# Prevalence and Characteristics Associated With Post–COVID-19 Condition Among Nonhospitalized Adolescents and Young Adults

**DOI:** 10.1001/jamanetworkopen.2023.5763

**Published:** 2023-03-30

**Authors:** Joel Selvakumar, Lise Beier Havdal, Martin Drevvatne, Elias Myrstad Brodwall, Lise Lund Berven, Tonje Stiansen-Sonerud, Gunnar Einvik, Truls Michael Leegaard, Trygve Tjade, Annika E. Michelsen, Tom Eirik Mollnes, Fridtjof Lund-Johansen, Trygve Holmøy, Henrik Zetterberg, Kaj Blennow, Carolina X. Sandler, Erin Cvejic, Andrew R. Lloyd, Vegard Bruun Bratholm Wyller

**Affiliations:** 1 Department of Paediatrics and Adolescent Health, Akershus University Hospital, Lørenskog, Norway; 2 Institute of Clinical Medicine, University of Oslo, Oslo, Norway; 3 Department of Clinical Molecular Biology (EpiGen), University of Oslo and Akershus University Hospital, Lørenskog, Norway; 4 Department of Pulmonary Medicine, Akershus University Hospital, Lørenskog, Norway; 5 Department of Microbiology and Infection Control, Akershus University Hospital, Lørenskog, Norway; 6 Fürst Medical Laboratory, Oslo, Norway.; 7 Research Institute of Internal Medicine, Oslo University Hospital, Oslo, Norway; 8 Department of Immunology, University of Oslo and Oslo University Hospital, Oslo, Norway; 9 Research Laboratory, Nordland Hospital, Bodø, Norway; 10 Centre of Molecular Inflammation Research, Norwegian University of Science and Technology, Trondheim, Norway; 11 Department of Neurology, Akershus University Hospital, Lørenskog, Norway; 12 Institute of Neuroscience and Physiology, Department of Psychiatry and Neurochemistry, Sahlgrenska Academy, University of Gothenburg, Mölndal, Sweden; 13 Clinical Neurochemistry Laboratory, Sahlgrenska University Hospital, Mölndal, Sweden; 14 UCL Institute of Neurology, Department of Neurodegenerative Disease, London, United Kingdom; 15 UK Dementia Research Institute, Gower Street, London, United Kingdom; 16 Hong Kong Center for Neurodegenerative Diseases, Hong Kong, China; 17 School of Health Sciences, Western Sydney University, Penrith, Australia; 18 The Kirby Institute, University of New South Wales, Sydney, Australia; 19 School of Public Health, Faculty of Medicine and Health, University of Sydney, Sydney Australia

## Abstract

**Importance:**

The prevalence and baseline risk factors of post–COVID-19 condition (PCC) remain unresolved among the large number of young people who experienced mild COVID-19.

**Objectives:**

To determine the point prevalence of PCC 6 months after the acute infection, to determine the risk of development of PCC adjusted for possible confounders, and to explore a broad range of potential risk factors.

**Design, Setting, and Participants:**

This cohort study included nonhospitalized individuals from 2 counties in Norway between ages 12 and 25 years who underwent reverse transcription–polymerase chain reaction (RT-PCR) testing. At the early convalescent stage and at 6-month follow-up, participants underwent a clinical examination; pulmonary, cardiac, and cognitive functional testing; immunological and organ injury biomarker analyses; and completion of a questionnaire. Participants were classified according to the World Health Organization case definition of PCC at follow-up. Association analyses of 78 potential risk factors were performed.

**Exposures:**

SARS-CoV-2 infection.

**Main Outcomes and Measures:**

The point prevalence of PCC 6 months after RT-PCR testing in the SARS-CoV-2–positive and SARS-CoV-2–negative groups, and the risk difference with corresponding 95% CIs.

**Results:**

A total of 404 individuals testing positive for SARS-CoV-2 and 105 individuals testing negative were enrolled (194 male [38.1%]; 102 non-European [20.0%] ethnicity). A total of 22 of the SARS-CoV-2–positive and 4 of the SARS-CoV-2–negative individuals were lost to follow-up, and 16 SARS-CoV-2–negative individuals were excluded due to SARS-CoV-2 infection in the observational period. Hence, 382 SARS-CoV-2–positive participants (mean [SD] age, 18.0 [3.7] years; 152 male [39.8%]) and 85 SARS-CoV-2–negative participants (mean [SD] age, 17.7 [3.2] years; 31 male [36.5%]) could be evaluated. The point prevalence of PCC at 6 months was 48.5% in the SARS-CoV-2–positive group and 47.1% in the control group (risk difference, 1.5%; 95% CI, −10.2% to 13.1%). SARS-CoV-2 positivity was not associated with the development of PCC (relative risk [RR], 1.06; 95% CI, 0.83 to 1.37; final multivariable model utilizing modified Poisson regression). The main risk factor for PCC was symptom severity at baseline (RR, 1.41; 95% CI, 1.27-1.56). Low physical activity (RR, 0.96; 95% CI, 0.92-1.00) and loneliness (RR, 1.01; 95% CI, 1.00-1.02) were also associated, while biological markers were not. Symptom severity correlated with personality traits.

**Conclusions and Relevance:**

The persistent symptoms and disability that characterize PCC are associated with factors other than SARS-CoV-2 infection, including psychosocial factors. This finding raises questions about the utility of the World Health Organization case definition and has implications for the planning of health care services as well as for further research on PCC.

## Key Points

**Question:**

What are the prevalence and associated risk factors of post–COVID-19 condition (PCC) in young people after mild acute infection?

**Findings:**

This cohort study included 382 SARS-CoV-2–positive individuals and a control group of 85 SARS-CoV-2–negative individuals aged 12 to 25 years who were assessed at the early convalescent stage and at 6-month follow-up. When applying the World Health Organization case definition of PCC, prevalence at 6 months was 49%, but was also comparably high (47%) in the control group. PCC was not associated with biological markers specific to viral infection, but with initial symptom severity and psychosocial factors.

**Meaning:**

These findings suggest that persistent symptoms in this age group are related to factors other than SARS-CoV-2 infection, and therefore question the usefulness of the WHO case definition of PCC.

## Teaser

This cohort study of Norwegian adolescents and young adults with mild SARS-CoV-2 infections examines the prevalence of post–COVID-19 condition 6 months after infection.

## Introduction

Post–COVID-19 condition (PCC) is characterized by the persistence of symptoms such as fatigue, dyspnea, and what is commonly referred to as “brain fog” occurring 3 months or longer after infection with SARS-CoV-2.^1^ The prevalence remains uncertain, with a review of PCC symptoms in children and adolescents reporting fatigue rates between 3% and 87%, whereas a meta-analysis reported the confidence interval of fatigue prevalence to be 32% to 62%.^2,3^

When sequelae arise after mild acute infection, a subset of cases might fit the label of postinfective fatigue syndrome (PIFS), in which persistent symptoms and disability accompany scarce findings on standard clinical examination.^4,5,6,7^ In the aftermath of a wide array of infectious diseases, such as mononucleosis, Q fever, and giardiasis, multiple prospective cohort studies report that 10% to 15% of patients experience moderate to severe disability meeting the diagnostic criteria for PIFS, in line with current studies of PCC.^6,7,8,9,10,11,12^

The underlying disease mechanisms of PCC, as well as PIFS, remain elusive. For PIFS, suggested explanations range from low-grade inflammation to functional alterations of the brain’s perception of bodily states partly caused by psychosocial factors.^13,14^ Most studies of PCC have focused on infection-specific factors (what may be considered as direct factors), such as immunological aberrations, and other possible mechanisms—organ damage, endotheliopathy, persisting viral reservoirs, and autoimmune inflammation have been proposed.^6,15,16,17,18,19^ However, indirect, nonspecific stressors during the pandemic, such as fear of viral transmission, societal lockdown, and parents experiencing PCC, have also been suggested.^6,20,21,22,23,24^

Studies of PIFS have benefitted from an international case definition^25^ that is centered around the symptom of fatigue, which should be persistent from onset of the acute infectious event, severely affect daily activities, and not be caused by any other condition; diagnosed individuals must experience at least 4 of 8 additional symptoms (such as headache and concentration or memory problems). In contrast, the broad case definition of PCC established by the World Health Organization (WHO) encompasses any symptom occurring in the aftermath of acute COVID-19, does not require symptom persistence since the infectious event, and does not stipulate significant disability.^1^

Prospective studies of nonhospitalized patients with COVID-19 with contemporaneous, SARS-CoV-2-antibody–negative control participants are scarce in younger age groups.^4,6,26^ To the best of our knowledge, no previous reports have provided prevalence estimates for PCC based upon a rigorous evaluation of caseness, including the assessment of alternative medical and psychiatric diagnoses. Furthermore, few studies have investigated both direct disease-specific factors, such as immunological activation markers, and indirect general stressors.^11,19,27,28^ Hence, the aims of this prospective controlled cohort study of nonhospitalized adolescents and young adults were 3-fold: (1) to determine the point prevalence of PCC in the SARS-CoV-2–positive group according to the WHO and PIFS definitions 6 months after acute COVID-19, while as a control measure applying the case definitions to the SARS-CoV-2–negative group as well; (2) to determine the risk of development of PCC 6 months after acute COVID-19, adjusted for possible confounders; and (3) to explore a broad range of potential risk factors for PCC.

## Methods

The current paper follows the reporting guidelines of Strengthening the Reporting of Observational Studies in Epidemiology (STROBE). The project was approved by the Regional Committee for Ethics in Medical Research and given a limited confidentiality waiver allowing us to approach individuals eligible for recruitment by text message. Written informed consent was obtained as required by the Norwegian Health Research Act.

### Study Design

This was a prospective cohort study of adolescents and young adults testing positive and negative for SARS-CoV-2 who were not hospitalized, with follow-up 6 and 12 months after inclusion (ClinicalTrials.gov identifier: NCT04686734) (Figure; eMethods in Supplement 1). Selected baseline data have been reported elsewhere.^29^ Data from 12-month follow-up are not presented in the present report.

**Figure.**
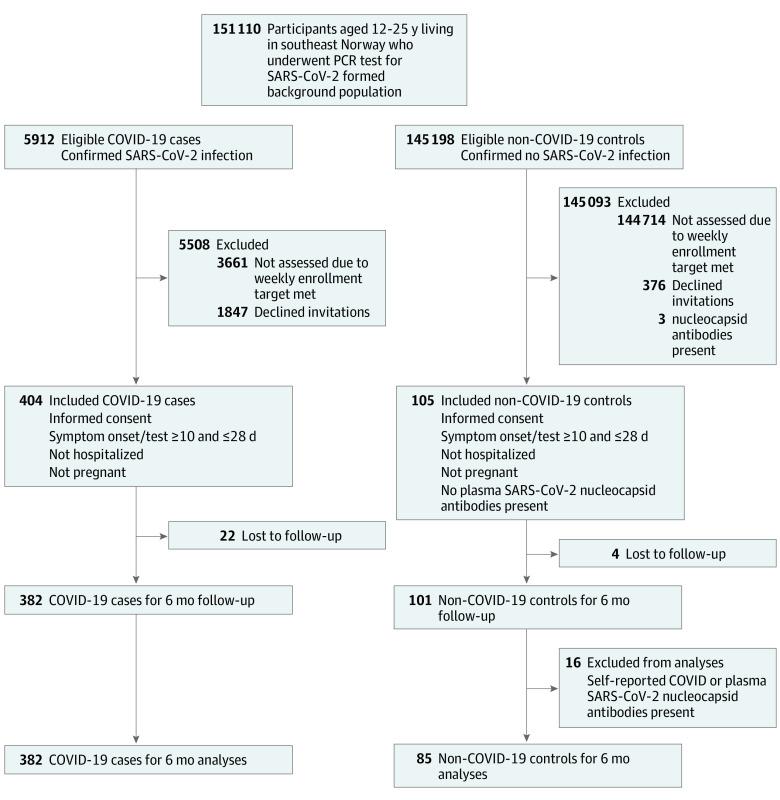
Study Flowchart

### Participants

From December 24, 2020, until May 18, 2021, consecutive individuals aged 12 to 25 years undergoing SARS-CoV-2 testing in 2 accredited microbiological laboratories in southeast Norway (Fürst Medical Laboratories and the Department of Microbiology and Infection Control at Akershus University Hospital) were recruited. The B.1.1.7 (Alpha) variant of SARS-CoV-2 was dominant in the geographical area during most of the recruitment period. Individuals with laboratory-confirmed SARS-CoV-2 infection (detected by upper respiratory tract swabs followed by reverse transcription–polymerase chain reaction [RT-PCR]) were eligible for enrollment after completing 10-days quarantine (SARS-CoV-2–positive group). Individuals having approximately the same distribution of sex and age as the SARS-CoV-2–infected cases, but with a negative SARS-CoV-2 test were recruited as controls (SARS-CoV-2–negative group). Some individuals in the SARS-CoV-2–negative group had been tested because of acute infectious symptoms, others were asymptomatic close contacts. Exclusion criteria at baseline were having greater than 28 days since onset of symptoms or SARS-CoV-2 test, hospitalization due to COVID-19, pregnancy, and having serological evidence of previous COVID-19 infection (in the SARS-CoV-2–negative group). In addition, SARS-CoV-2–negative individuals with evidence of SARS-COV-2 infection at follow-up (acute COVID-19 in the observational period or the presence of antinucleocapsid antibodies at follow-up) were excluded from 6-month analyses.

### Assessment Program and Clinical Examination

At inclusion and follow-up, participants attended a 1-day investigational program at our study center at Akershus University Hospital, Norway, encompassing a clinical interview and complete physical examination, recording of vital signs, functional testing, blood sampling, and completion of questionnaires (eMethods in Supplement 1).

### Functional Testing

The forced vital capacity and the forced expiratory volume in 1 second were measured by standard spirometry. A 5-minute supine electrocardiogram recording was used to calculate heart rate variability indices, including power in the high-frequency (a marker of parasympathetic activity) and low-frequency (a marker of combined sympathetic and parasympathetic activity) range (eMethods in Supplement 1). Cognitive function tests included the digit span test of working memory and tests of verbal learning, recall, and recognition.

### Blood Sampling and Laboratory Assays

Samples were obtained by antecubital venous puncture. Immune markers assayed in plasma included C-reactive protein; growth/differentiation factor 15; terminal complement complex; regulated upon activation T cell expressed and secreted; monocyte chemotactic protein; interferon-inducible protein; immunoglobulins G, M, and A; interleukins 1β, 2, 4, 7, 8, 9, 12p70, 13, and 17a; tumor necrosis factor α; interferon-γ; eotaxin-1; macrophage inflammatory proteins 1α and 1β; granulocyte-macrophage colony-stimulating factor; basic fibroglast growth factor 2; and C3bc (an activation product of complement 3).

Antibodies against SARS-CoV-2 (antinucleocapsid and antireceptor binding domain), as well as Epstein-Barr virus (EBV) were assayed in serum to document recent or previous infection with these pathogens. Neurofilament light chain (NfL) and glial fibrillary acidic protein were assayed in serum, providing markers of brain axonal damage and astrocytic activation, respectively. Routine blood analyses of hematology and biochemistry (including D-dimer and the cardiac markers troponin T and N-terminal prohormone of brain natriuretic peptide) were carried out.

### Questionnaires

A composite questionnaire charted comorbidities, the family history of disease, current medication, smoking habits, substance abuse, physical activity, parents’ occupation, and history of COVID-19. Parents’ occupations were used as an indicator of socioeconomic status.

Clinical symptoms of fatigue, postexertional malaise, sleep problems, pain, anxiety, depression, and negative affect were recorded using validated inventories. A symptom inventory specifically designed for PIFS research was incorporated; sum scores were calculated for cognitive, respiratory, and autonomic symptoms, respectively.

The psychological traits of neuroticism, emotional awareness, worrying tendencies, and body vigilance were charted by validated inventories, as were quality of life and the social variables loneliness and significant life events. Information on vaccination was obtained through linkage with the Norwegian Immunisation Register.^30^

### Caseness Assessment

Application of the WHO definition of PCC and the case definition for PIFS at 6 months was operationalized, and all participants were classified as cases or noncases according to both definitions. A distinction was made between certain and uncertain classification based upon a detailed assessment of other conditions (eg, medical or psychiatric comorbidity) that may explain symptoms. This assessment was performed independently by 2 researchers masked to initial SARS-CoV-2 status.

### Risk Factor Hypotheses

The scientific literature on PCC, as well as PIFS, was scrutinized to identify potential baseline risk factors of 6-month caseness. A total of 78 variables were identified, grouped as: SARS-CoV-2 status (positive vs negative), background and constitutional factors (sex, age, body mass index, ethnicity, chronic disorders), observational period characteristics (vaccinations, duration from baseline to follow-up), organ function tests and biomarkers, immunological markers, autonomic markers, cognitive function tests, clinical symptoms, psychological traits, and social and behavioral markers. SARS-CoV-2 status was hypothesized to be the main risk factor for PCC, as well as for PIFS. Background and constitutional factors and observational period characteristics were regarded potential confounders. The remaining variables were assumed to be either mediating variables related to COVID-19 pathophysiology or independent variables.

### Statistical Analyses

PCC and PIFS at 6 months were defined as primary and secondary outcomes, respectively. The study had a power of approximately 80% to detect a relative risk (RR) of 1.5.

Prevalence data are reported separately in the SARS-CoV-2–positive and SARS-CoV-2–negative groups, and the risk difference calculated. For analyses of risk factors, bivariate analyses between the 2 outcome variables and each hypothesized risk factor were performed by generalized linear modeling using a modified Poisson approach (log-link and robust error variances). Dimensionality reduction was performed by principal component analyses (PCA). SARS-CoV-2 status, background and constitutional factors, observational period characteristics and all remaining variables with an unadjusted *P* < .25 were included in a multivariable model; variables were then removed and eventually reinserted 1 by 1 dependent on their influence on overall goodness-of-fit in order to find the most parsimonious model.

As sensitivity analyses, identical analytical procedures were performed on 2 different data sets: 1 with imputation of missing data points with mean or median values, and 1 with exclusion of participants with uncertain caseness classification, vaccination prior to enrolment or less than 5 days prior to follow-up appointment, or evidence of recent EBV infection at enrollment or during the observational period. An additional sensitivity analysis of the final multivariable model was performed where individuals in the SARS-CoV-2–negative group with baseline symptoms suggesting an acute infection were removed alongside the exclusions listed above. *P* < .05 was considered statistically significant in 2-sided tests. All statistical analyses were carried out in SPSS version 28.0 (SPSS Inc).

## Results

A total of 151 110 RT-PCR tests of SARS-CoV-2 were carried out in the background population during the recruitment period (Figure). A total of 5912 individuals (3.9%) were SARS-CoV-2–positive, of whom 2251 (1136 male [50.5%]) were invited into the study. Of this group 404 (mean [SD] age, 18.1 [3.7] years; 157 male [38.9%]) were enrolled (Table 1; eTable 1 in Supplement 1). Among the individuals in the SARS-CoV-2–negative control group, a total of 484 were invited (330 males [68.2%]), while 105 were enrolled (mean [SD] age, 17.6 [3.3] years; 37 male [35.2%]), and their negative status was confirmed by the absence of antinucleocapsid antibodies at baseline. The invited sample had a distribution of sex and age similar to the background population; however, within the enrolled sample, the group aged 18 to 25 years had a disproportionately higher number of female participants (eTable 2 in Supplement 1). The SARS-CoV-2–positive group had a higher proportion of individuals with non-European ethnicity than the SARS-CoV-2–negative group; otherwise, the 2 groups were comparable (Table 1).

**Table 1.** Cohort Characteristics at Baseline and 6 Month Follow-up

Characteristic	Participants, No. (%)					
	Inclusion			6-mo follow-up		
	SARS-CoV-2		Missing values	SARS-CoV-2		Missing values
	Positive group (n = 404)	Negative group (n = 105)		Positive group (n = 382)	Negative group (n = 85)	
Background						
Sex						
Female	247 (61.1)	68 (64.8)	0	230 (60.2)	54 (63.5)	0
Male	157 (38.9)	37 (35.2)		152 (39.8)	31 (36.5)	
Age group						
12-15 y	101 (25.0)	25 (23.8)	0	98 (25.7)	18 (21.2)	0
15-18 y	107 (26.5)	35 (33.3)		104 (27.2)	31 (36.5)	
18-21 y	84 (20.8)	26 (24.8)		80 (20.9)	21 (24.7)	
21-25 y	112 (27.7)	19 (18.1)		100 (26.2)	15 (17.6)	
BMI, mean (SD), z-score^a^	0.45 (1.2)	0.49 (1.1)	0	0.52 (1.2)	0.51 (1.1)	0
Ethnicity						
European	306 (75.7)	101 (96.2)	0	294 (77.0)	83 (97.6)	0
Non-European	98 (24.3)	4 (3.8)		88 (23.0)	2 (2.4)	
Current comorbidity						
Any comorbidity	81 (21)	36 (35)	14 (2.8)	89 (24)	31 (37)	13 (2.8)
ADHD	5 (1.3)	3 (2.9)		5 (1.4)	3 (3.6)	
Asthma	26 (6.7)	5 (4.8)		27 (7.3)	4 (4.8)	
Allergy and atopy	16 (4.1)	10 (9.6)		17 (4.6)	9 (11)	
Anxiety and depression	1 (0.3)	3 (2.9)		4 (1.1)	3 (3.6)	
Endocrinological	6 (1.5)	1 (1.0)		6 (1.6)	1 (1.2)	
Gastrointestinal	5 (1.3)	4 (3.8)		6 (1.6)	5 (6.0)	
Gynecological	4 (1.0)	1 (1.2)		4 (1.0)	1 (1.2)	
Neurological including primary headache disorders	10 (2.6)	5 (4.8)		9 (2.5)	4 (2.8)	
Socieconomic level						
Parents’ highest ISEI-08 score (range, 10-90), median (IQR)	63 (21)	65 (17)	48 (9.4)	64 (21)	62 (18)	44 (9.4)
Smoking						
Daily	1 (0.3)	0	16 (3.1)	1 (0.3)	0	16 (3.4)
Never	376 (96.7)	101 (97.1)		355 (96.7)	82 (97.6)	
COVID-19 immunization, doses						
None	399 (98.8)	99 (94.3)	0	145 (38.0)	8 (9.4)	0
1	5 (1.2)	4 (3.8)		232 (60.7)	29 (34.1)	
2	0	2 (1.9)		5 (1.3)	47 (55.3)	
3	0	0		0	1 (1.2)	
Symptoms and functional impairment scores^b^						
Fatigue (range, 0-33), mean (SD)^c^	16.2 (5.7)	13.2 (4.7)	16 (3.1)	14.5 (5.2)	13.3 (3.8)	0
Postexertional malaise (range, 0-100), median (IQR)^d^	20.0 (5.0-45.0)	10 (1.3-25.0)	16 (3.1)	10.0 (0-35)	10.0 (0-22.5)	3 (0.6)
Cognitive symptoms (range, 3-15), median (IQR)^e^	6.0 (3.0-8.5)	6.0 (4.3-9.0)	16.0 (3.1)	6.0 (4.0-10.0)	6.0 (4.0-8.0)	3 (0.6)
Respiratory symptoms (range, 2-10), median (IQR)^f^	4.0 (3.0-6.0)	3.0 (2.0-4.0)	16 (3.1)	3.0 (2.0-5.0)	3.0 (3.0-4.0)	3 (0.6)
Symptoms of anxiety (range, 0-21), median (IQR)^g^	5.0 (3.0-9.0)	7.0 (4.0-10.0)	16 (3.1)	6.0 (3.0-9.0)	5.0 (3.5-10.0)	3 (0.6)
Symptoms of depression (range, 0-21), median (IQR)^h^	3.0 (1.0-6.0)	3.5 (2.0-6.0)	16 (3.1)	3.0 (1.0-6.0)	3.0 (1.0-7.0)	3 (0.6)
Quality of life (range, 0-100), median (IQR)^i^	77.2 (63.6-88.0)	77.2 (65.2-84.8)	16 (3.1)	78.3 (66.3-88.0)	76.1 (67.9-86.4)	3 (0.6)
Clinical findings						
Time since symptom onset/PCR test, median (IQR), d	18.0 (15.0-21.0)	17 (14-21)	0	213 (207-224)	210.0 (205.0-218.5)	0
Time between baseline and follow-up, median (IQR), d	NA	NA	NA	193.0 (188.0-205.0)	193 (188.0-200.0)	0
Tympanic temperature, mean (SD), °C	36.8 (0.4)	36.7 (0.4)	2 (0.4)	36.6 (0.4)	36.7 (0.4)	0
SpO_2_, mean (SD), %	98.6 (1.1)	98.6 (1.2)	2 (0.4)	98.5 (1.1)	98.3 (1.3)	0
FVC, mean (SD), % of estimated value^j^	99.5 (10.0)	100.4 (10.3)	67 (14.3)	99.5 (10.3)	99.9 (9.9)	45 (9.6)
Laboratory findings						
Blood, mean (SD)						
Hemoglobin, g/dL	13.5 (1.2)	13.5 (1.1)	43 (8.4)	13.6 (1.2)	13.7 (1.0)	13 (2.8)
Leukocyte count, 10^9^ cells/L	5.9 (1.5)	5.6 (1.3)	44 (8.6)	6.1 (1.8)	5.9 (1.5)	13 (2.8)
Plasma, median (IQR), mg/L						
hsCRP	0.8 (0.4-2.6)	1.3 (0.5-3.5)	18 (3.6)	1.3 (0.45-4.24)	1.8 (0.7-5.7)	9 (1.9)
D-dimer	0.2 (0.1-0.3)	0.2 (0.1-0.3)	12 (2.4)	0.1 (0.1-0.2)	0.1 (0.1-0.2)	9 (1.9)
Serum, median (IQR)						
NT-proBNP, ng/L	34.5 (21.0-57.3)	34.0 (20.5-54.5)	30 (5.9)	30.0 (19.0-49.0)	34.0 (19.5-54.5)	14 (3.0)
Troponin T, ng/L	4.0 (2.4-6.0)	4.0 (1.5-4.0)	23 (4.5)	2.1 (1.1-4.0)	2.1 (0.8-4.0)	14 (3.0)
SARS-CoV-2 antibody titer^k^	4.0 (0.9-14.9)	0 (0-0)	13 (2.6)	23 (6.9-51.1)	0.1 (0.1-0.1)	11 (2.4)

Abbreviations: ADHD, attention-deficit/hyperactivity disorder; BMI, body mass index; FVC, forced vital capacity; hsCRP, high-sensitive assay of C-reactive protein; ISEI, international socioeconomic index; NA, not applicable; NT-proBNP, N-terminal pro-brain natriuretic peptide; PCR, polymerase chain reaction; SpO_2_, peripheral oxygen saturation.

SI conversion factor: To convert D-dimer to nanomole per liter, multiply by 5.476; hemoglobin to grams per liter, multiply by 10; troponin T to micrograms per liter, multiply by 0.001.

^a^ Standardized score calculated according to World Health Organization 2006 Child Growth Standards.

^b^ With the exception of quality of life, higher values imply more symptoms. For quality of life, higher values imply higher quality of life and less functional impairment.

^c^ From the Chalder Fatigue Questionnaire (eMethods in Supplement 1).

^d^ From the DePaul Symptom Questionnaire (eMethods in Supplement 1).

^e^ The sum score across the 3 items memory problems, concentration problems, and decision-making problems (eMethods in Supplement 1).

^f^ The sum of scores across dyspnea and coughing (eMethods in Supplement 1).

^g^ From the Hospital Anxiety and Depression Scale anxiety subscale (eMethods in Supplement 1).

^h^ From the Hospital Anxiety and Depression Scale depression subscale (eMethods in Supplement 1).

^i^ From the Pediatric Quality of Life Inventory (eMethods in Supplement 1).

^j^ The Global Lung Function Initiative 2012 reference values were used to calculate estimated values (eMethods in Supplement 1).

^k^ Total antinucleocapsid immunoglobulin G and M.

A total of 22 individuals (5.4%) in the SARS-CoV-2–positive group and 4 (3.8%) in the SARS-CoV-2–negative group were lost to follow-up (Figure; eTable 3 in Supplement 1). Additionally, 16 individuals were excluded from the SARS-CoV-2–negative group at follow-up due to evidence of SARS-CoV-2 infection, leaving 382 in the SARS-CoV-2–positive group and 85 in the SARS-CoV-2–negative group for evaluation at 6 months (Figure). The SARS-CoV-2–positive group had received fewer immunization doses in the observational period; otherwise, the 2 groups remained comparable (Table 1). Missing data points for the independent variables were randomly distributed and the median (IQR) missingness per variable was 3.4% (3.3%-3.4%; range, 0%-14.3%), while 160 individuals (34.2%) had missing values for at least 1 variable (eTable 4 in Supplement 1). Additionally, 3 individuals in the SARS-CoV-2–positive group had missing questionnaire data at 6 months, and thus were excluded from prevalence and regression analyses. A total of 10 individuals (2.7%) in the SARS-CoV-2–positive group and 3 individuals (3.6%) in the SARS-CoV-2–negative group had a serological pattern suggesting recent EBV infection prior to enrolment or during the observational period (eTables 5 and 6 in Supplement 1).

At 6 month follow-up, 184 of 379 individuals in the SARS-COV-2–positive group and 40 of 85 individuals in the SARS-CoV-2–negative group were classified as having PCC (eFigure 1, eTable 7 in Supplement 1), respectively corresponding to almost identical point prevalences of 48.5% (95% CI, 43.6% to 53.6%) and 47.1% (95% CI, 36.8% to 57.6%), for a risk difference of 1.5% (95% CI, −10.2% to 13.1%). For PIFS, 53 individuals in the SARS-CoV-2–positive group and 7 individuals in the SARS-COV-2–negative group met the criteria (eFigure 2 in Supplement 1), corresponding respectively to a point prevalence of 14.0% (95% CI, 10.8% to 17.9%) and 8.2% (95% CI, 3.8% to 16.3%), for a risk difference of 5.7% (95% CI, −2.0% to 12.0%). For the majority of individual symptoms, the confidence intervals of the prevalence overlapped between the groups; however, some dimensions of fatigue and ear-nose-throat symptoms were more common in the SARS-CoV-2–positive group (eTable 12 in Supplement 1).

SARS-CoV-2 status was not associated with either PCC or PIFS at 6 months (Table 2). PCA of clinical symptoms and psychological traits extracted 1 main component from each of these 2 variable groups (eTables 13 through 15 in Supplement 1). These components, representing symptom severity and emotional maladjustment, were strongly associated with both PCC and PIFS in bivariate regression analyses (eTable 16 in Supplement 1). Other notable risk factors at baseline for both conditions were female sex, low self-reported level of physical activity before infection, loneliness, and negative life events during the preceding year. The majority of biological markers were not associated with the outcome variables (eTable 16 in Supplement 1).

**Table 2.** Baseline Risk Factors of Post–COVID-19 Condition (PCC) and the Postinfective Fatigue Syndrome (PIFS) at 6-month Follow-up^a^

Characteristic	PCC^b^		PIFS^c^	
	Relative risk (95% CI)^d^	*P* value^e^	Relative risk (95% CI)^d^	*P* value^e^
SARS-CoV-2 status				
Positive at baseline	1.06 (0.83-1.37)	.66	1.63 (0.86-3.36)	.14
Background and constitutional factors				
Female sex	1.16 (0.94-1.44)	.16	1.50 (0.86-2.78)	.16
Age, y	0.98 (0.95-1.00)	.09	1.03 (0.97-1.09)	.33
BMI, z-score^f^	1.00 (0.92-1.08)	.97	0.86 (0.72-1.03)	.10
Non-European ethnicity	0.95 (0.75-1.20)	.69	0.97 (0.59-1.57)	.92
Any comorbidity	1.10 (0.89-1.36)	.36	0.79 (0.49-1.25)	.32
Observational period characteristics				
Time span between baseline and follow-up, d	1.00 (1.00-1.01)	.70	0.99 (0.98-1.00)	.11
Immunization against COVID-19^g^	0.80 (0.32-1.67)	.59	2.40 (0.66-6.64)	.17
Remaining risk factors				
Symptom severity^h^	1.41 (1.27-1.56)	<.001	3.37 (2.72-4.20)	<.001
Physical activity prior to infection^i^	0.96 (0.92-1.00)	.03	NA	NA
Loneliness^j^	1.01 (1.00-1.02)	.01	NA	NA
Blood lymphocyte count	NA	NA	0.68 (0.48-0.94)	.02
Plasma IL-7	NA	NA	0.97 (0.95-0.99)	.006
Negative life events prior to last year^k^	NA	NA	0.88 (0.80-0.96)	.004
LF-RRI^l^	NA	NA	0.66 (0.53-0.82)	<.001

Abbreviations: BMI, body mass index; LF-RRI, low-frequency power of heart rate variability; PCC, post–COVID-19 condition.

^a^ The analyses encompassed a total of 464 individuals. Three individuals belonging to the SARS-CoV-2–positive group at baseline had missing values in questionnaire data at 6 months precluding classification according to the case definitions; hence, they were removed from regression analyses. Final multiple regression models of per protocol data (modified Poisson regression with log-link and robust error variances).

^b^ According to the World Health Organization definition of PCC.^1^

^c^ According to the international case definition of PIFS.^25^

^d^ 95% profile likelihood-based confidence intervals.

^e^ Likelihood ratio *P* values.

^f^ Standardized score calculated according to World Health Organization 2006 Child Growth Standards.

^g^ One or more doses of COVID-19 vaccine.

^h^ Component extracted from principal component analysis of 10 clinical symptoms variables at baseline; higher value implies more severe symptoms.

^i^ From a single questionnaire item; higher scores imply more physically active.

^j^ From the University of California, Los Angeles loneliness questionnaire; higher scores imply more loneliness (eMethods in Supplement 1).

^k^ From the life events checklist impact score; higher scores imply more subjective negative impact (eMethods in Supplement 1).

^l^ Log-transformed variable was used for regression analysis.

In the final multivariable model, the symptom severity component remained the main risk factor, both for PCC (RR, 1.41; 95% CI, 1.27 to 1.56), and for PIFS (RR, 3.37; 95% CI, 2.72 to 4.20) (Table 2). Additionally, loneliness and low levels of physical activity were associated with PCC. The symptom severity component correlated with the emotional maladjustment component and with female sex, explaining why the latter 2 variables were not associated with the outcome in the multivariable modeling (eFigure 3 in Supplement 1). The sensitivity analyses yielded comparable results for prevalence estimates, bivariate regression analyses and multivariable modeling (eTables 8 through 11 and eTables 17 through 21 in Supplement 1).

## Discussion

The main results from the present study were: (1) the prevalence of PCC 6 months after acute COVID-19 was approximately 50%, but was equally high in a control group of comparable SARS-CoV-2–negative individuals; (2) acute COVID-19 was not an independent risk factor for PCC; (3) the severity of clinical symptoms at baseline, irrespective of SARS-CoV-2 status, was the main risk factor of persistent symptoms 6 months later.

Symptom prevalence data are consistent with other controlled studies of young people after acute COVID-19 reporting a high symptom load, with only subtle differences between individuals testing positive and negative for SARS-CoV-2.^3,26,31,32,33^ Correspondingly, a large population-based study found no associations between most persistent symptoms attributed to COVID-19 and serological evidence of SARS-CoV-2 infection.^34^

Hence, mild acute COVID-19 per se does not seem to be the main driver of most persistent symptoms in this age group. Rather, 2 other phenomena might be affecting these results: first, symptoms associated with PCC are common in the general population. For instance, the point prevalence of fatigue was reported to be 34% to 38% among British adolescents,^35^ with similar high rates for symptoms such as dyspnea and memory problems.^36^ Second, several studies have documented a significant increase in mental distress in the general population during the pandemic,^37^ particularly affecting young people,^38,39^ which in turn may affect physical symptoms.^22,27^ Hence, nonspecific stressors either unique to or increasing during the pandemic, and which affected both SARS-CoV-2–positive and SARS-CoV-2–negative individuals similarly, may be important for symptom persistence and associated disability. This possibility should be considered when societal countermeasures against infection outbreaks such as lockdowns are implemented.

The association of baseline symptom severity with symptom persistence echoes previous findings from studies of both PCC and PIFS,^6,9,10,40,41^ as well as general studies of clinical symptoms.^35^ Of particular relevance, Wessely et al^42^ reported that common, mild acute infections in general practice were not associated with PIFS, whereas fatigue and psychosocial distress prior to presentation with a clinical infection were strongly associated. In the present study, severity of baseline clinical symptoms was associated with female sex and psychological traits. These associations might be important for the understanding of persistent symptoms in general, and deserve attention in future studies.

In contrast to previous reports on PCC as well as PIFS,^10,11,19,43^ no immune markers were associated with symptom persistence in the present study. This may be seen as a logical consequence of the absence of association between the outcomes and SARS-CoV-2-status. Unaltered concentrations of central nervous system injury markers in blood in the PCC group speak against ongoing neuronal injury and astrocytic activation.

The prevalence of PIFS in the current study was comparable with observations from studies of sequelae after other infections,^8,9,10,44,45^ and also yielded a nonsignificant trend toward a higher prevalence in the SARS-CoV-2–positive group. Furthermore, certain dimensions of fatigue (eg, postexertional malaise) were more common in the SARS-CoV-2–positive group. These observations suggest that further analysis of the phenomenon of fatigue following COVID-19 might be of value.

### Strengths and Limitations

Strengths of this study included rigorous case definitions and evaluation tools, comprehensive risk factor assessment, a well-defined control group, and a low dropout rate. However, there were several limitations. The low number of individuals in the control group reduced statistical power. For the sensitivity analysis, we opted to use a crude method of mean and median imputation, rather than multiple imputation. Given the sparsity of missingness per variable, we believe it unlikely that a more complex imputation approach would appreciably alter the outcome. Regarding internal validity, a correction for pre–COVID-19 symptoms might diminish the estimated prevalence.^46^ A limitation to external validity, shared with similar studies in nonhospitalized individuals, is that our study was prone to self-selection bias. We cannot rule out that our sample was skewed with regards to what we have described as indirect stressors, ie, that individuals who chose to enroll in the control group had more symptoms than the background population. Furthermore, it is unclear to what extent the results of the present study are applicable to those with more severe acute COVID-19, as persistent symptoms seem to be more common and have been found to be associated with other risk factors in hospitalized patients.^47,48,49^ Also, the present study included only young people, with the great majority infected with the Alpha variant of SARS-CoV-2, hence the generalizability to older age groups and other viral variants is uncertain. Given the potential importance of external factors influencing symptom persistence, studies across different cultural contexts might yield different results. Finally, while the present study showed no association between SARS-CoV-2 and the WHO case definition of PCC, SARS-CoV-2 may still be a risk factor for other diagnostic entities.

## Conclusions

The 6-month point prevalence of PCC was similar in infected and noninfected individuals, thus questioning the usefulness of the WHO case definition. Symptom severity at baseline was the main risk factor, and correlated with personality traits. Low physical activity and loneliness were also associated with the outcome. These results suggest that factors often labeled as psychosocial should be considered risk factors for persistent symptoms. This does not imply that PCC is “all in the mind,” or that the condition has a homogeneous, psychological etiology. Rather, there might be heterogeneous biological, psychological, and social factors engaged in triggering and maintaining the symptoms of the individual.^50^ However, the results do suggest that nonpharmacological interventions may be beneficial and should be investigated in future studies, in line with experiences from PIFS following other infections.^51^

## Notes

Supplement 1.
eMethods.
**eTable 1.** Results of All SARS-CoV-2 PCR Tests Performed Between December 24, 2020 and May 18, 2021 at Akershus University Hospital and Fürst Medical Laboratory, With Respect to Age and Sex **eTable 2.** Attritional Analyses: SARS-CoV-2–positive in Background Population, Proportions Invited to Participate, and Proportions Included in Study, With Respect to Age and Sex **eTable 3.** Attritional Analyses: Characteristics of Potential Baseline Risk Factors and Their Univariate Associations (Poisson Regression With Log-Link and Robust Error Variances) to Being Lost to Follow-up **eTable 4.** Analyses of Missing Data: Characteristics of Baseline Independent Variables and Their Association to Complete Cases at Six Months Follow-up **eTable 5.** Results of Epstein-Barr Virus (EBV) Serology at Baseline and Six Months Follow-up **eTable 6.** Epstein-Barr Virus (EBV) Infection Status of Individuals Attending Six Month Follow-up **eTable 7.** Point Prevalence Percentage of Long COVID-19 and Postinfective Fatigue Syndrome at Six Months Follow-up **eTable 8.** Point Prevalence (Confidence Intervals) of Long COVID-19 at Six Months Follow-up: Sensitivity Analysis Removing Cases of Uncertain Classification, Individuals With Possible EBV Infection at Inclusion or During the Observational Period, Individuals Vaccinated Before Baseline and Individuals Receiving Vaccination Less Than Five Days Prior to the Six Months Assessment **eTable 9.** Point Prevalence Percentage (Confidence Intervals) of Long COVID-19 at Six Months Follow-up: Sensitivity Analysis Removing Cases of Uncertain Classification, Individuals With Possible EBV Infection at Inclusion or During the Observational Period, Individuals Vaccinated Before Baseline, Individuals Receiving Vaccination Less Than Five Days Prior to the Six Month Assessment, and Individuals in the SARS-CoV-2 Negative Group With General Infectious Symptoms Score ≥11 at Baseline **eTable 10.** Point Prevalence Percentage (Confidence Intervals) of Postinfective Fatigue Syndrome (PIFS) at Six Months Follow-up: Sensitivity Analysis Removing Cases of Uncertain Classification, Individuals With Possible EBV Infection at Inclusion or During the Observational Period, Individuals Vaccinated Before Baseline and Individuals Receiving Vaccination Less Than Five Days Prior to the Six Months Assessment **eTable 11.** Point Prevalence Percentage (Confidence Intervals) of Postinfective Fatigue Syndrome (PIFS) at Six Months Follow-up: Sensitivity Analysis Removing Cases of Uncertain Classification, Individuals With Possible EBV Infection at Inclusion or During the Observational Period, Individuals Vaccinated Before Baseline and Individuals Receiving Vaccination Less Than Five Days Prior to the Six Months Assessment, and Individuals in the SARS-CoV-2–negative Group With General Infectious Symptoms Score ≥11 at Baseline **eTable 12.** Point Prevalence Percentage (Confidence Intervals) of Specific Symptoms at Baseline and Six Months Follow-up **eTable 13.** Results of Final Factor Analyses (Principal Component Analysis) of Ten Clinical Symptoms Variables and Four Psychological Traits Variables, Respectively—Per Protocol Data **eTable 14.** Results of Final Factor Analyses (Principal Component Analysis) of Ten Clinical Symptoms Variables and Four Psychological Traits Variables, Respectively—Sensitivity Analysis Removing Cases of Uncertain Classification, Individuals With Possible EBV Infection at Inclusion or During the Observational Period, Individuals Vaccinated Before Baseline and Individuals Receiving Vaccination Less Than Five Days Prior to the Six Months Assessment **eTable 15.** Results of Final Factor Analyses (Principal Component Analysis) of Ten Clinical Symptoms Variables and Four Psychological Traits Variables, Respectively—Sensitivity Analysis Featuring Imputation of Mean/Median for Missing Data **eTable 16.** Characteristics of Potential Baseline Risk Factors and Their Univariate Associations (Poisson Regression With Log-Link and Robust Error Variances) to Long COVID-19 and Postinfective Fatigue Syndrome (PIFS) at Six Months Follow-up—Per Protocol Data **eTable 17.** Baseline Risk Factors and Their Univariate Associations (Poission Regression With Log-Link) to Long COVID-19 and Postinfective Fatigue Syndrome (PIFS) at Six Months Follow-up—Sensitivity Analysis Removing Cases of Uncertain Classification, Individuals With Possible EBV Infection at Inclusion or During the Observational Period, Individuals Vaccinated Before Baseline and Individuals Receiving Vaccination Less Than Five Days Prior to the Six Months Assessment **eTable 18.** Characteristics of Potential Baseline Risk Factors and Their Univariate Associations (Poisson Regression With Log-Link and Robust Error Variances) to Long COVID-19 and Postinfective Fatigue Syndrome (PIFS) at Six Months Follow-up—Sensitivity Analysis Featuring Imputation of Mean/Median Values for Missing Data **eTable 19.** Baseline Independent Risk Factors of Long COVID-19 and Postinfective Fatigue Syndrome (PIFS) at Six Months Follow-up—Final Multiple Regression Models (Modified Poisson Regression With Log-Link and Robust Error Variances)—Sensitivity Analysis Removing Cases of Uncertain Classification, Individuals With Possible EBV Infection at Inclusion or During the Observational Period, Individuals Vaccinated Before Baseline and Individuals Receiving Vaccination Less Than Five Days Prior to the Six Months Assessment **eTable 20.** Baseline Independent Risk Factors of Long COVID-19 and Postinfective Fatigue Syndrome (PIFS) at Six Months Follow-up—Final Multiple Regression Models (Modified Poisson Regression With Log-Link and Robust Error Variances)—Sensitivity Analysis Removing Cases of Uncertain Classification, Individuals With Possible EBV Infection at Inclusion or During the Observational Period, Individuals Vaccinated Before Baseline, Individuals Receiving Vaccination Less Than Five Days Prior to the Six Months Assessment, and Individuals in the SARS-CoV-2–negative Group With General Infectious Symptoms Score ≥11 at Baseline **eTable 21.** Baseline Independent Risk Factors of Long COVID-19 and Postinfective Fatigue Syndrome (PIFS) at Six Months Follow-up—Final Multiple Regression Models (Sensitivity Analysis Featuring Imputation of Mean/Median Values for Missing Data **eFigure 1.** Algorithm for Assessment of Long COVID-19 at Six Months Follow-up **eFigure 2.** Algorithm for Assessment of Postinfective Fatigue Syndrome (PIFS) at Six Months Follow-up **eFigure 3.** Spearman Rank Correlation Heatmap of Independent Variables
eReferences.

Supplement 2.
Data Sharing Statement
