# Supplementary material for: Prevalence and Characteristics Associated With Post–COVID-19 Condition Among Nonhospitalized Adolescents and Young Adults
Source: JAMA Netw Open. 2023 Mar 30;6(3):e235763. doi: 10.1001/jamanetworkopen.2023.5763 (PMC10064252; doi:10.1001/jamanetworkopen.2023.5763)
Supplement: Supplement 1. — eMethods. eTable 1. Results of All SARS-CoV-2 PCR Tests Performed Between December 24, 2020 and May 18, 2021 at Akershus University Hospital and Fürst Medical Laboratory, With Respect to Age and Sex eTable 2. Attritional Analyses: SARS-CoV-2–positive in Background Population, Proportions Invited to Participate, and Proportions Included in Study, With Respect to Age and Sex eTable 3. Attritional Analyses: Characteristics of Potential Baseline Risk Factors and Their Univariate Associations (Poisson Regression With Log-Link and Robust Error Variances) to Being Lost to Follow-up eTable 4. Analyses of Missing Data: Characteristics of Baseline Independent Variables and Their Association to Complete Cases at Six Months Follow-up eTable 5. Results of Epstein-Barr Virus (EBV) Serology at Baseline and Six Months Follow-up eTable 6. Epstein-Barr Virus (EBV) Infection Status of Individuals Attending Six Month Follow-up eTable 7. Point Prevalence Percentage of Long COVID-19 and Postinfective Fatigue Syndrome at Six Months Follow-up eTable 8. Point Prevalence (Confidence Intervals) of Long COVID-19 at Six Months Follow-up: Sensitivity Analysis Removing Cases of Uncertain Classification, Individuals With Possible EBV Infection at Inclusion or During the Observational Period, Individuals Vaccinated Before Baseline and Individuals Receiving Vaccination Less Than Five Days Prior to the Six Months Assessment eTable 9. Point Prevalence Percentage (Confidence Intervals) of Long COVID-19 at Six Months Follow-up: Sensitivity Analysis Removing Cases of Uncertain Classification, Individuals With Possible EBV Infection at Inclusion or During the Observational Period, Individuals Vaccinated Before Baseline, Individuals Receiving Vaccination Less Than Five Days Prior to the Six Month Assessment, and Individuals in the SARS-CoV-2 Negative Group With General Infectious Symptoms Score ≥11 at Baseline eTable 10. Point Prevalence Percentage (Confidence Intervals) of Postinfective Fatigue Syndrome ( [file jamanetwopen-e235763-s001.pdf]

Supplemental Online Content

Selvakumar J, Havdal LB, Drevvatne M, et al. Prevalence and characteristics associated with post–COVID-19 condition among nonhospitalized adolescents and young adults. *JAMA Netw Open.* 2023;6(3):e235763. doi:10.1001/jamanetworkopen.2023.5763

eMethods.

eTable 1. Results of All SARS-CoV-2 PCR Tests Performed Between December 24, 2020 and May 18, 2021 at Akershus University Hospital and Fürst Medical Laboratory, With Respect to Age and Sex

eTable 2. Attritional Analyses: SARS-CoV-2–positive in Background Population, Proportions Invited to Participate, and Proportions Included in Study, With Respect to Age and Sex

eTable 3. Attritional Analyses: Characteristics of Potential Baseline Risk Factors and Their Univariate Associations (Poisson Regression With Log-link and Robust Error Variances) to Being Lost to Follow-up

eTable 4. Analyses of Missing Data: Characteristics of Baseline Independent Variables and Their Association to Complete Cases at Six Months Follow-up

eTable 5. Results of Epstein-Barr Virus (EBV) Serology at Baseline and Six Months Follow-up

eTable 6. Epstein-Barr Virus (EBV) Infection Status of Individuals Attending Six Month Follow-up

eTable 7. Point Prevalence Percentage of Long COVID-19 and Postinfective Fatigue Syndrome at Six Months Follow-up

eTable 8. Point Prevalence (Confidence Intervals) of Long COVID-19 at Six Months Follow-up: Sensitivity Analysis Removing Cases of Uncertain Classification, Individuals With Possible EBV Infection at Inclusion or During the Observational Period, Individuals Vaccinated Before Baseline and Individuals Receiving Vaccination Less Than Five Days Prior to the Six Months Assessment

eTable 9. Point Prevalence Percentage (Confidence Intervals) of Long COVID-19 at Six Months Follow-up: Sensitivity Analysis Removing Cases of Uncertain Classification, Individuals With Possible EBV Infection at Inclusion or During the Observational Period, Individuals Vaccinated Before Baseline, Individuals Receiving Vaccination Less Than Five

© 2023 Selvakumar J et al. *JAMA Network Open.*

Days Prior to the Six Month Assessment, and Individuals in the SARS-CoV-2 Negative Group With General Infectious Symptoms Score ≥11 at Baseline

eTable 10. Point Prevalence Percentage (Confidence Intervals) of Postinfective Fatigue Syndrome (PIFS) at Six Months Follow-up: Sensitivity Analysis Removing Cases of Uncertain Classification, Individuals With Possible EBV Infection at Inclusion or During the Observational Period, Individuals Vaccinated Before Baseline and Individuals Receiving Vaccination Less Than Five Days Prior to the Six Months Assessment

eTable 11. Point Prevalence Percentage (Confidence Intervals) of Postinfective Fatigue Syndrome (PIFS) at Six Months Follow-up: Sensitivity Analysis Removing Cases of Uncertain Classification, Individuals With Possible EBV Infection at Inclusion or During the Observational Period, Individuals Vaccinated Before Baseline and Individuals Receiving Vaccination Less Than Five Days Prior to the Six Months Assessment, and Individuals in the SARS-CoV-2–negative Group With General Infectious Symptoms Score ≥11 at Baseline

eTable 12. Point Prevalence Percentage (Confidence Intervals) of Specific Symptoms at Baseline and Six Months Follow-up

eTable 13. Results of Final Factor Analyses (Principal Component Analysis) of Ten Clinical Symptoms Variables and Four Psychological Traits Variables, Respectively—Per Protocol Data

eTable 14. Results of Final Factor Analyses (Principal Component Analysis) of Ten Clinical Symptoms Variables and Four Psychological Traits Variables, Respectively—Sensitivity Analysis Removing Cases of Uncertain Classification, Individuals With Possible EBV Infection at Inclusion or During the Observational Period, Individuals Vaccinated Before Baseline and Individuals Receiving Vaccination Less Than Five Days Prior to the Six Months Assessment

eTable 15. Results of Final Factor Analyses (Principal Component Analysis) of Ten Clinical Symptoms Variables and Four Psychological Traits Variables, Respectively—Sensitivity Analysis Featuring Imputation of Mean/Median for Missing Data

eTable 16. Characteristics of Potential Baseline Risk Factors and Their Univariate Associations (Poisson Regression With Log-Link and Robust Error Variances) to Long COVID-19 and Postinfective Fatigue Syndrome (PIFS) at Six Months Follow-up—Per Protocol Data

eTable 17. Baseline Risk Factors and Their Univariate Associations (Poission Regression With Log-Link) to Long COVID-19 and Postinfective Fatigue Syndrome (PIFS) at Six Months Follow-up—Sensitivity Analysis Removing Cases of Uncertain Classification, Individuals With Possible EBV Infection at Inclusion or During the Observational Period, Individuals Vaccinated Before Baseline and Individuals Receiving Vaccination Less Than Five Days Prior to the Six Months Assessment

eTable 18. Characteristics of Potential Baseline Risk Factors and Their Univariate Associations (Poisson Regression With Log-Link and Robust Error Variances) to Long

© 2023 Selvakumar J et al. *JAMA Network Open.*

COVID-19 and Postinfective Fatigue Syndrome (PIFS) at Six Months Follow-up—Sensitivity Analysis Featuring Imputation of Mean/Median Values for Missing Data

eTable 19. Baseline Independent Risk Factors of Long COVID-19 and Postinfective Fatigue Syndrome (PIFS) at Six Months Follow-up—Final Multiple Regression Models (Modified Poisson Regression With Log-Link and Robust Error Variances)—Sensitivity Analysis Removing Cases of Uncertain Classification, Individuals With Possible EBV Infection at Inclusion or During the Observational Period, Individuals Vaccinated Before Baseline and Individuals Receiving Vaccination Less Than Five Days Prior to the Six Months Assessment

eTable 20. Baseline Independent Risk Factors of Long COVID-19 and Postinfective Fatigue Syndrome (PIFS) at Six Months Follow-up—Final Multiple Regression Models (Modified Poisson Regression With Log-Link and Robust Error Variances)—Sensitivity Analysis Removing Cases of Uncertain Classification, Individuals With Possible EBV Infection at Inclusion or During the Observational Period, Individuals Vaccinated Before Baseline, Individuals Receiving Vaccination Less Than Five Days Prior to the Six Months Assessment, and Individuals in the SARS-CoV-2–negative Group With General Infectious Symptoms Score ≥11 at Baseline

eTable 21. Baseline Independent Risk Factors of Long COVID-19 and Postinfective Fatigue Syndrome (PIFS) at Six Months Follow-up—Final Multiple Regression Models (Sensitivity Analysis Featuring Imputation of Mean/Median Values for Missing Data

eFigure 1. Algorithm for Assessment of Long COVID-19 at Six Months Follow-up

eFigure 2. Algorithm for Assessment of Postinfective Fatigue Syndrome (PIFS) at Six Months Follow-up

eFigure 3. Spearman Rank Correlation Heatmap of Independent Variables

eReferences.

This supplemental material has been provided by the authors to give readers additional information about their work.

eMethods.

1.1. Design of the LoTECA project

Overall design

The project entitled Long-Term Effects of COVID-19 in Adolescents (LoTECA) is a prospective cohort study investigating the long-term consequences of acute infection with Severe Acute Respiratory Syndrome Coronavirus 2 (SARS-CoV-2) in non-hospitalised adolescents and young adults aged 12 to 25 years (ClinicalTrials ID: NCT04686734).[1] The project enrolled a total of 404 individuals with a positive Polymerase Chain Reaction (PCR) test for SARS-CoV-2 (SARS-CoV-2-positive group), as well as 105 individuals with a negative PCR test (SARS-CoV-2-negative group) for baseline investigations (cf. flowchart below). After six months, a follow-up investigation was carried out in all participants. Participants who met criteria for fatigue caseness at six months (Chalder Fatigue Questionnaire total sum score ≥ 4, bimodal (0-0-1-1) scoring of single items, cf. paragraph 1.6 below) were recalled for a second follow-up investigation at 12 months. The present paper reports data from baseline and six months follow-up only. The 12 months follow-up appointments was completed June 2022. At each time point, participants completed a standardised assessment program at our study center lasting about 1.5 hours, and encompassing: a) clinical interview and examination; b) functional testing; c) sampling of biological material; and d) completion of a questionnaire. Further details are provided in the sections below.

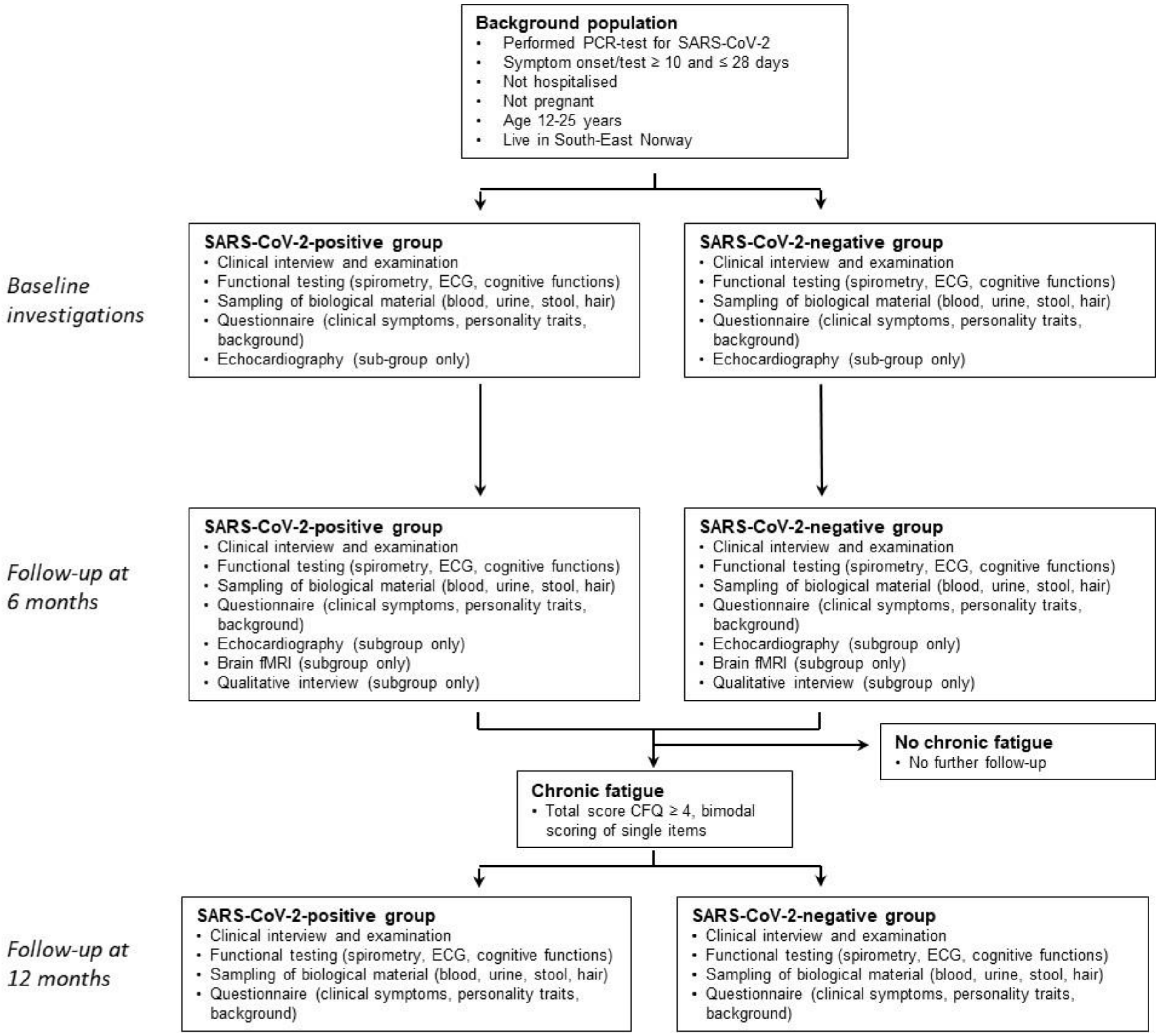

Study flowchart of the entire LoTECA project. Follow-up at 12 months was completed June 2022.

© 2023 Selvakumar J et al. *JAMA Network Open.*

LoTECA sub-studies

In addition, subgroups of participants were included in three different LoTECA sub-studies, results of which are not reported in the present paper:

- A brain imaging sub-study, using functional magnetic resonance imaging (fMRI) to identify aberrations in sufferers of long COVID.
- An echocardiography sub-study, to identify associations between patients' symptoms and disability and indices of circulatory function (including echocardiographic markers, blood biochemical markers and autonomic function tests).
- A qualitative sub-study, to obtain a richer description of long COVID among adolescents, primarily addressing coping, coping beliefs and hope.

1.2. Recruitment, inclusion and exclusion criteria

Recruitment

From December 24, 2020 until May 18, 2021, study participants were recruited from two accredited microbiological laboratories (Fürst Medical Laboratory; Dept. of Microbiology and Infection Control, Akershus University Hospital). These two laboratories provided comprehensive microbiological testing services (upper respiratory tract swabs followed by reverse-transcription polymerase chain reaction (RT-PCR)) to the population of Oslo and Viken counties, Norway, during the entire COVID-19 pandemic. Testing for SARS-CoV-2 was undertaken for those with symptoms of an acute COVID-19, such as fever, sore throat, cough or loss of taste and smell, or recent exposure to someone with a confirmed case of SARS-CoV-2 infection. Test results from individuals 12-25 years old were continuously reported to the LoTECA study centre. Eligible individuals were first contacted by a Short Text Message explaining the purpose of the study and asking for permission to receive a phone call. A subsequent phone conversation clarified inclusion and exclusion criteria, and provided contact information for forwarding the written project information and consent form. For potential participants under 16 years of age, their parents/next-of-kin were also informed. If consent was given, the first (baseline) appointment at the LoTECA study centre was scheduled at least 10 days after the first onset of symptoms (quarantine period), but no more than 28 days. Additionally, participants were not allowed to have had fever for at least 24 hours prior to the baseline investigations. Travel expenses to the LoTECA study center were covered, and all participants also received a gift card of 400 NOK as compensation for their contribution.

Inclusion and exclusion criteria

Inclusion and exclusion criteria are shown below. Study participants were restricted to patients living in the counties of Oslo and Viken (in the South-East region of Norway) for practical reasons and to secure adherence to the follow-up through proximity to the LoTECA study center.

Criteria for inclusion and exclusion

SARS-CoV-2-positive group	SARS-CoV-2-negative group
INCLUSION CRITERIA	
Suspected SARS-CoV-2 infection (symptoms or exposure)	Suspected SARS-CoV-2 infection (symptoms or exposure)
Positive SARS-CoV-2 PCR test	Negative SARS-CoV-2 PCR test
Age 12-25 years	Age 12-25 years
≤ 28 days since onset of first symptom	≤ 28 days since onset of first symptom
EXCLUSION CRITERIA	
Hospitalised because of COVID-19	Antibodies suggesting previous COVID-19[a]
Pregnancy	Pregnancy
Lack of consent from patient/next-of-kin	Lack of consent from patient/next-of-kin

[a] Elevated total anti-nucleocapsid IgM and IgG and/or elevated anti-receptor binding domain IgG for unvaccinated individuals.

1.3. Clinical interview and examination

Prior to each appointment at the LoTECA study centre, participants were instructed to abstain from tobacco products, caffeine and over-the-counter pharmaceuticals (such as paracetamol and ibuprofen) for at least 48 hours. They were also offered local anaesthetic ointment (EMLA®, AstraZeneca) to apply on the antecubital areas one hour before arriving, to avoid the pain of venous puncture. Finally, they were instructed to bring a morning spot urine sample in a sterile container as well as a stool sample using manufactured sampling devices (Bio-Me, Oslo, Norway).

The clinical interview included questions on country of birth of participants and their parents, as well as the history of medical and psychiatric disorders and current regular use of pharmaceuticals. The physical examination encompassed a structured

© 2023 Selvakumar J et al. *JAMA Network Open.*

review of all organ systems, particularly focusing on respiratory (stridor, wheezing, retractions, crackling sounds), cardiovascular (murmur) and neurological (focal signs) abnormalities. Weight and height was measured with SECA® 877 scale and SECA® height rod 0123 (SECA, Birmingham, United Kingdom). Blood pressure were obtained using Connex® ProBP™ 3400 Non-invasive Blood Pressure Device (Welch Allyn, NY, USA). Blood oxygen saturation (SpO2) was measured with Nellcor™ Portable SpO2 Patient monitoring system, PN10M (Covidien, Medtronic, MN, USA). Tympanic temperature was recorded using ThermoScan® PRO 6000 (Welch Allyn, Macquarie Park, Australia). The urine sample was assayed with a Multistix 5 (Siemens Healthcare, Erlangen, Germany); also, a pregnancy test was performed if indicated, applying Alere™ hCG Cassette (Abon biopharm, Hangzhou, P.R. China).

Body Mass Index was normalised to World Health Organization 2006 Child Growth Standards, which provides z-scores for ages 12 to 19.[2] For participants above this age, the reference values for 19-year-olds were used.

1.4. Functional testing

Spirometry

Spirometry was conducted to measure the forced vital capacity (FVC) and the forced expiratory volume in one second (FEV_1) (EasyOne® Air spirometer, EasyOne Connect software, NDD Medizintechnic AG, Switzerland). The ratio of FEV_1/FVC was calculated. Procedures were executed according to the American Thoracic Society and European Respiratory Society guidelines, and recordings that did not adhere to technical quality requirements were excluded from the main analysis.[3] The Global Lung Function Initiative 2012 network reference values were used to calculate the percentage of predicted values and the lower limit of normal (LLN).[4]

ECG recording and autonomic cardiovascular control

A 5-minute ECG recording was performed applying The Bittium Faro 360® device (Bittium Corporation, Oulu, Finland). During recording, participants were laying supine in a dark room with calm surroundings. Recordings were analysed using manufacturer developed software, providing automatic R-wave detection and exclusion of arrhythmias (including ectopic beats). Heart rate variability (HRV) indices were calculated in the time domain, as well as in the frequency domain after Fast Fourier Transformation of the time series, according to international standards.[5] Computed time domain indices include SDNN (the standard deviation of all RR-intervals), pNN50 (the proportion of successive RRIs with a difference greater than 50 ms), and r-MSSD (the square root of the mean square differences of successive RRIs). In the frequency domain, power densities were computed in the low-frequency (LF) band (0.04-0.15 Hz) and the high-frequency (HF) band (0.15-0.5 Hz), and expressed both in absolute ($LF_{abs,}$ HF_{abs}) and normalized units, where $LF_{norm}= LF_{abs} /(LF_{abs} + HF_{abs})$ and $HF_{norm}= HF_{abs} /(LF_{abs} + HF_{abs})$. In addition, the LF_{abs}/HF_{abs} ratio was computed. Vagal (parasympathetic) activity is considered the main contributor to HF-variability of heart rate, whereas both vagal and sympathetic activity contributes to LF-variability. In 57 normal subjects (age 20-60 years), we analyzed the spontaneous beat-to-beat oscillation in R-R interval during control recumbent position, 90 degrees upright tilt, controlled respiration (n = 16) and acute (n = 10) and chronic (n = 12) beta-adrenergic receptor blockade. Automatic computer analysis provided the autoregressive power spectral density, as well as the number and relative power of the individual components. The power spectral density of R-R interval variability contained two major components in power, a high frequency at approximately 0.25 Hz and a low frequency at approximately 0.1 Hz, with a normalized low frequency:high frequency ratio of 3.6 +/- 0.7. With tilt, the low-frequency component became largely predominant (90 +/- 1%) with a low frequency:high frequency ratio of 21 +/- 4. Acute beta-adrenergic receptor blockade (0.2 mg/kg IV propranolol) increased variance at rest and markedly blunted the increase in low frequency and low frequency:high frequency ratio induced by tilt. Chronic beta-adrenergic receptor blockade (0.6 mg/kg p.o. propranolol, t.i.d.), in addition, reduced low frequency and increased high frequency at rest, while limiting the low frequency:high frequency ratio increase produced by tilt. Controlled respiration produced at rest a marked increase in the high-frequency component, with a reduction of the low-frequency component and of the low frequency:high frequency ratio (0.7 +/- 0.1); during tilt, the increase in the low frequency:high frequency ratio (8.3 +/- 1.6) was significantly smaller. In seven additional subjects in whom direct high-fidelity arterial pressure was recorded, simultaneous R-R interval and arterial pressure variabilities were examined at rest and during tilt. Also, the power spectral density of arterial pressure variability contained two major components, with a relative low frequency:high frequency ratio at rest of 2.8 +/- 0.7, which became 17 +/- 5 with tilt. These power spectral density components were numerically similar to those observed in R-R variability. Thus, invasive and noninvasive studies provided similar results. More direct information on the role of cardiac sympathetic nerves on R-R and arterial pressure variabilities was derived from a group of experiments in conscious dogs before and after bilateral stellectomy. Under control conditions, high frequency was predominant and low frequency was very small or absent, owing to a predominant vagal tone. During a 9% decrease in arterial pressure obtained with IV nitroglycerin, there was a marked increase in low frequency, as a result of reflex sympathetic activation.[6,7] The LF/HF ratio is considered an index of sympathovagal balance.

Cognitive function tests

All cognitive function tests were carried out by trained examiners in a separate room with calm surroundings. The Digit Span Test was adopted from the Wechsler Intelligence Scale for Children, 4th edition (WISC-IV).[8] This test is used for verbal and auditory working memory assessment. A string of random digits was read aloud by the examiner. The first string consists of two random numbers, and for every other string, one more number is added. The digit span forward mode required the test subject to repeat the digits in the same order as they were presented; in the digit span backward mode, digits were repeated in reverse order. Each correctly repeated string was scored one point. The test was discontinued when two strings of equal length were answered incorrectly. Sum scores for digit span forward and backward, as well as total sum score, were computed.

A test of verbal learning, delayed recall, and recognition was adopted from the Hopkins Verbal Learning Test-Revised (HVLT-R).[9] The examiner read aloud a list of 12 words and the participant was asked to repeat as many words as possible in three consecutive trials. An index of verbal learning memory was computed as the sum score of remembered words (ranging from 0 to 36) across the three trials. After 20 minutes, the examiner asked the participants to report as many words as possible; an index of delayed verbal memory was computed as the number of words the test subject were able to recall correctly (ranging from 0 to 12). Finally, a total of 24 words were read aloud by the examiner, of which 12 were identical to the previous list of words; the number of correctly recognized and falsely recognized words was recorded separately (both indices ranging from 0 to 12).

A computerised test of attention bias towards illness-related words were implemented as described by Hughes and co-workers.[10] This test measured reaction times to illness-related words and neutral word pairs; faster reaction times to probes replacing (appearing in the location of) illness-related words relative to probes replacing neutral words indicate an attentional bias. Results from the attention bias test is not reported in the present paper.

The computerised Function Acquisition Speed Test (FAST) of cognitive fusion (ie., to what extent behaviour is overly regulated by thoughts and perceptions rather than external contextual clues) was implemented as described by O'Reilly and co-workers.[11] The FAST assesses the differential rate at which relations between classes of words are acquired in two differing training configurations. Results are not reported in the present paper.

1.5. Sampling of biological specimens and laboratory assays

Sampling and biorepository procedures

The primary biological specimens obtained were blood, hair, urine, and stool. Blood samples were obtained from antecubital venous puncture. If requested by the participants, local anaesthetic ointment (EMLA®) was applied for at least 60 minutes, but removed 15 minutes prior to sampling, cf. paragraph 1.3 above. A hair sample was collected from the parietal/occipital region of the scalp, where a bundle of hair with approximately the same diameter as a pencil was cut as close to the scalp as possible. Urine and stool samples were collected by the participants themselves, in the morning on the day of investigation, cf. paragraph 1.3 above.

Blood samples for routine analysis were immediately delivered to the accredited laboratory at Akershus University Hospital, Norway. Blood samples for storage at the biorepository underwent further preparations in order to obtain aliquots of plasma, serum, whole blood, RNA and viable Peripheral Blood Mononuclear Cells (PBMC). Thereafter, blood derived material not subjected to further analyses as well as the hair, urine and stool samples were transferred to a biorepository adjacent to the study centre (EpiGen laboratories, Akershus University Hospital, Norway), and stored at –80 ºC or –150 ºC, as appropriate. Results from analyses of whole blood, RNA, PBMC, hair, urine and stool are not reported in the present paper.

Cytokines, growth factors and complement activation markers

EDTA whole blood samples were placed on ice-water for 5-60 minutes. Thereafter, plasma was separated by centrifugation (2200 g, 10 min.) and frozen at –80 °C until assayed. Plasma samples were analyzed using a multiplex cytokine assay (Bio-Plex Human Cytokine 27-Plex Panel; Bio-Rad Laboratories Inc., Hercules, CA, USA) containing the following cytokines: IL-1β, IL-1 receptor antagonist (IL1-ra), IL-2, IL-4, IL-5, IL-6, IL-7, IL-8, IL-9, IL-10, IL-12, IL-13, IL-15, IL-17A, eotaxin, basic fibroblast growth factor (bFGF), granulocyte-colony stimulating factor (G-CSF), granulocyte macrophage colony stimulating factor (GM-CSF), interferon (IFN)-γ, interferon-inducible protein (IP-10), monocyte chemotactic protein (MCP-1), macrophage inflammatory protein (MIP)-1α, MIP-1β, platelet derived growth factor-BB (PDGF-BB), regulated upon activation T cell expressed and secreted (RANTES), Tumor Necrosis Factor (TNF), and vascular endothelial growth factor (VEGF). The samples were analyzed on a Multiplex Analyser (Bio-Rad Laboratories) according to instructions from the manufacturer.

Plasma levels of growth/differentiation factor (GDF)-15 and C-reactive protein (CRP) were measured in duplicate by enzyme immunoassays (EIA) using commercially available antibodies (R&D Systems, Minneapolis, MN, USA) in a 384-format using

a combination of a SELMA (Jena, Germany) pipetting robot and a BioTek (Winooski, VT) dispenser/washer. Absorption was read at 450 nm with wavelength correction set to 540 nm using an ELISA plate reader (BioTek, Winooski, VT).

The complement activation products C3bc and the terminal complement complex (TCC) sC5b-9 were quantified in plasma using enzyme-linked immunosorbent assays (ELISAs) based on monoclonal antibodies designed against neoepitopes of the products, not reacting with the native component.[12] The units of these two well-established in-house assays are given according to an international standard defined as complement activation units (CAU) per milliliter with blood donors to define upper reference values of the normal population.

SARS-CoV-2-antibodies

Serum samples were tested with the Elecsys® Anti-SARS-CoV-2 immunoassay (Roche Diagnostics, Cobas e801, Mannheim, Germany) for IgG/IgM against the SARS-CoV-2 nucleocapsid antigen. The specificity and the sensitivity of the test are estimated by the manufacturer as 99.8% and 99.5%, respectively. In addition, antibodies to full-length spike protein (Spike-FL) and the receptor-binding domain (RBD) were measured using a multiplexed bead-based assay described in detail earlier.[13] Briefly, sera were diluted 1:100 and incubated for 30 min with polymer beads with fluorescent bar codes coupled to Spike-FL or RBD. The beads were next washed, and aliquots were labelled with R-Phycoerythrin-conjugated anti-human IgG Fc (Jackson ImmunoResearch, West Grove, PA) and analyzed by flow cytometry (Attune Next, Thermo Fisher Scientific, Waltham, MA). The median fluorescence intensity (MFI) of beads coupled with viral antigens was divided by the MFI measured for beads with no antigen. Effects of sera on ACE2-binding to RBD were measured as a proxy for neutralizing antibodies. The beads were incubated with sera as described above, but labelled with digoxigenin-conjugated ACE2 and R-Phycoerythrin-conjugated anti-digoxigenin (Jackson ImmunoResearch, West Grove, PA). Signals measured in sera with no detectable anti-RBD were used as reference for no inhibitory effect.

Epstein-Barr virus antibodies

Specific antibody responses were assessed in serum samples using EBV VCA IgM and IgG (LIAISON®, DiaSorin, Saluggia, Italy) and EBV EBNA IgG (LIAISON®, DiaSorin, Saluggia, Italy). A rapid chromatographic immunoassay for the qualitative detection of Infectious Mononucleosis heterophile antibodies, Clearview® IM II (Abbott Laboratories, USA), was performed on serum samples with inconclusive result from the three specific tests. The specificity and the sensitivity of EBV-VCA IgM are estimated by the manufacturer as 99.2 % and 97.8 %, respectively; for EBV-VCA IgG 95.8 % and 98.5 %, respectively; and for EBV-EBNA IgG 97.6 % and 98.8 %, respectively. The manufacturer states >99 % negative and positive agreement between Clearview® IM II and slide agglutination.

Brain injury markers

Blood for neurofilament light chain (NfL) and glial fibrillary acidic protein (GFAp) measurements in serum was collected in 3,5 mL Vacuette R (Greiner Bio-One GmbH, Kremsmünster, Austria) with gel, allowed to clot for at least 30 minutes, processed within 2 hours by centrifugation (2200 g, 10 min) and aliquots stored immediately at − 80 °C until analysis. Serum GFAp and NfL measurements were performed at the Clinical Neurochemistry Laboratory, Sahlgrenska University Hospital, Sweden, by board-certified laboratory technicians blinded to clinical data using commercially available Single molecule array (Simoa) assays on an HD-X Analyzer (Human Neuro 2-Plex B assay), as described by the manufacturer (Quanterix, Billerica, MA). Calibrators were run in duplicates, while samples were diluted 4-fold and run in singlicates. Two quality control (QC) samples with different levels were run in duplicates in the beginning and the end of each run. Repeatability and intermediate precision were both 8.7% for the QC sample with an NfL concentration of 8.4 pg/mL and 5.9% for the 79.6 pg/mL sample. For GFAP, repeatability was 6.5% and intermediate precision 7.3% for the QC sample at 102 pg/mL, and repeatability was 5.8% and intermediate precision 6.7% for the QC sample at 388 pg/mL.

Routine blood analyses

Routine blood analyses were assayed at the accredited laboratory at Akershus University Hospital, Norway, and included the following markers: haemoglobin; leukocytes with differential count; platelets; CRP; ferritin; alanine transaminase (ALT); gamma-glutamyltransferase (GGT); lactate dehydrogenase (LDH); albumin; N-terminal prohormone of Brain Natriuretic Peptide (NT-proBNP); troponin T; creatine kinase (CK); glucose; glycated haemoglobin (HbA_{1C}); bilirubin; D-dimer; international normalized ratio (INR); urea; creatinine; natrium; potassium; calcium; vitamin B_{12}; folic acid; thyroid-stimulating hormone (TSH); thyroxine; cortisol; IgG (total); IgM (total); IgA (total); blood gases (venous sample); SARS-CoV-2 total antibody titer (IgM+IgG).

1.6. Questionnaires

A composite questionnaire consisting of validated inventories were used to chart clinical symptoms, personality traits, and social factors as well as basic demographic and constitutional variables. An overview is provided below. Responses were used in the regression analyses as well as for categorization according to the WHO case-definition of long COVID,[14] and the

© 2023 Selvakumar J et al. *JAMA Network Open.*

international diagnostic criteria for PIFS,[15] cf. paragraphs 1.7 and 1.8 below. The questionnaire was administered digitally using the "Nettskjema"-tool administered by the Services for Sensitive Data at the University of Oslo.[16] This tool ascertains that all items are completed before submission to a dedicated and secured server area where scores are automatically computed following a predefined scoring algorithm. All participants answered the questionnaire using a designated computer at our study center as part of the investigational program at baseline and follow-up, cf. paragraph 1.3 above.

Composite questionnaire overview: Constructs, inventories and scoring procedures

Construct(s)	Name of inventory	Description and scoring procedures
**BACKGROUND AND DEMOGRAPHICS**		
Household, socioeconomic level	Not applicable	Household members; parents' occupation; the international socio-economic index (ISEI) of occupational status were used to score socio-economic level.[17,18]
Smoking, alcohol, drugs	Not applicable	Alcoholic beverages, illicit drugs, smoking; answered on a 5-point Likert scale, where 1 is "never" and 5 is "every day/almost every day".
Physical activity	Not applicable	Answered on a 5-point Likert scale, where 1 is "a lot less active than peers" and 5 is "a lot more active than peers".
Diseases	Not applicable	Comorbidity; chronic disease affecting parents or siblings; undergone acute COVID-19 (only asked at follow-up)
Vaccines	Not applicable	Received vaccination against COVID-19 (number of dosages, manufacturer; only asked at follow-up)
**SYMPTOMS AND DISABILITY**		
Fatigue	Chalder Fatigue Questionnaire (CFQ)	A total of 11 items scored on 4-point Likert scales. In order to obtain a continuous variable, each item was scored 0-3 where 0 is "less than usual" and 3 is "much more than usual"; then, a total sum score across all items was obtained ranging from 0 to 33, where higher scores indicate more fatigue.[19] In addition, bimodal scoring (0-0-1-1) of each item was performed; a total sum score across all items of 4 or higher was defined as fatigue caseness.
Clinical symptoms of long COVID and PIFS	CDC symptom inventory for Chronic Fatigue Syndrome	A total of 30 items addressed frequency of specific symptoms since falling ill from acute COVID-19 on 5-point Likert scales, where 1 is "never" and 5 is "all the time". [20]At follow-up, the questions were slightly rephrased in order to address symptom frequency during the last months. Follow-up answers were used to define caseness of long COVID and PIFS, cf. paragraph 1.7 below.
Post-exertional malaise (PEM)	PEM items from the DePaul Symptom Questionnaire	A total of five items addressed frequency of PEM symptoms on 5-point Likert scales, where 0 is "never" and 4 is "all the time"; answers where then averaged across all items and multiplied with 25 to get a 100 point scoring scale where higher scores indicate more PEM.[21,22]
Sleep disturbances	Karolinska Sleep Questionnaire (KSQ)	A total of 12 items addressed frequency of sleep disturbances on 6-point Likert scales, where 1 is "never" and 6 is "all the time"; then, the scoring were reversed, and total sum score was computed across all items ranging from 12 to 72, where *lower* scores indicate more sleep disturbances.[23] Accordingly, indexes for insomnia, awakening problems, and sleepiness were computed as sum scores across relevant items.
Pain	Brief Pain Inventory (BPI)	A total of four items addressed different aspects of pain on 10-point Likert scales, where 1 is "no pain" and 10 is "worst pain imaginable"; total sum score was computed across all items ranging from 4 to 40, where higher scores indicate more pain.[24]
Depression and anxiety symptoms	Hospital Anxiety and Depression Symptoms (HADS)	A total of 14 items addressed different symptoms of depression and anxiety on 4-point Likert scales scored 0 – 3; for eight of the items, scoring were reversed, after which total sum score was computed ranging from 0 to 42, where higher scores indicate more symptoms of depression and anxiety.[25] Accordingly, separate indexes for depression and anxiety were computed as sum scores across relevant items (seven each).
Negative affect	Positive and Negative Affect Schedule, short-form (PANAS-SF)	A total of five items addressing negative affects (shameful, anxious, nervous, hostile, offended) on 5-point Likert scales, where 1 is "disagree completely" and 5 is "agree completely"; total sum score was computed ranging from 5-25, where higher scores indicate more negative affects.[26]
Illness perception	Brief Illness Perception Questionnaire (BPIQ)	A total of eight items addressing perceived impact of acute COVID-19 were scored on 10-point Likert scale scored 1 – 10; total sum score was computed ranging from 8 to 80, where higher scores indicate more perceived impact.[27] Due to a mistake in the questionnaire design process, we did not apply the original scoring procedure as proposed by Broadbent et al., which is based on 11-point Likert scales. At follow-up, the questions were slightly rephrased in order to address 'symptoms following COVID-19'.
Quality of life	Pediatric Quality of Life (PedsQL)	A total of 23 items addressing different aspects of quality of life (QoL) were scored on 5-point Likert scales where 0 is "never" and 4 is "almost always"; scores were multiplied with 25 to get a 100 point scale and then averaged across all items, implying that higher scores indicate better QoL.[28,29] In addition, separate indexes for four QoL subdomains (health related, emotional, social, school) were computed as average scores across relevant items. In order to fit the age span of the participants in the present study, a few items were slightly rephrased; for instance, "school" was substituted with "school/work".
Interoceptive attention	Body Vigilance Scale (BVS)	A total of four items addressing interoceptive phenomena were scored on 11-point Likert scales.[30] One of the items asks the respondent to indicate percent of time (from 0 to 100)

© 2023 Selvakumar J et al. *JAMA Network Open.*

		spent on monitoring internal bodily states; scores were divided by 10 to obtain a 0–10 point scoring scale. Another item asks the respondent to score the amount of attention directed towards a total of 14 different bodily sensations; answers were averaged across all these sensations. Finally, a total sum score across the four items were computed ranging from 0 to 40, where higher scores imply more interoceptive attention
Miscellaneous	Not applicable	• One item addressed avoidance behavior on a 10-point Likert scale, where higher scores indicate more avoidance tendency.<br>• One item addressed school/work absenteeism as number of totally absent days during the last month.
**PSYCHOLOGICAL TRAITS AND SOCIAL FACTORS**		
Neuroticism	NEO Five-Factor Inventory-30 (NEO-FFI-30)	A total of six items making up the neuroticism axis were included and scored on 5-point Likert scales where 0 is “disagree completely” and 4 is “agree completely”; total sum score across all items were computed ranging from 0 to 24, where higher scores indicate stronger neuroticism tendencies.[31]
Worrying tendencies	Penn State Worry Questionnaire (PSWQ)	A total of 16 items addressing worrying tendencies were scored on 5-point Likert scales where 1 is “disagree completely” and 5 is “agree completely”; scoring were reversed on five items, after which the total sum score across all items was computed ranging from 16 to 80, where higher scores indicate stronger worrying tendencies.[32]
Emotional awareness	Toronto Alexithymia Scale (TAS-20)	A total of seven items making up the index of Difficult identifying feelings were included and scored on 5-point Likert scales where 1 is “disagree completely” and 5 is “agree completely”; total sum score was computed across all items ranging from 7 to 49, where higher scores indicate poorer emotional awareness (ie. more difficulties identifying feelings).[33]
Loneliness	UCLA Loneliness Scale	A total of 20 items addressing loneliness were scored on 4-point Likert scales where 1 is “never” and 4 is “always”; scorings were reversed on nine items, after which the total sum score was computed ranging from 20 to 80, where higher scores indicate more loneliness.[34]
Self-efficacy	General Self-Efficacy Scale, short form (GSE-6)	A total of six items addressing self-efficacy was scored on 4-point Likert scales where 1 is “disagree completely” and 4 is “agree completely”; total sum across all items was computed ranging from 6 to 24, where higher scores indicate better self-efficacy.[35]
Life events	Life Events Checklist (LEC)	A total of 48 prespecified life events were presented; for each of them, and the respondents were expected to indicate whether they had encountered the specific event during the last year, and if so, whether they considered the event to be good or bad and assess its subjective impact on a 4-point Likert scale where 0 is “no impact” and 3 is “large impact”. Also, the respondents were allowed to list additional events. Finally, an identical procedure was undertaken for events having occurred any time in the past. Number of positive and negative life events were computed separately for ‘last year’ and ‘any time in the past’; accordingly, sum scores for subjective impact were computed.
Miscellaneous	Child-Adolescent Perfectionism Scale (CAPS); Highly Sensitive Person Scale (HSP); Parenting Dimension Inventory (PDI).	• One item (“Others always expect me to be perfect”) was picked from the CAPS inventory in order to address socially prescribed perfectionism; the item is scored on a 5-point Likert scale where 1 is “disagree completely” and 5 is “agree completely”.[36]<br>• Two items were included from the HSP: Startling tendencies and tendencies to be affected by other people’s emotions.[35] Both items were scored on 5-point Likert scales where 1 is “disagree completely” and 5 is “agree completely”.<br>• Two items were included from the PDI, both addressing parental control.[37] They were scored on 4-point Likert scales where 1 is “disagree completely” and 5 is “agree completely”.<br>• A total of four self-invented items addressing interoceptive awareness and positive expectancies were included; all were scored on 5-point Likert scales where 1 is “disagree completely” and 5 is “agree completely.

1.7. Caseness assessment

The wording of the WHO diagnostic definition of post-COVID-19 condition (the term used by the WHO for long COVID)[14] as well as the international criteria for the diagnosis of PIFS[15] were scrutinized in order to establish operationalised definitions based upon available data in the LoTECA project. As a general approach, questionnaire data on clinical symptoms and functional disability were used to define potential cases, whereas other questionnaire data as well as clinical and laboratory findings were used to identify possible exclusionary criteria. Potential cases *without* possible exclusionary criteria were classified as ‘certain cases’, while for cases *with* possible exclusionary criteria were further scrutinized by two researchers independently and blinded for initial SARS-CoV-2 status, and eventually labelled “uncertain cases” if classification remained uncertain. The processes are outlined in detail below and in Figures S1 and S2.

For the WHO case definition of long COVID, a list of 13 clinical symptoms found to be persistently prevalent among COVID-19 sufferers in a large population-based Norwegian study guided the selection of questionnaire items used to screen for potential caseness.[37] Individuals reporting at least one of these symptoms 1-2 times a week or more were considered to fulfil the persistent symptom requirement of the WHO definition of long COVID.

© 2023 Selvakumar J et al. *JAMA Network Open.*

Operationalisation of the WHO case definition of long COVID[a,14]

Variable	Criterion	Comment
**1. Persistent symptoms (cases must adhere to at least one)**		
a) "… experienced altered smell and/or taste."	≥ 3	Single item (5-point Likert scale, 3 indicates 1-2 times a week) from the CDC symptom inventory for Chronic Fatigue Syndrome.[20]
b) "… experienced shortness of breath/dyspnea."	≥ 3	Single item (5-point Likert scale, 3 indicates 1-2 times a week) from the CDC symptom inventory for Chronic Fatigue Syndrome.[20]
c) "… experienced chest pain."	≥ 3	Single item (5-point Likert scale, 3 indicates 1-2 times a week) from the CDC symptom inventory for Chronic Fatigue Syndrome.[20]
d) "… experienced memory problems."	≥ 3	Single item (5-point Likert scale, 3 indicates 1-2 times a week) from the CDC symptom inventory for Chronic Fatigue Syndrome.[20]
e) Fatigue score.	≥ 4	Total score on the Chalder Fatigue Questionnaire, bimodal scoring (0-0-1-1) of single item.[19] This definition of fatigue caseness has been applied in several previous publications.[38–41]
f) PEM score.	≥ 2 for at least 1 of 5 items	From the DePaul symptom questionnaire: Five items addresses frequency of PEM symptoms on 5-point Likert scales, where 0 is "never" and 4 is "all the time".[21,22]
g) "….experienced palpitations."	≥ 3	Single item (5-point Likert scale, 3 indicates 1-2 times a week) from the CDC symptom inventory for Chronic Fatigue Syndrome.[20]
h) "… experienced concentration problems."	≥ 3	Single item (5-point Likert scale, 3 indicates 1-2 times a week) from the CDC symptom inventory for Chronic Fatigue Syndrome.[20]
i) "… experienced problems making decisions."	≥ 3	Single item (5-point Likert scale, 3 indicates 1-2 times a week) from the CDC symptom inventory for Chronic Fatigue Syndrome.[20]
j) "….experienced feeling of fever/chills."	≥ 3	Single item (5-point Likert scale, 3 indicates 1-2 times a week) from the CDC symptom inventory for Chronic Fatigue Syndrome.[20]
k) "… experienced cough."	≥ 3	Single item (5-point Likert scale, 3 indicates 1-2 times a week) from the CDC symptom inventory for Chronic Fatigue Syndrome.[20]
l) "… experienced dizziness."	≥ 3	Single item (5-point Likert scale, 3 indicates 1-2 times a week) from the CDC symptom inventory for Chronic Fatigue Syndrome.[20]
m) "… experienced headache."	≥ 3	Single item (5-point Likert scale, 3 indicates 1-2 times a week) from the CDC symptom inventory for Chronic Fatigue Syndrome.[20]
**2. Functional disability (cases must adhere to the criterion)**		
a) PedsQL (Pediatric Quality of Life); total score.	≤ 80	Corresponds to a chronic disease of "mild" severity.[42]
**3. Exclusion of other states that may explain persistent symptoms (screening followed by individual evaluation)**		
**3.1. Screening (individuals must adhere to all in order to remain as cases; non-adherents are subjected to individual evaluation, cf point 3.2)**		
a) HADS-A (Hospital Anxiety and Depression Scale, anxiety subscale).	≤ 10	Screening for anxiety disorder. Score of 8-10 corresponds to "possible" anxiety caseness, 11-15 corresponds to "probable" anxiety caseness.[23] A cut-off of 10 is reported to be optimal in a previous study of screening tools for psychiatric comorbidities in CFS/ME.[43]
b) HADS-D (Hospital Anxiety and Depression Scale, depression subscale).	≤ 10	Screening for depressive disorder. Score of 8-10 corresponds to "possible" depression caseness, 11-15 corresponds to "probable" depression caseness.[25] A cut-off of 10 is reported to be optimal in a previous study of screening tools for psychiatric comorbidities in CFS/ME.[43]
c) KSQ (Karolinska Sleep Questionnaire), average score.	≥ 2	Screening for primary sleep disorders.[44]
d) NT-proBNP.	Upper limit of normality (97.5 percentile)	Screening for cardiac failure. Upper limit (97.5-percentile) age 12-14 years is ≤242; 14-18 years is ≤207; above 18 year is ≤130 (women) and ≤86 (men).[45]
e) SpO_2.	<95%	Screening for respiratory failure.[46]
f) Other disorder/use of medications that may explain persistent symptoms.	No one	As reported in questionnaire, e.g., psychiatric, cardiac, pulmonary, or rheumatic disease.
g) Substance abuse that may explain persistent symptoms.	No one	As reported in questionnaire
h) Finding during clinical examination that may explain persistent symptoms.	No one	E.g., signs of cardiac failure
i) Finding from routine lab screening that may explain persistent symptoms.[b]	No one	E.g., anemia
**3.2. Individual evaluation of potential exclusions (individuals excluded as cases must adhere to all)[c]**		
a) Is a co-existing disorder/aberration causally related to the acute infection (COVID-19)?	No	Organ damage and/or psychological distress caused by the acute infection (COVID-19) itself is NOT a criteria for exclusion according to the WHO case definition (as opposed to the international case definition of PIFS).

© 2023 Selvakumar J et al. *JAMA Network Open.*

b) Is it likely that a co-existing disorder/aberration is causally related to a persisting symptom?	Yes	Example: Chronic asthma may be causally related to persistent shortness of breath and/or coughs. However, chronic asthma cannot readily explain for instance problems of memory and concentration. If the latter problem persist, individuals may still be considered a case of long COVID.
c) Are there other persisting symptoms that cannot be explained from a co-existing disorder/aberration?	No	

[a]The term for long COVID used in the WHO definition is 'post-COVID-19 condition'. [b]Routine lab screening included Blood Haemoglobin, Leukocytes, Differential count, Platelets; Plasma/Serum CRP, Vitamin B_{12}, Folic acid, Ferritin, ALT, GGT, LDH, Albumin, CK, Glucose, HbA_{1C}, Bilirubin, D-dimer, INR, Urea, Creatinine, Natrium, Potassium, Calcium, TSH, Thyroxine. [c]Individual evaluation was performed independently by two researchers using all available information such as recorded data in the present project as well as patients' hospital and GP records. If disagreement about classification, cases were discussed with the principal investigator of the project until consensus was reached.

Operationalisation of the international case definition of post-infective fatigue syndrome (PIFS)[15]

Variable	Criterion	Comment
**1. The international diagnostic definition, main criteria for PIFS (patients must adhere to all)**		
a) Fatigue score at 6 months follow-up.	≥ 4	Total score on the Chalder Fatigue Questionnaire, bimodal scoring (0-0-1-1) of single item.[19] This definition of fatigue caseness has been applied in several previous publications.[38–41]
b) Fatigue score at baseline.	≥ 4	Ensures persistence of fatigue from the acute infectious event.
c) PedsQL (Pediatric Quality of Life); total score.	≤ 76	Corresponds to the "fatigue severely affects daily activities" criterion, and to a chronic disease of "moderate" severity.[42]
a) PEM (Post Exertional Malaise) score.	≥ 2 for at least 1 of 5 items	From the DePaul symptom questionnaire: Five items addresses frequency of PEM symptoms on 5-point Likert scales, where 0 is "never" and 4 is "all the time.[21,22]
**2. The international diagnostic definition, additional criteria for PIFS (criterion h) and i) are merged; patients must then adhere to at least 4 of 8)**		
a) "... experienced fatigue the day after an exertion."	≥ 3	Single item (5-point Likert scale, 3 indicates 1-2 times a week) from the CDC symptom inventory for Chronic Fatigue Syndrome.[20]
b) "… experienced headache."	≥ 3	Single item (5-point Likert scale, 3 indicates 1-2 times a week) from the CDC symptom inventory for Chronic Fatigue Syndrome.[20]
c) "… experienced sore throat."	≥ 3	Single item (5-point Likert scale, 3 indicates 1-2 times a week) from the CDC symptom inventory for Chronic Fatigue Syndrome.[20]
d) "… experienced tender cervical lymphatic nodes."	≥ 3	Single item (5-point Likert scale, 3 indicates 1-2 times a week) from the CDC symptom inventory for Chronic Fatigue Syndrome.[20]
e) "… experienced muscle pain."	≥ 3	Single item (5-point Likert scale, 3 indicates 1-2 times a week) from the CDC symptom inventory for Chronic Fatigue Syndrome.[20]
f) "… experienced multi-joint pain."	≥ 3	Single item (5-point Likert scale, 3 indicates 1-2 times a week) from the CDC symptom inventory for Chronic Fatigue Syndrome.[20]
g) "… experienced unrefreshing sleep."	≥ 3	Single item (5-point Likert scale, 3 indicates 1-2 times a week) from the CDC symptom inventory for Chronic Fatigue Syndrome.[20]
h) "… experienced concentration problems."	≥ 3	Single item (5-point Likert scale, 3 indicates 1-2 times a week) from the CDC symptom inventory for Chronic Fatigue Syndrome.[20]
i) "… experienced memory problems."	≥ 3	Single item (5-point Likert scale, 3 indicates 1-2 times a week) from the CDC symptom inventory for Chronic Fatigue Syndrome.[20]
**3. Exclusion of other states that may explain fatigue (screening followed by individual evaluation)**		
**3.1. Screening (individuals must adhere to all in order to remain as cases; non-adherents are subjected to individual evaluation, cf point 3.2)**		
a) HADS-A (Hospital Anxiety and Depression Scale, anxiety subscore).	≤ 10	Screening for anxiety disorder. Score of 8-10 corresponds to "possible" anxiety caseness, 11-15 corresponds to "probable" anxiety caseness.[25] A cut-off of 10 is reported to be optimal in a previous study of screening tools for psychiatric comorbidities in CFS/ME.[43]
b) HADS-D (Hospital Anxiety and Depression Scale, depression subscore).	≤ 10	Screening for depressive disorder. Score of 8-10 corresponds to "possible" depression caseness, 11-15 corresponds to "probable" depression caseness.[25] A cut-off of 10 is reported to be optimal in a previous study of screening tools for psychiatric comorbidities in CFS/ME.[43]
c) KSQ (Karolinska Sleep Questionnaire, total score).	≥ 2	Screening for primary sleep disorders.[44]
d) NT-proBNP.	Upper limit of normality (97.5 percentile)	Screening for cardiac failure. Upper limit (97.5-percentile) age 12-14 years is ≤242; 14-18 years is ≤207; above 18 year is ≤130 (women) and ≤86 (men).[45]
e) SaO_2.	<95%	Screening for respiratory failure.[46]
f) Other disorder/use of medications that may explain fatigue.	No one	As reported in questionnaire, e.g., psychiatric, cardiac, pulmonary, or rheumatic disease.
g) Substance abuse that may explain fatigue.	No one	As reported in questionnaire

© 2023 Selvakumar J et al. *JAMA Network Open.*

h) Finding during clinical examination that may explain fatigue.	No one	E.g., signs of cardiac failure
i) Finding from routine lab screening that may explain fatigue.[a]	No one	E.g., anemia
**3.2. Individual evaluation of potential exclusions (individuals excluded as cases must adhere to all)[b]**		
a) Is it likely that a co-existing disorder/aberration is causally related to persistent fatigue?	Yes	

[a]Routine lab screening included Blood Haemoglobin, Leukocytes, Differential count, Platelets; Plasma/Serum CRP, Vitamin B_{12}, Folic acid, Ferritin, ALT, GGT, LDH, Albumin, CK, Glucose, HbA_{1C}, Bilirubin, D-dimer, INR, Urea, Creatinine, Natrium, Potassium, Calcium, TSH, Thyroxine. [b]Individual evaluation was performed independently by two researchers using all available information such as recorded data in the present project as well as patients' hospital and GP records. If disagreement about classification, cases were discussed with the principal investigator of the project until consensus was reached.

1.8. Risk factor hypotheses

A total of 78 potential risk factors were defined, based on existing empirical findings of risk factors for long COVID and PIFS, as outlined below. SARS-CoV-2 status (ie., belonging to the SARS-CoV-2-positive or the SARS-CoV-2-negative group at baseline) was considered the primary exposure variable. Background/constitutional factors and observational period characteristics were regarded as potential confounders. The remaining variables were assumed to be either mediating variables related to COVID-19 pathophysiology, or independent exposure variables.

Three of the variables belonging to the clinical symptoms group were defined *de novo* for the present study, based upon the CDC symptom inventory for Chronic Fatigue Syndrome[20] (cf. paragraph 1.6):

- Cognitive symptoms were defined as the sum score across the three items "memory problems", "concentration problems" and "decision making problems"; total range is from 3 to 15, where higher scores imply more cognitive symptoms.
- Respiratory symptoms were defined as the sum score across the two items "dyspnoea" and "coughing"; total range is from 2 to 10, where higher scores imply more respiratory symptoms.
- Autonomic symptoms were defined as the sum score across the three items "orthostatic dizziness", "cold and pale hands" and "feeling alternating warm and cold"; total range is from 3 to 15, where higher scores imply more autonomic symptoms.

Potential risk factors of long COVID and post-infective fatigue syndrome (PIFS) at six months follow-up

Variable group	Variable	Explanations and empirical references
SARS-CoV-2 status	SARS-CoV-2-positive vs. SARS-CoV-2-negative at inclusion	Primary exposure variable
Background and constitutional factors	Sex	Female sex is reported to be a risk factor for long COVID.[39–41,47]
	Age	Increasing age is reported to be a risk factor for long COVID.[41,47,48]
	Body Mass Index (BMI)	Obesity is reported to increase risk of long COVID.[49,50]
	Ethnicity	Classified according to country of birth of the participant and the participant's parents in the present study. In the UK, non-white ethnic minority groups are reported to have lower risk of long COVID.[50]
	Chronic diseases	Asthma as well as poor general health are reported to be risk factors for long COVID.[47,48,50]
Observational period characteristics	Time span from baseline to follow-up	Individuals with PIFS are reported to recover spontaneously over time.[51]
	Immunization against SARS-CoV-2 infection	Vaccination is reported to reduce the risk of long COVID.[52]
Organ function tests and biomarkers	FVC<br>SpO_2	Markers of respiratory aberrations. Persistent microclots in the pulmonary circulation is proposed as a mechanism for long COVID.[53,54]
	D-dimer	Coagulation activation marker. Persisting microclots is a proposed mechanism behind long COVID.[53,54]
	Ferritin	Blood marker of iron storage as well as acute inflammatory responses. Low ferritin level is reported to be a risk factor of long COVID.[55]
	NT-proBNP<br>Troponin T	Blood markers of cardiac involvement. Mild COVID-19 is reported to be associated with elevated Troponin T and NT-proBNP levels, suggesting subtle cardiac damage as a possible mechanism behind long COVID development.[56]
	NfL<br>GFAp	Blood markers of brain injury. Both markers are reported to be elevated in COVID-19; subtle brain injury, in turn, may be implicated in development of long COVID.[57–59]
	Vitamin B_{12}	Vitamin B_{12} is reported to be negatively associated with PIFS following EBV infection.[60]

Immunological markers	Leukocytes (neutrophil, lymphocytes, monocytes, neutrophile:lymphocyte ratio and total count)	High initial white blood cell count associated with long COVID in several studies[61]
	Systemic immune-inflammation index	Defined as (NxP)/L, where N, P and L represent neutrophil, platelet and lymphocyte counts respectively. Used as a prognostic marker in oncology, however was associated with psychological post-COVID symptoms.[62]
	hsCRP	Inflammatory marker. Reported to be an independent risk factor for PIFS following EBV infection.[60]
	GDF-15	Inflammatory marker reported to be associated with poorer clinical outcomes in acute COVID-19.[63]
	TCC/C5b-9	Complement activation marker and hence a part of the innate immune response; reported to be elevated in mild cases of acute COVID-19,[64] and may potentially play a role in long COVID development.[65]
	RANTES/CCL5	T-cell activation marker. Reported to be related to PIFS after EBV-infection.[66]
	MCP-1/CCL2	Monocyte/macrophage activation marker. Reported to be associated with severity of acute COVID-19,[67] and may potentially be related to long COVID development.[68]
	IP-10	Monocyte/macrophage activation marker. Higher levels are reported to be associated with development of long COVID.[69]
	SARS-CoV-2-Anti-RBD	IgG-type antibody directed against the receptor-binding domain (RBD) of the SARS-CoV-2 virus; higher blood titers are reported increase the risk of long COVID.[70]
	Immunoglobulins (IgA, IgM, IgG)	IgG subclass and IgM risk factors for long COVID.[71]
	IL-1β	Inflammatory marker reported to be increased in children with long COVID.[72]
	IL-2	Promoter of T-cell differentiation. Reported to be higher in patients with long COVID[73], and to be related to post-COVID depression.[74]
	IL-4	Induces differentiation of Th0 cells to Th2 cells. Reported to be both lower in patients with long COVID[73], and higher[75]
	IL-7	Hematopoietic growth factor. Associated with long COVID in a prediction model[76]
	IL-8	Chemokine secreted by macrophages and endothelial cells, to induce chemotaxis and phagocytosis. Associated with long COVID[75], and specifically lower grand grip strength in one study[77].
	IL-9	Regulator of hematopoietic cells.
	IL-12	Promoter of T-cell differentiation.
	IL-13	Induces T-cell differentiation, and associated with allergic disease. Increased in long COVID[78]
	IL-17a	Produced by T helper type 17 cells, and proposed in the pathogenesis of immunoinflammatory diseases.[79] Higher in patients with long COVID[73]
	GM-CSF	Growth factor for leukocytes. Increased in long COVID.[78]
	C3bc	Complement C3 – part of innate immunity. Need for further research into how deviations in innate immunity in acute COVID-19 relate to post-COVID-19 syndrome[65]
	bFGF/FGF2	Basic fibroblast growth factor. Higher in patitents with severe acute COVID-19[80], endotheliopathy proposed as contributing to long COVID[81]
	MIP-1 α /CCL3	Acute inflammatory marker involved in the activation of granulocytes. Associated with long COVID in prediction model[76]
	MIP-1 β / CCL4	Among other functions, a chemoattractant for NK-cells. Associated with long COVID in prediction model[76], higher in long COVID.[75]
	Eotaxin /CCL11	Chemotaxis for eosinophils, with a role in neuroinflammation. Proposed as risk factor for LC[82]
	Interferon **γ**	Immunostimulatory effects in both innate and adaptive immunity. Higher in post-COVID depression[74], associated with long COVID[83]
	Tumor necrosis factor α	Increased in long COVID in one report.[84]
Autonomic markers	LF-RRI<br>HF-RRI	Heart rate variability (HRV) indices, providing information on the vagal modulation of the sinoatrial (SA) node (HF-RRI) and the combined effect of vagal and sympathetic SA modulation (LF-RRI). HRV-indices are reported to be implicated in development of PIFS after EBV-infection.[85]
Cognitive function tests	Digit span, total score<br>HVLT-R, immediate recall<br>HVLT-R, delayed recall<br>HVLT-R, recognition index	Digit span total score assess working memory, whereas HVLT-R (Hopkins Verbal Learning Test, revised) assess verbal memory. Cognitive complaints are a main feature of long COVID,[14,86] whereas some cognitive function tests (of verbal memory) are reported to be positively associated with PIFS development after EBV infection.[60]
Clinical symptoms	Chalder Fatigue Questionnaire, total sum score<br>DePaul Symptom Questionnaire, average score of PEM items	Generally, the number of clinical symptoms during acute infection is reported to be predictive of long COVID,[47] and self-reported severity of acute illness is predictive of PIFS development.[60,87] Fatigue, PEM, cognitive symptoms and respiratory symptoms are main features of long COVID.[14,86] Sleep problems, pain and

© 2023 Selvakumar J et al. *JAMA Network Open.*

	Karolinska Sleep Questionnaire, average score<br>Brief Pain Inventory, average pain subscore<br>Cognitive symptoms, total sum score (memory, concentration, decision making)<br>Respiratory symptoms, total sum score (dyspnea, coughing)<br>Autonomic symptoms, total sum score (orthostatic dizziness, cold and pale hands, feeling alternating warm and cold)<br>Hospital Anxiety and Depression Scale, anxiety subscore<br>Hospital Anxiety and Depression Scale, depression subscore<br>Positive and Negative Affect Schedule, total sum score	autonomic symptoms are related to PIFS.[88–90] Symptoms of anxiety and depression as well as mental distress in general are reported to be risk factors for long COVID,[40,48,91] as well as PIFS.[60,92–94]
Psychological traits	NEO-FFI-30, subscore neuroticism<br>Toronto Alexitymia Scale, subscore Difficulty Identifying Feelings<br>Penn State Worry Questionnaire, total score<br>Body Vigilance Scale (BVS), total score	Neuroticism, low emotional awareness, and worrying tendencies are all reported to increase risk of PIFS development;[60,92] also, autonomic hypervigilance is associated with PIFS.[95]
Social and behavioural markers	Average level of physical activity prior to acute infection	Low level of physical activity is reported to be an independent risk factor of PIFS development after EBV infection.[60]
	Socioeconomic level	Classified according to parents' occupation in the present study, following to the international socio-economic index (ISEI)-08.[17,18] Lower education level is possibly associated with lower risk of long COVID.[50]
	Chronic disease, family member	Family stress is reported to increase risk of PIFS.[96]
	UCLA loneliness questionnaire, total sum score	Loneliness is associated with increased mental distress during the COVID-19 pandemic,[97] which in turn is considered a risk factor for PIFS development.[91,92,98]
	Negative life events last year	Negative life events is associated with PIFS development after EBV infection.[60]
	Negative life events prior to last year	

1.9. Statistical analyses

Primary and secondary outcome variables

Long COVID caseness according to the WHO clinical case definition was specified as the primary outcome variable of the present study,[14] whereas post-infective fatigue syndrome (PIFS) caseness according to the international diagnostic definition was designated as a secondary outcome variable.[15] Hence, both outcome variables are dichotomous, having a binominal distribution. The two outcome variables were defined prior to data analysis. However, as the WHO-definition was first proposed in September 2021 whereas recruitment to the present study commenced in December 2020, the primary outcome was not defined in the first version of the Statistical Analysis Plan, but was introduced in a later amendment.[1]

Power analysis

The prevalence of persistent symptoms in the unexposed (SARS-CoV-2-negative) group is uncertain. However, two recent studies of comparable populations reported prevalence rates at 37 % and 21 %, respectively.[99,100] Assuming a prevalence of 30 %, the present study has a power of approximately 80 % to detect a relative risk (RR) of 1.5 (α=0.05, drop-out rate=5 %).

The per protocol data set

The per protocol data set was defined as all individuals completing the investigational program at baseline and six months follow up, except:

- SARS-CoV-2-negative individuals at baseline with reported SARS-CoV-2 infection in the observational period or anti-SARS-CoV-2 antibodies (any type for unvaccinated, anti-nucleocapsid for vaccinated) detected at six months follow-up.
- SARS-CoV-2-positive individuals at baseline with reported novel SARS-CoV-2 infection in the observational period, or increased anti-nucleocapsid antibody-titer at six months as compared to baseline.

These individuals were thought to violate a fundamental premise of the study (one acute SARS-CoV-2 infection in the SARS-CoV-2-positive group, no acute SARS-CoV-2 infection in the SARS-CoV-2-negative group), and were therefore excluded from all further analyses. In the per-protocol data set, laboratory values below lower detection limit (LDL) were replaced with a random value in the interval between zero and LDL for each specific analysis. Otherwise, no missing data were imputed.

Data set for sensitivity analyses #1 – imputation of missing values

For sensitivity analyses purposes, an imputed data set was constructed based upon the per protocol data set. Missing values in the independent variables were substituted with the mean or median, based on assumed distribution as reported in Table 1 in the main manuscript. Hence, no imputation was performed for individuals lost to follow-up or excluded due to suspected

© 2023 Selvakumar J et al. *JAMA Network Open.*

SARS-CoV-2 infection, as these individuals were not part of the per-protocol data set. The Statistical Analyses Plan for LoTECA generally recommended the technique of multiple imputation for missing data points.[1] However, in the present study, this technique was not considered feasible as it would have created datasets with potential differences in caseness allocation, factor structures and multivariable modelling, making it difficult (or even impossible) to obtain an aggregated final result.

Sensitivity analyses #2 – removal of potential bias
The impact of immunisation against SARS-CoV-2 for development of long COVID is unclear. Some data suggests a protective effect,[52] whereas others have speculated that vaccination may actually trigger PIFS in vulnerable individuals, as has been reported after immunization against other microorganisms.[101] Also, common side effects in the days after vaccination (chills, malaise, etc.) may mimic the symptoms of long COVID. Hence, vaccination introduces a potential bias.

The reason for requiring a SARS-CoV-2 diagnostic test, which was the primary entry criterion for the present study, might have been clinical symptoms caused by an infection other than COVID-19. The only endemic infection in Norway that has a documented association to PIFS development is EBV-infection, causing the clinical picture of infectious mononucleosis.[60] Thus, acute EBV-infection among the participants of the present study may bias the results. As described in paragraph 1.7, some individuals at six months follow-up could not be classified with certainty according to the WHO case definition of long COVID and/or the international case definition of PIFS. These uncertain classifications also introduce a potential source of bias.

In order to construct a dataset for sensitivity analyses minimizing the potential sources of biases, the following exclusions from the per-protocol data set were performed:

- Individuals that received vaccination prior to inclusion or less than five days prior to the six month follow-up appointment
- Individuals with EBV serology results suggesting acute EBV infection at inclusion or during the six months observational period, or for which an early infection at the six-month follow-up could not be ruled out.
- Individuals with uncertain caseness classification at six months follow-up.

The number of individuals with uncertain classification differed between long COVID and PIFS, and thus the exclusions above resulted in two different datasets. For long COVID, the dataset consisted of a total of 407 individuals (n=335 in the SARS-CoV-2-positive group and n=72 in the SARS-CoV-2-negative group); while for PIFS, the dataset consisted of a total of 420 individuals (n=343 in the SARS-CoV-2-positive group and n=77 in the SARS-CoV-2-negative group).

As an extra quality control, an additional sensitivity analysis of prevalence and the final multivariate regression model were performed, removing individuals in the SARS-CoV-2-negative group with a general infection symptoms score ≥ 11 at baseline alongside the exclusions listed above. This score is computed as the sum across five single items charting the symptoms of fever/chills, sore throat, headaches, muscle ache and fatigue after exercise, and has a total range from 5 – 25.[64] The cut-off was chosen based on a previous study of EBV-infected adolescents and healthy (i.e. non-infectious) controls where an identical inventory was applied.[60] Removing individuals in the SARS-CoV-2-negative group with general infectious symptom score ≥ 11 resulted in a distribution very similar to the healthy control group of the previous study, hence minimising the possibility of bias from another acute infection than COVID-19. The resulting dataset for long COVID consisted of 393 individuals (n=335 in the SARS-CoV-2-positive group and n=63 in the SARS-CoV-2-negative group), and the dataset for PIFS consisted of 409 individuals (n=343 in the SARS-CoV-2-positive group and n=66 in the SARS-CoV-2-negative group).

Bivariate analyses
The 78 hypothesized baseline risk factors (cf. paragraph 1.8) were assess for collinearity applying non-parametric correlation analyses (Spearman's *rho*). All variables within the clinical symptoms group were strongly correlated with each other, as were all variables within the psychological traits group. Hence, principal component analysis (PCA) within each of these two groups were used to reduce dimensionality, and the principal component from each analysis were extracted. The relationships between each of the hypothesized baseline factors (including the two PCA-derived components) and the two dependent variables (caseness according to the WHO case definition of long COVID and the international case definition of PIFS respectively) were explored in separate univariate regression analyses. First, generalized linear modelling (GLM) using binominal distribution and log-link function was used. However, as the model failed to converge for several of the analyses, an alternative approach using Poisson-distribution and log-link function with robust error variances was successfully applied. Analyses were first conducted using the per-protocol data set. An identical approach was applied separately on the two different data sets for sensitivity analyses.

Multivariable analyses

© 2023 Selvakumar J et al. *JAMA Network Open.*

As the primary exposure variable (SARS-CoV-2 status at baseline) was not associated with either long COVID or PIFS, the primary hypothesis of the present study was not supported. Hence, adjusting the association between the primary exposure variable and the two outcome variables were not seen as relevant. Exploratory multivariable analyses were therefore carried out with the aim of identifying other potential risk factors of long COVID and PIFS.

The multivariable modelling procedure also featured generalized linear models with a modified Poisson approach (log-link function with robust error variances). All independent variables in the categories SARS-CoV-2 status, background/constitutional factors, and observational period characteristics were retained in the modelling throughout (cf. paragraph 1.8). Of the remaining variables, all with a p-value ≤ 0.25 (based on the likelihood-ratio test) in the bivariate GLM analyses were included in the first modelling step, except for clinical symptoms and psychological traits where the two PCA-derived components replaced the original variables. Then, in order to obtain a more parsimonious model, the variable with the highest p-value was removed if it did not substantially alter the overall goodness-of-fit of the model, defined as a change in the Akaike Information Criteria (AIC) > 2. Iterative removal of variables one-by-one was performed adhering to the same rule, resulting in a reduced model where only variables with a p-value ≤ 0.05 remained. Then, removed variables were re-introduced to the reduced model one at a time. Variables with a p-value ≤ 0.05 when added individually to the reduced model, were added back in the model. Then, the model was iteratively reduced once again, until only variables with a p-value ≤ 0.05 remained.

Again, the analyses were first carried out within the per protocol data set, followed by identical procedures within the two data sets for sensitivity analyses. As the modelling was considered exploratory, p-values were not adjusted for test multiplicity.

© 2023 Selvakumar J et al. *JAMA Network Open.*

Table S1. Results of all SARS-CoV-2 PCR-tests performed between December 24., 2020 and May 18., 2021 at Akershus University Hospital and Fürst Medical Laboratory, with respect to age and sex.

	SARS-CoV-2 negative			SARS-CoV-2 positive			Total number tested		
	Male	*Female*	*Both*	*Male*	*Female*	*Both*	*Male*	*Female*	*Both*
**Age group**									
**12-13**	8463	7907	16370	390	354	744	8853	8261	17114
**14-15**	9172	8474	17646	409	368	777	9581	8842	18423
**16-17**	8319	9413	17732	444	390	834	8763	9803	18566
**18-19**	10477	12342	22819	495	457	952	10972	12799	23771
**20-21**	11042	13063	24105	466	467	933	11508	13530	25038
**22-25**	21397	25129	46526	812	860	1672	22209	25989	48198
**All**	68870	76328	145198	3016	2896	5912	71886	79224	151110

SARS-CoV-2= Severe acute respiratory syndrome coronavirus 2; PCR=Polymerase chain reaction

© 2023 Selvakumar J et al. *JAMA Network Open.*

Table S2. Attritional analyses. SARS-CoV-2-positive in background population, proportions invited to participate, and proportions included in study, with respect to age and sex.

	**Background population**		**Invited to participate**			**Included in study**		
	Male	*Female*	*Male Proportion of population*	*Female Proportion of population*	*Test of proportions p-value*	*Male Proportion of invited*	*Female Proportion of invited*	*Test of proportions p-value*
**Age group**								
**12-13**	390	354	0.44 (172/390)	0.45 (159/172)	0.824	0.19 (33/172)	0.24 (38/159)	0.297
**14-15**	409	368	0.49 (202/409)	0.44 (161/202)	0.116	0.19 (38/202)	0.19 (30/161)	0.965
**16-17**	444	390	0.44 (196/444)	0.45 (176/196)	0.775	0.17 (33/196)	0.2 (36/176)	0.370
**18-19**	495	457	0.41 (204/495)	0.39 (176/204)	0.395	0.09 (19/204)	0.2 (36/176)	0.002
**20-21**	466	467	0.35 (164/466)	0.41 (193/164)	0.054	0.1 (17/164)	0.22 (43/193)	0.003
**22-25**	812	860	0.24 (198/812)	0.29 (250/198)	0.031	0.1 (20/198)	0.25 (62/250)	<0.001
**All**	3016	2896	0.38 (1136/3016)	0.39 (1115/1136)	0.508	0.14 (160/1136)	0.22 (245/1115)	<0.001

SARS-CoV-2= Severe acute respiratory syndrome coronavirus 2

© 2023 Selvakumar J et al. *JAMA Network Open.*

Table S3. Attritional analyses. Characteristics of potential baseline risk factors and their univariate associations (Poisson regression with log-link and robust error variances) to being 'lost to follow up'.

	Baseline characteristics		Relative risk of being lost to follow-up		
	All cases who attended six month follow-up (n=467)	*Cases lost to follow-up before six months (n=26)*	*Relative risk (CI)[a]*	*p-value[b]*	*Adjusted p-value[c]*
**SARS-CoV-2 status**					
SARS-CoV-2-positive at baseline – no. (%)	382 (81.8)	22 (84.6)	1.21 (0.47, 4.00)	0.711	1.000
**Background and constitutional factors**					
Female sex – no. (%)	284 (60.8)	20 (76.9)	2.07 (0.90, 5.51)	0.089	1.000
Age, years – mean (CI)	17.94 (17.61, 18.27)	19.56 (17.93, 21.18)	1.12 (1.01, 1.25)	0.031	1.000
BMI, z-score[d] – mean (CI)	0.44 (0.34, 0.55)	0.75 (0.33, 1.18)	1.24 (0.90, 1.71)	0.185	1.000
Ethnicity non-European – no. (%)	90 (19.3)	11 (42.3)	2.84 (1.30, 6.04)	0.010	0.761
Any comorbidity – no. (%)	107 (22.9)	3 (11.5)	0.44 (0.11, 1.23)	0.126	1.000
**Observational period characteristics**					
Time span between baseline and follow-up, days – median (range)	193 (164-326)	NA	NA	NA	NA
Immunisation against SARS-CoV-2[e] – no. (%)	7 (1.5)	2 (7.7)	4.48 (0.76, 14.70)	0.087	1.000
**Organ function tests/biomarkers**					
FVC, % of predicted[f] – mean (CI)	99.7 (98.7, 100.7)	101.0 (97.1, 104.9)	1.01 (0.97, 1.05)	0.548	1.000
SpO_2, % – mean (CI)	98.7 (98.6, 98.8)	98.1 (97.6, 98.6)	0.68 (0.49, 0.94)	0.022	1.000
NT-pBNP, ng/L – median (CI)	35 (31, 37)	38 (26, 61)	1.00 (0.99, 1.01)	0.420	1.000
Troponin T, ng/L – median (CI)	4 (4, 4)	3.41 (1.36, 4.00)	0.81 (0.67, 0.96)	0.016	1.000
NfL, pg/mL – mean (CI)	4.63 (4.30, 4.96)	5.29 (3.85, 6.72)	1.03 (0.93, 1.07)	0.475	1.000
GFAp, pg/mL – mean (CI)	67.44 (62.88, 72.0)	74.96 (54.52, 95.41)	1.00 (0.99, 1.01)	0.523	1.000
D-dimer[g], mg/L – median (CI)	0.18 (0.16, 0.19)	0.23 (0.20, 0.30)	3.73 (0.45, 23.01)	0.215	1.000
Ferritin, µg/L – median (CI)	66 (61, 69)	52 (24, 85)	1.00 (0.99, 1.01)	0.786	1.000
Vitamin B_{12}, pmol/L – mean (CI)	439.63 (424.12, 455.15)	431.96 (356.72, 507.20)	1.00 (1.00, 1.00)	0.822	1.000
**Immunological markers**					
Blood Leukocyte count, 10^9 cells/L - mean (CI)	5.87 (5.73, 6.01)	6.30 (5.80, 6.81)	1.17 (0.92, 1.42)	0.189	1.000
Blood Lymphocyte count, 10^9 cells/L - mean (CI)	2.11 (2.06, 2.17)	2.32 (2.10, 2.52)	1.63 (0.88, 2.85)	0.118	1.000
Blood Monocyte count, 10^9 cells/L - mean (CI)	0.448 (0.434, 0.462)	0.52 (0.45, 0.58)	1.16 (1.01, 1.32)	0.030	1.000
Blood Neutrophil count, 10^9 cells/L - mean (CI)	3.14 (3.04, 3.25)	3.30 (2.84, 3.77)	1.11 (0.79, 1.50)	0.515	1.000
Neutrophil-to-Lymphocyte ratio – mean (CI)	1.57 (1.51, 1.63)	1.51 (1.24, 1.79)	0.88 (0.45, 1.56)	0.677	1.000
Systemic immune-inflammation index - median (CI) [h]	408 (389, 427)	411 (322, 501)	1.00 (1.00, 1.00)	0.938	1.000
hsCRP[i], mg/L – median (CI)	0.89 (0.72, 1.10)	2.62 (1.47, 5.83)	1.70 (1.26, 2.32)	<.001	0.030
GDF15, ng/mL – mean (CI)	0.41 (0.39, 0.42)	0.50 (0.36, 0.65)	4.36 (1.12, 11.44)	0.036	1.000
TCC/C5b-9, CAU/mL – median (CI)	0.16 (0.14, 0.17)	0.23 (0.13, 0.32)	1.01 (0.52, 1.18)	0.950	1.000
RANTES/CCL5[j], pg/mL – median (CI)	264.16 (237.23, 292.15)	303.11 (190.07, 469.65)	1.06 (0.67, 1.54)	0.800	1.000
MCP-1/CCL2, pg/mL – mean (CI)	13.02 (12.45, 13.58)	13.46 (9.96, 16.97)	1.01 (0.95, 1.07)	0.730	1.000
IP-10, pg/mL – mean (CI)	157.02 (143.39, 164.65)	148.69 (127.44, 169.94)	1.00 (0.99, 1.00)	0.591	1.000
SARS-CoV-2-Anti-RBD, BAU/mL – median (CI)	129.58 (74.80, 972.83)	1004.5 (30.50, 1586.30)	1.00 (1.00, 1.00)	0.719	1.000
Plasma total IgG, g/L - mean (CI)	11.0 (10.8, 11.2)	11.5 (10.8, 12.3)	1.11 (0.93, 1.31)	0.242	1.000
Plasma total IgM, g/L - mean (CI)	1.24 (1.19, 1.29)	1.20 (0.97, 1.42)	0.86 (0.39, 1.75)	0.692	1.000
Plasma total IgA, g/L - mean (CI)	1.68 (1.61, 1.75)	2.02 (1.62, 2.41)	1.56 (1.00, 2.31)	0.050	1.000
Plasma IL-1β, pg/mL – median (CI)	0.47 (0.23, 0.63)	0.20 (0.01, 0.86)	1.19 (0.91, 1.39)	0.165	1.000
Plasma IL-2, pg/mL - median (CI)	0.690 (0.470, 0.780)	0.030 (0.017, 1.66)	1.11 (0.96, 1.20)	0.136	1.000

© 2023 Selvakumar J et al. *JAMA Network Open.*

Plasma IL-4, pg/mL - median (CI)	1.33 (1.25, 1.41)	1.25 (1.01, 1.50)	1.17 (0.77, 1.53)	0.424	1.000
Plasma IL-7, pg/mL - median (CI)	11.5 (10.0, 12.6)	9.39 (5.65, 18.7)	1.01 (0.98, 1.04)	0.447	1.000
Plasma IL-8, pg/mL - median (CI)	0.550 (0.240, 0.690)	0.550 (0.116, 1.91)	1.05 (0.996, 1.08)	0.064	1.000
Plasma IL-9, pg/mL - median (CI)	68.9 (60.6, 79.1)	77.0 (39.6, 129.7)	1.00 (1.00, 1.00)	0.323	1.000
Plasma IL-12, pg/mL - median (CI)	1.38 (1.37, 1.49)	1.38 (0.17, 4.84)	1.03 (1.00, 1.06)	0.059	1.000
Plasma IL-13, pg/mL - median (CI)	0.270 (0.260, 0.290)	0.260 (0.019, 0.450)	1.09 (0.84, 1.26)	0.429	1.000
Plasma IL-17A, pg/mL - median (CI)	1.62 (1.35, 1.99)	1.31 (0.69, 2.62)	1.07 (0.89, 1.22)	0.461	1.000
Plasma TNF, pg/mL - median (CI)	6.73 (6.26, 7.81)	5.78 (4.54, 10.5)	1.01 (0.95, 1.04)	0.787	1.000
Plasma IFN-γ, pg/mL - median (CI)	1.14 (1.02, 1.30)	1.02 (0.55, 1.49)	1.02 (1.00, 1.03)	0.071	1.000
Plasma Eotaxin-1/CCL11, pg/mL - median (CI)	14.1 (13.6, 14.9)	13.5 (10.8, 16.0)	0.95 (0.87, 1.01)	0.134	1.000
Plasma MIP-1α, pg/mL - median (CI)	0.77 (0.77, 0.82)	0.82 (0.67, 0.96)	1.99 (0.90, 3.66)	0.084	1.000
Plasma MIP-1β, pg/mL - median (CI)	24.9 (22.4, 26.7)	26.3 (17.1, 48.3)	1.00 (0.99, 1.01)	0.485	1.000
Plasma GM-CSF, pg/mL - median (CI)	0.11 (0.03, 0.11)	0.02 (0.01, 0.34)	1.07 (0.98, 1.13)	0.118	1.000
Plasma bFGF, pg/mL - median (CI)	2.40 (2.30, 3.14)	1.78 (1.53, 8.51)	1.03 (1.00, 1.04)	0.079	1.000
Plasma C3bc, ng/mL - median (CI)	3.64 (3.42, 3.82)	4.15 (3.32, 4.57)	1.16 (0.94, 1.39)	0.171	1.000
**Autonomic markers**					
LF-RRI[i], ms^2 – median (CI)	642 (582, 744)	486.5 (319.0, 997.0)	0.89 (0.60, 1.31)	0.554	1.000
HF-RRI[i], ms^2 – median (CI)	809.96 (718.0, 923.15)	567.5 (334.0, 886.0)	0.78 (0.56, 1.09)	0.146	1.000
**Cognitive function tests**					
Digit span[k], total score – median (CI)	15.12 (14.80, 15.44)	13.81 (12.60, 15.01)	0.89 (0.79, 1.00)	0.053	1.000
Immediate recall[l], score 0 to 36 – median (CI)	24.59 (24.22, 24.97)	22.38 (20.60, 24.17)	0.89 (0.81, 0.97)	0.010	0.734
Delayed recall[l], score 0 to 12 – median (CI)	8.68 (8.50, 8.86)	8.08 (7.29, 8.87)	0.87 (0.72, 1.05)	0.139	1.000
Recognition index[m], score 0 to 12 – median (CI)	12 (11, 12)	12 (11, 12)	0.88 (0.63, 1.30)	0.495	1.000
**Clinical symptoms**					
Fatigue[n], score 0 to 33 – mean (CI)	15.61 (15.09, 16.14)	16.65 (14.36, 18.94)	1.03 (0.97, 1.10)	0.368	1.000
Post-exertional malaise[o], score 0 to 100 – median (CI)	20 (15, 20)	17.5 (5, 40)	1.01 (0.99, 1.02)	0.494	1.000
Sleep problems[p], score 1 to 6 – mean (CI)	4.01 (3.90, 4.11)	3.79 (3.25, 4.34)	0.86 (0.62, 1.20)	0.358	1.000
Pain[q], score 1 to 10 – median (CI)	2.25 (2.00, 2.50)	2.63 (1.75, 4.00)	1.28 (0.98, 1.64)	0.071	1.000
Cognitive symptoms[r], score 3 to 15 – median (CI)	6 (5, 6)	5.5 (4.0, 9.0)	0.99 (0.87, 1.11)	0.884	1.000
Respiratory symptoms[s], score 2 to 10 – median (CI)	4 (4, 4)	4 (3, 5)	1.09 (0.91, 1.28)	0.335	1.000
Autonomic symptoms[t], score 2 to 10 - median (CI)	4 (5, 6)	5 (4, 8)	1.02 (0.89, 1.15)	0.804	1.000
Symptoms of anxiety[u], score 0 to 21 – median (CI)	6 (5, 6)	5.5 (3.0, 9.0)	1.00 (0.91, 1.09)	0.956	1.000
Symptoms of depression[u], score 0 to 21 – median (CI)	3 (3, 4)	4 (3, 6)	1.03 (0.94, 1.13)	0.515	1.000
Negative emotions[v], score 5 to 25 – median (CI)	10 (9, 11)	8 (5, 11)	0.4 (0.86, 1.02)	0.161	1.000
Principal Component: Symptom severity[w] – mean (CI)	-0.01 (-0.10, 0.09)	0.13 (-0.31, 0.56)	1.13 (0.78, 1.61)	0.517	1.000
**Psychological traits**					
Neuroticism[x], score 0 to 24 – median (CI)	6 (5, 7)	3.5 (1.0, 9.0)	0.96 (0.90, 1.02)	0.234	1.000
Emotional awareness[y], score 7 to 35 – median (CI)	13 (12, 14)	14.5 (11.0, 18.0)	1.00 (0.95, 1.06)	0.911	1.000
Worrying tendencies[z], score 16 to 80 – mean (CI)	45.54 (44.23, 46.84)	41.96 (35.81, 48.11)	0.98 (0.95, 1.01)	0.207	1.000
Body vigilance[aa], score 0 to 40 – mean (CI)	11.99 (11.31, 12.68)	12.94 (9.33, 16.55)	1.02 (0.97, 1.07)	0.534	1.000
Principal component: Emotional maladjustment[ab] – mean (CI)	0.01 (-0.09, 1.00)	-0.12 (-0.54, 0.30)	0.89 (0.59, 1.29)	0.533	1.000
**Social/behavioural markers**					
Average level of physical activity prior to acute infection[ac], score 1 to 10 – mean (CI)	6.37 (6.17, 6.58)	5.15 (4.22, 6.09)	0.79 (0.67, 0.94)	0.007	0.528
Socioeconomic level ISEI-08[ad], score 10 to 90 – median (CI)	63.33 (60.29, 68.54)	62.45 (35.7, 76.49)	1.00 (0.98, 1.02)	0.796	1.000
Family member with chronic disease[ae] – no. (%)	153 (33.9)	8 (30.8)	0.87 (0.37, 1.90)	0.740	1.000
Loneliness[af], score 20-80 – mean (CI)	37.98 (36.99, 38.97)	38.31 (33.73, 42.89)	1.00 (0.97, 1.04)	0.879	1.000

© 2023 Selvakumar J et al. *JAMA Network Open.*

Negative life events last 12 months[ag], impact score – median (CI)	2 (2, 2)	1.5 (0, 3)	0.97 (0.87, 1.06)	0.525	1.000
Negative life events prior to last 12 months[ag], impact score – median (CI)	0 (0, 1)	0 (0, 0)	0.73 (0.52, 0.94)	0.013	1.000

CI=95% Confidence interval; NA=Not applicable; SARS-CoV-2= Severe acute respiratory syndrome coronavirus 2; BMI=Body mass index; FVC=Forced vital capacity; SpO_2=Peripheral oxygen saturation; NT-pBNP=N-terminal pro-brain natriuretic peptide; NfL=Neurofilament light chain; GFAp=Glial fibrillary acidic protein; hsCRP=high-sensitive assay of C-reactive protein; GDF-15=Growth/differentiation factor 15; IL=Interleukin; TCC=Terminal complement complex; CAU=Complement arbitrary units; RANTES=Regulated on activation, normal T-cell expressed and secreted; MCP=Monocyte chemotactic protein; IP=Interferon gamma-induced protein; RBD= Receptor binding domain; BAU=Binding antibody units; LF-RRI=Low frequency power of heart rate variability; HF-RRI=High-frequency power of heart rate variability; ISEI-08=International Socioeconomic Index 2008. [a]95% Profile likelihood based confidence intervals. [b]Likelihood ratio p-values. [c]Bonferroni-adjusted for test multiplicity. [d]Standardised score calculated according to World Health Organisation 2006 Child Growth Standards for ages 12-19; for participants above this age, reference values for 19-year-olds were used. [e]One or more doses of immunisation against SARS-CoV-2. [f]The Global Lung Function Initiative 2012 reference values were used to calculate predicted values.[4] [g]Square-root-transformed variable was used for regression analysis. [h]Defined as (NxP)/L, where N, P and L represent neutrophil, platelet and lymphocyte counts respectively. [i]Ln-transformed variable was used for regression analyses. [j]Fifth-root-transformed variable was used for regression analyses. [k]From the Wechsler Intelligence Scales for Children revised; higher score implies better short-term memory. [l]From the Hopkins Verbal Learning Test revised (HVLT-R); higher scores imply better immediate and delayed recall of words, respectively. [m]From the HVLT-R; higher score implies better recognition of words. [n]From the Chalder Fatigue Questionnaire; higher score implies more fatigue. [o]From the DePaul Symptom Questionnaire; higher score implies more post-exertional malaise. [p]From the Karolinska Sleep Questionnaire; higher score implies better sleep. [q]From the Brief Pain Inventory, higher score implies more pain. [r]Self-developed, aggregated score for problems with 'memory', 'concentration', and 'decision making'; higher score implies more symptoms. [s]Self-developed, aggregated score for symptoms 'cough' and 'dyspnoea'; higher score implies more symptoms. [t]Self-developed, aggregated score for symptoms 'dizziness', 'cold and pale hands', 'feeling alternately warm and cold'; higher score implies more symptoms. [u]From the anxiety and depression subscales, respectively, of the Hospital Anxiety and Depression Scale; higher scores imply more symptoms. [v]From the Positive and Negative Affect Schedule; higher score implies more negative emotions. [w]The main component extracted by Principal Component Analysis of the 10 clinical symptoms variables, labelled 'symptom severity'. [x]From the NEO-Five-Factor-Inventory-30; higher scores implies more neuroticism. [y]From the Toronto Alexithymia Scale; higher score implies more difficulty identifying feelings. [z]From the Penn State Worry Questionnaire; higher score implies more worrying. [aa]From the Body Vigilance Scale; higher score implies being more attentive to bodily sensations. [ab]The main component extracted by Principal Component Analysis of the four psychological traits variables, labelled 'emotional maladjustment'. [ac]Self-developed; higher score implies more physical activity. [ad]The ISEI-08 score of the parent with the highest score; higher score implies higher socioeconomic status. [ae]Having a sibling or parent affected by chronic disease. [af]From the University of California, Los Angeles, Loneliness Scale; higher score implies more loneliness. [ag]From the Life Event Checklist; higher score implies more negative impact of past life events.

© 2023 Selvakumar J et al. *JAMA Network Open.*

Table S4. Analyses of missing data. Characteristics of baseline independent variables and their association to complete cases at six months follow-up.

		**Baseline characteristics**		**Relative risk of being a complete case at six months**		
	Cases with available data for variable, N (%)	*All cases with available data for variable (n=N)*	*Complete cases only (n=307)*	*Relative risk (CI)*[a]	*p-value*[b]	*Adjusted p-value*[c]
**SARS-CoV-2 status**						
SARS-CoV-2-positive at baseline – no. (%)	467 (100)	382 (81.8)	247 (80.5)	0.92 (0.88, 1.08)	0.304	1.000
**Background and constitutional factors**						
Female sex – no. (%)	467 (100)	284 (60.8)	196 (63.8)	1.14 (0.99, 1.31)	0.063	1.000
Age, years – mean (CI)	467 (100)	17.94 (17.61, 18.27)	18.2 (17.8, 18.6))	1.02 (1.00, 1.04)	0.029	1.000
BMI, z-score[d] – mean (CI)	466 (99.8)	0.44 (0.34, 0.55)	0.54 (0.42, .066)	1.07 (1.01, 1.14)	0.015	1.000
Ethnicity non-European – no. (%)	467 (100)	90 (19.3)	50 (16.3)	0.82 (0.68, 0.97)	0.021	1.000
Any comorbidity – no. (%)	453 (97.0)	107 (23.6)	70 (22.8)	0.96 (0.82, 1.10)	0.551	1.000
**Observational period characteristics**						
Time span between baseline and follow-up, days – median (range)	467 (100)	193 (164-326)	199.9 (197.6, 202.2)	1.09 (0.62, 1.76)	0.753	1.000
Immunisation against SARS-CoV-2[e] – no. (%)	467 (100)	7 (1.5)	5 (1.6)	1.00 (1.00, 1.00)	0.550	1.000
**Organ function tests/biomarkers**						
FVC, % of predicted[f] – mean (CI)	400 (85.7)	99.7 (98.7, 100.7)	0.859 (0.852, 0.867)	1.11 (0.48, 2.56)	0.812	1.000
SpO_2, % – mean (CI)	465 (99.6)	98.7 (98.6, 98.8)	98.7 (98.5, 98.8)	1.00 (0.94, 1.06)	0.997	1.000
NT-pBNP, ng/L – median (CI)	439 (94.0)	35 (31, 37)	34.0 (30.0, 36.0)	1.00 (1.00, 1.00)	0.134	1.000
Troponin T, ng/L – median (CI)	447 (95.7)	4 (4, 4)	4.0 (4.0, 4.0)	0.99 (0.96, 1.10)	0.221	1.000
NfL, pg/mL – mean (CI)	461 (98.7)	4.63 (4.30, 4.96)	4.70 (4.26, 5.15)	1.01 (0.99, 1.02)	0.536	1.000
GFAp, pg/mL – mean (CI)	461 (98.7)	67.44 (62.88, 72.0)	67.3 (61.0, 73.1)	1.00 (1.00, 1.00)	0.800	1.000
D-dimer[g], mg/L – median (CI)	456 (97.6)	0.18 (0.16, 0.19)	0.178 (0.154, 0.201)	0.91 (0.62, 1.32)	0.613	1.000
Ferritin, µg/L – median (CI)	437 (93.6)	66 (61, 69)	67.0 (63.0, 72.0)	1.00 (1.00, 1.00)	0.049	1.000
Vitamin B_{12}, pmol/L – mean (CI)	443 (94.9)	439.63 (424.12, 455.15)	431 (413, 449)	1.00 (1.00, 1.00)	0.109	1.000
**Immunological markers**						
Blood Leukocyte count, 10^9 cells/L - mean (CI)	427 (91.4)	5.87 (5.73, 6.01)	5.87 (5.70, 6.04)	1.00 (0.96, 1.04)	0.941	1.000
Blood Lymphocyte count, 10^9 cells/L - mean (CI)	437 (93.6)	2.11 (2.06, 2.17)	2.11 (2.04, 2.18)	0.99 (0.89, 1.10)	0.824	1.000
Blood Monocyte count, 10^9 cells/L - mean (CI)	438 (93.8)	0.448 (0.434, 0.462)	0.448 (0.431, 0.466)	1-02 (0.68, 1.53)	0.917	1.000
Blood Neutrophil count, 10^9 cells/L - mean (CI)	437 (93.6)	3.14 (3.04, 3.25)	3.13 (3.01, 3.26)	0.99 (0.94, 1.05)	0.751	1.000
Neutrophil-to-Lymphocyte ratio – mean (CI)	437 (93.6)	1.57 (1.51, 1.63)	1.57 (1.49, 1.64)	1.00 (0.91, 1.10)	0.938	1.000
Systemic immune-inflammation index - median (CI) [h]	428 (91.6)	408 (389, 427)	403 (381, 425)	1.00 (1.00, 1.00)	0.388	1.000
hsCRP[i], mg/L – median (CI)	451 (96.1)	0.89 (0.72, 1.10)	0.98 (0.72, 1.20)	1.02 (0.97, 1.07)	0.482	1.000
GDF15, ng/mL – mean (CI)	451 (96.6)	0.41 (0.39, 0.42)	0.399 (0.380, 0.417)	0.80 (0.53, 1.17)	0.261	1.000
TCC/C5b-9, CAU/mL – median (CI)	451 (96.6)	0.16 (0.14, 0.17)	0.170 (0.150, 0.190)	1.02 (0.97, 1.06)	0.500	1.000
RANTES/CCL5[j], pg/mL – median (CI)	451 (96.6)	264.16 (237.23, 292.15)	266 (243, 309)	1.00 (0.93, 1.08)	0.958	1.000
MCP-1/CCL2, pg/mL – mean (CI)	451 (96.6)	13.02 (12.45, 13.58)	13.0 (12.4, 13.7)	1.00 (0.99, 1.01)	0.886	1.000
IP-10, pg/mL – mean (CI)	451 (96.6)	157.02 (143.39, 164.65)	156 (148, 164)	1.00 (1.00, 1.00)	0.716	1.000
SARS-CoV-2-Anti-RBD, BAU/mL – median (CI)	461 (98.7)	129.58 (74.80, 972.83)	249 (88.5, 1048)	1.00 (1.00, 1.00)	0.125	1.000
Plasma total IgG, g/L - mean (CI)	450 (96.94)	11.0 (10.8, 11.2)	10.9 (10.7, 11.2)	0.98 (0.95, 1.01)	0.141	1.000
Plasma total IgM, g/L - mean (CI)	452 (96.8)	1.24 (1.19, 1.29)	1.23 (1.17, 1.29)	0.97 (0.81, 1.10)	0.658	1.000
Plasma total IgA, g/L - mean (CI)	451 (96.6)	1.68 (1.61, 1.75)	1.71 (1.63, 1.80)	1.06 (0.97, 1.14)	0.204	1.000
Plasma IL-1β, pg/mL – median (CI)	451 (96.6)	0.47 (0.23, 0.63)	0.470 (0.220, 0.630)	1.00 (0.93, 1.08)	0.951	1.000
Plasma IL-2, pg/mL - median (CI)	451 (96.6)	0.690 (0.470, 0.780)	0.690 (0.470, 0.780)	1.00 (0.96, 1.04)	0.936	1.000

© 2023 Selvakumar J et al. *JAMA Network Open.*

Plasma IL-4, pg/mL - median (CI)	451 (96.6)	1.33 (1.25, 1.41)	1.33 (1.25, 1.46)	1.00 (0.92, 1.08)	0.915	1.000
Plasma IL-7, pg/mL - median (CI)	451 (96.6)	11.5 (10.0, 12.6)	12.2 (11.4, 12.6)	1.01 (1.00, 1.01)	0.102	1.000
Plasma IL-8, pg/mL - median (CI)	451 (96.6)	0.550 (0.240, 0.690)	0.550 (0.240, 0.800)	1.01 (0.98, 1.03)	0.682	1.000
Plasma IL-9, pg/mL - median (CI)	451 (96.6)	68.9 (60.6, 79.1)	70.8 (62.1, 82.2)	1.00 (1.00, 1.00)	0.859	1.000
Plasma IL-12, pg/mL - median (CI)	451 (96.6)	1.38 (1.37, 1.49)	1.38 (1.37, 1.50)	1.00 (0.99, 1.01)	0.913	1.000
Plasma IL-13, pg/mL - median (CI)	451 (96.6)	0.270 (0.260, 0.290)	0.270 (0.260, 0.320)	1.03 (0.98, 1.08)	0.181	1.000
Plasma IL-17A, pg/mL - median (CI)	451 (96.6)	1.62 (1.35, 1.99)	1.62 (1.30, 1.99)	1.00 (0.96, 1.03)	0.835	1.000
Plasma TNF, pg/mL - median (CI)	451 (96.6)	6.73 (6.26, 7.81)	6.73 (5.96, 7.81)	1.00 (0.99, 1.01)	0.608	1.000
Plasma IFN-γ, pg/mL - median (CI)	451 (96.6)	1.14 (1.02, 1.30)	1.14 (1.02, 1.34)	1.01 (0.99, 1.02)	0.419	1.000
Plasma Eotaxin-1/CCL11, pg/mL - median (CI)	451 (96.6)	14.1 (13.6, 14.9)	14.1 (13.6, 15.0)	1.00 (0.99, 1.01)	0.772	1.000
Plasma MIP-1α, pg/mL - median (CI)	451 (96.6)	0.77 (0.77, 0.82)	0.770 (0.720, 0.820)	1.07 (0.91, 1.25)	0.426	1.000
Plasma MIP-1β, pg/mL - median (CI)	451 (96.6)	24.9 (22.4, 26.7)	24.6 (21.6, 27.8)	1.00 (1.00, 1.00)	0.806	1.000
Plasma GM-CSF, pg/mL - median (CI)	451 (96.6)	0.11 (0.03, 0.11)	0.110 (0.029, 0.110)	1.02 (0.98, 1.05)	0.359	1.000
Plasma bFGF, pg/mL - median (CI)	450 (96.3)	2.40 (2.30, 3.14)	2.30 (2.30, 3.40)	1.00 (0.99, 1.01)	0.819	1.000
Plasma C3bc, ng/mL - median (CI)	450 (96.3)	3.64 (3.42, 3.82)	3.67 (3.41, 3.84)	1.00 (0.96, 1.04)	0.839	1.000
**Autonomic markers**						
LF-RRI[i], ms^2 – median (CI)	463 (99.1)	642 (582, 744)	707 (610, 821)	1.09 (1.02, 1.16)	0.017	1.000
HF-RRI[i], ms^2 – median (CI)	463 (99.1)	809.96 (718.0, 923.15)	825 (673, 998)	1.01 (0.95, 1.07)	0.701	1.000
**Cognitive function tests**						
Digit span[k], total score – median (CI)	464 (99.4)	15.12 (14.80, 15.44)	15.3 (14.9, 15.8)	1.02 (1.00, 1.04)	0.062	1.000
Immediate recall[l], score 0 to 36 – median (CI)	464 (99.4)	24.59 (24.22, 24.97)	24.9 (24.4, 25.3)	1.02 (1.00, 1.03)	0.039	1.000
Delayed recall[l], score 0 to 12 – median (CI)	464 (99.4)	8.68 (8.50, 8.86)	8.81 (8.58, 9.03)	1.03 (1.00, 1.07)	0.053	1.000
Recognition index[m], score 0 to 12 – median (CI)	463 (99.1)	12 (11, 12)	12 (11, 12)	1.05 (0.98, 1.12)	0.209	1.000
**Clinical symptoms**						
Fatigue[n], score 0 to 33 – mean (CI)	451 (96.6)	15.61 (15.09, 16.14)	15.5 (14.9, 16.1)	1.00 (0.99, 1.01)	0.466	1.000
Post-exertional malaise[o], score 0 to 100 – median (CI)	451 (96.6)	20 (15, 20)	15.0 (10.0, 20.0)	1.00 (1.00, 1.00)	0.76	1.000
Sleep problems[p], score 1 to 6 – mean (CI)	451 (96.6)	4.01 (3.90, 4.11)	4.03 (3.90, 4.15)	1.01 (0.96, 1.07)	0.629	1.000
Pain[q], score 1 to 10 – median (CI)	451 (96.6)	2.25 (2.00, 2.50)	2.25 (2.00, 2.50)	0.98 (0.94, 1.03)	0.514	1.000
Cognitive symptoms[r], score 3 to 15 – median (CI)	451 (96.6)	6 (5, 6)	6 (5, 6)	0.98 (0.96, 1.00)	0.065	1.000
Respiratory symptoms[s], score 2 to 10 – median (CI)	451 (96.6)	4 (4, 4)	4 (3, 4)	0.99 (0.95, 1.02)	0.334	1.000
Autonomic symptoms[t], score 2 to 10 - median (CI)	451 (96.6)	4 (5, 6)	5 (5, 6)	1.00 (0.97, 1.02)	0.754	1.000
Symptoms of anxiety[u], score 0 to 21 – median (CI)	451 (96.6)	6 (5, 6)	6 (5, 6)	1.00 (0.99, 1.02)	0.795	1.000
Symptoms of depression[u], score 0 to 21 – median (CI)	451 (96.6)	3 (3, 4)	3 (3, 4)	0.98 (0.96, 1.00)	0.015	1.000
Negative emotions[v], score 5 to 25 – median (CI)	451 (96.6)	10 (9, 11)	10 (9, 11)	1.00 (0.99, 1.01)	0.916	1.000
Principal Component: Symptom severity[w] – mean (CI)	451 (96.6)	0.060 (-0.087, 0.098)	-0.256 (-0.135, 0.084)	0.97 (0.91, 1.03)	0.324	1.000
**Psychological traits**						
Neuroticism[x], score 0 to 24 – median (CI)	451 (96.6)	6 (5, 7)	6 (5, 7)	1.00 (0.99, 1.01)	0.520	1.000
Emotional awareness[y], score 7 to 35 – median (CI)	451 (96.6)	13 (12, 14)	13 (12, 14)	1.00 (1.00, 1.01)	0.418	1.000
Worrying tendencies[z], score 16 to 80 – mean (CI)	451 (96.6)	45.54 (44.23, 46.84)	45.9 (44.3, 47.5)	1.00 (0.99, 1.00)	0.569	1.000
Body vigilance[aa], score 0 to 40 – mean (CI)	451 (96.6)	11.99 (11.31, 12.68)	12.1 (11.3, 12.9)	1.00 (0.99, 1.01)	0.685	1.000
Principal component: Emotional maladjustment[ab] – mean (CI)	451 (96.6)	0.005 (-0.087, 0.976)	0.002 (-0.110, 0.113)	1.00 (0.94, 1.06)	0.917	1.000
**Social/behavioural markers**						
Average level of physical activity prior to acute infection[ac], score 1 to 10 – mean (CI)	451 (96.6)	6.37 (6.17, 6.58)	6.61 (6.38, 6.83)	1.05 (1.02, 1.08)	0.001	0.078
Socioeconomic level ISEI-08[ad], score 10 to 90 – median (CI)	423 (90.1)	63.33 (60.29, 68.54)	66.4 (62.4, 68.7)	1.00 (1.00, 1.01)	0.007	0.525
Family member with chronic disease[ae] – no. (%)	451 (96.6)	153 (33.9)	103 (33.6)	0.98 (0.86, 1.12)	0.807	1.000
Loneliness[af], score 20-80 – mean (CI)	451 (96.6)	37.98 (36.99, 38.97)	37.4 (36.2, 38.6)	1.00 (0.99, 1.00)	0.122	1.000

© 2023 Selvakumar J et al. *JAMA Network Open.*

Negative life events last 12 months[ag], impact score – median (CI)	451 (96.6)	2 (2, 2)	2 (1, 2)	1.01 (0.99, 1.02)	0.326	1.000
Negative life events prior to last 12 months[ag], impact score – median (CI)	451 (96.6)	0 (0, 1)	0 (0, 2)	1.02 (0.99, 1.04)	0.248	1.000

CI=95% Confidence interval; NA=Not applicable; SARS-CoV-2= Severe acute respiratory syndrome coronavirus 2; BMI=Body mass index; FVC=Forced vital capacity; SpO_2=Peripheral oxygen saturation; NT-pBNP=N-terminal pro-brain natriuretic peptide; NfL=Neurofilament light chain; GFAp=Glial fibrillary acidic protein; hsCRP=high-sensitive assay of C-reactive protein; GDF-15=Growth/differentiation factor 15; IL=Interleukin; TCC=Terminal complement complex; CAU=Complement arbitrary units; RANTES=Regulated on activation, normal T-cell expressed and secreted; MCP=Monocyte chemotactic protein; IP=Interferon gamma-induced protein; RBD= Receptor binding domain; BAU=Binding antibody units; LF-RRI=Low frequency power of heart rate variability; HF-RRI=High-frequency power of heart rate variability; ISEI-08=International Socioeconomic Index 2008. [a]95% Profile likelihood based confidence intervals. [b]Likelihood ratio p-values. [c]Bonferroni-adjusted for test multiplicity. [d]Standardised score calculated according to World Health Organisation 2006 Child Growth Standards for ages 12-19; for participants above this age, reference values for 19-year-olds were used. [e]One or more doses of immunisation against SARS-CoV-2. [f]The Global Lung Function Initiative 2012 reference values were used to calculate predicted values.[4] [g]Square-root-transformed variable was used for regression analysis. [h]Defined as (NxP)/L, where N, P and L represent neutrophil, platelet and lymphocyte counts respectively. [i]Ln-transformed variable was used for regression analyses. [j]Fifth-root-transformed variable was used for regression analyses. [k]From the Wechsler Intelligence Scales for Children revised; higher score implies better short-term memory. [l]From the Hopkins Verbal Learning Test revised (HVLT-R); higher scores imply better immediate and delayed recall of words, respectively. [m]From the HVLT-R; higher score implies better recognition of words. [n]From the Chalder Fatigue Questionnaire; higher score implies more fatigue. [o]From the DePaul Symptom Questionnaire; higher score implies more post-exertional malaise. [p]From the Karolinska Sleep Questionnaire; higher score implies better sleep. [q]From the Brief Pain Inventory, higher score implies more pain. [r]Self-developed, aggregated score for problems with 'memory', 'concentration', and 'decision making'; higher score implies more symptoms. [s]Self-developed, aggregated score for symptoms 'cough' and 'dyspnoea'; higher score implies more symptoms. [t]Self-developed, aggregated score for symptoms 'dizziness', 'cold and pale hands', 'feeling alternately warm and cold'; higher score implies more symptoms. [u]From the anxiety and depression subscales, respectively, of the Hospital Anxiety and Depression Scale; higher scores imply more symptoms. [v]From the Positive and Negative Affect Schedule; higher score implies more negative emotions. [w]The main component extracted by Principal Component Analysis of the 10 clinical symptoms variables, labelled 'symptom severity'. [x]From the NEO-Five-Factor-Inventory-30; higher scores implies more neuroticism. [y]From the Toronto Alexithymia Scale; higher score implies more difficulty identifying feelings. [z]From the Penn State Worry Questionnaire; higher score implies more worrying. [aa]From the Body Vigilance Scale; higher score implies being more attentive to bodily sensations. [ab]The main component extracted by Principal Component Analysis of the four psychological traits variables, labelled 'emotional maladjustment'. [ac]Self-developed; higher score implies more physical activity. [ad]The ISEI-08 score of the parent with the highest score; higher score implies higher socioeconomic status. [ae]Having a sibling or parent affected by chronic disease. [af]From the University of California, Los Angeles, Loneliness Scale; higher score implies more loneliness. [ag]From the Life Event Checklist; higher score implies more negative impact of past life events.

© 2023 Selvakumar J et al. *JAMA Network Open.*

Table S5. Results of Epstein-Barr virus (EBV) serology at baseline and six months follow-up

	At baseline		At six months follow-up	
	SARS-CoV-2 positive (n=394)[a]	*SARS-CoV-2 negative (n=104)*[a]	*SARS-CoV-2 positive (n=377)*[b]	*SARS-CoV-2 negative (n=84)*[b]
EBV VCA IgM positive[c] – no. (%)	21 (5.3)	1 (1)	18 (4.8)	1 (1.2)
EBV VCA IgG positive[d] – no. (%)	283 (71.8)	58 (55.8)	275 (72.9)	49 (58.3)
EBV EBNA IgG positive[e] – no. (%)	268 (68.0)	59 (56.7)	260 (69.0)	48 (57.1)
Heterophile antibodies[f], positive – no. (%)	3 (0.8)	1 (1.0)	2 (0.5)	0 (0)

The interpretation of the results, based on the overall serological pattern for each individual patient, is presented in table S6. SARS-CoV-2= Severe acute respiratory syndrome coronavirus 2; VCA=Viral capsid antigen; IgM=Immunoglobulin M; IgG=Immunoglobulin G; EBNA=Epstein-Barr Nuclear Antigen. [a]At baseline, there were missing values for 10 and on individuals in the SARS-CoV-2 positive and SARS-CoV-2 negative group, respectively. [b]At follow-up, there were missing values for five and one individuals in the SARS-CoV-2 positive and SARS-CoV-2 negative group, respectively. [c]Positive ≥ 20 U/mL in serum. [d]Positive ≥ 20 U/mL in serum. [e]Positive ≥ 20 U/mL in serum. [f]Only performed when the results of the three EBV-specific immunoglobulin tests were inconclusive.

© 2023 Selvakumar J et al. *JAMA Network Open.*

Table S6. Epstein-Barr virus (EBV) infection status of individuals attending six month follow-up.[a]

	Serological pattern	Prevalence N (%)	
		SARS-CoV-2 positive (n=377)[b]	*SARS-CoV-2 negative (n=84)*[b]
Recent EBV-infection at baseline	Positive IgG antibodies (VCA[c] and/or EBNA[d]) and positive heterophile antibodies at baseline	3 (0.8)	1 (1.2)
EBV-infection in observational period	Seroconversion of IgG antibodies (VCA and/or EBNA) and/or heterophile antibodies from baseline to six months	5 (1.3)	2 (2.4)
Prior (not recent) EBV-infection	Positive IgG antibodies (VCA and/or EBNA) and negative heterophile antibodies at baseline and six months, regardless of VCA[e] IgM result	277 (73.4)	51 (60.7)
Early EBV-infection at six months cannot be ruled out	Positive VCA IgM antibodies at six months only, and negative IgG antibodies (VCA and EBNA) and negative heterophile antibodies	2 (0.5)	0 (0.0)
No serological evidence of EBV infection	Negative IgG antibodies and negative heterophile antibodies at both time points, excepting those in the above category	90 (23.9)	30 (35.7)

SARS-CoV-2= Severe acute respiratory syndrome coronavirus 2; VCA=Viral capsid antigen; IgM=Immunoglobulin M; IgG=Immunoglobulin G; EBNA=Epstein Barr Nuclear Antigen. [a]Participants were classified into mutually exclusive categories of probable infection status, based on the serological pattern of tests performed at both baseline and six months. [b]At follow-up, there were missing values for five and one individuals in the SARS-CoV-2 positive and SARS-CoV-2 negative group, respectively. [c]Positive ≥ 20 U/mL in serum. [d]Positive ≥ 20 U/mL in serum. [e]Positive ≥ 20 U/mL in serum.

© 2023 Selvakumar J et al. *JAMA Network Open.*

Table S7. Point prevalence % (confidence intervals)[a] of long COVID and the post-infective fatigue syndrome at six months follow-up, compared to the control group of SARS-CoV-2-negative individuals. Per protocol data.

	SARS-CoV-2-positive group, % (n=379)[b]	*SARS-CoV-2-negative group, % (n=85)*	*Risk difference, % (95 % CI)*
Long COVID[c] (n=224)	48.5 (43.6 to 53.6)	47.1 (36.8 to 57.6)	1.5 (-10.2 to 13.1)
Post-infective fatigue syndrome[d] (n=60)	14.0 (10.8 to 17.9)	8.2 (3.8 to 16.3)	5.7 (-2.0 to 12.0)

CI=Confidence interval. [a]Agresti-Coull and Agresti-Caffo confidence intervals were calculated, respectively, for prevalence and risk difference. [b]Three of the 382 individuals in the SARS-CoV-2-positive group that attended six months follow-up had missing values in questionnaire data precluding classification; hence, they were removed from prevalence analyses. [c]According to the WHO-definition of long COVID[1]. [d]According to the international case definition of PIFS.[25]

© 2023 Selvakumar J et al. *JAMA Network Open.*

Table S8. Point prevalence % (confidence intervals) [a] of long COVID at six months follow-up. Sensitivity analysis removing cases of uncertain classification, individuals with possible EBV-infection at inclusion or during the observational period, individuals vaccinated before baseline and individuals receiving vaccination less than five days prior to the six months assessment.

	SARS-CoV-2 positive (n=335)	*SARS-CoV-2 negative (n=72)*	*Risk difference*
Long COVID (n=191)	46.9 (41.6, 52.2)	47.2 (36.1, 58.6)	-0.4 (-13,0, 12.1)

SARS-CoV-2= Severe acute respiratory syndrome coronavirus 2. [a]Agresti-Coull and Agresti-Caffo confidence intervals were calculated, respectively, for prevalence and risk difference.

© 2023 Selvakumar J et al. *JAMA Network Open.*

Table S9. Point prevalence % (confidence intervals) [a] of long COVID at six months follow-up. Sensitivity analysis removing cases of uncertain classification, individuals with possible EBV-infection at inclusion or during the observational period, individuals vaccinated before baseline, individuals receiving vaccination less than five days prior to the six month assessment, and individuals in the SARS-CoV-2 negative group with general infectious symptoms score[b] ≥ 11 at baseline.

	SARS-CoV-2 positive (n=335)	*SARS-CoV-2 negative (n=63)*	*Risk difference*
Long COVID (n=185)	46.9 (41.6, 52.2)	44.4 (32.8, 56.7)	2.4 (-10.9 , 15.5)

SARS-CoV-2= Severe acute respiratory syndrome coronavirus 2. [a]Agresti-Coull and Agresti-Caffo confidence intervals were calculated, respectively, for prevalence and risk difference. [b]General infectious symptoms score was computed as the sum across five single items (fever/chills, sore throat, headaches, muscle ache and fatigue after exercise), and has a total range from 5 – 25.[63]

© 2023 Selvakumar J et al. *JAMA Network Open.*

Table S10. Point prevalence % (confidence intervals) [a] of post-infective fatigue syndrome (PIFS) at six months follow-up. Sensitivity analysis removing cases of uncertain classification, individuals with possible EBV-infection at inclusion or during the observational period, individuals vaccinated before baseline and individuals receiving vaccination less than five days prior to the six months assessment.

	SARS-CoV-2 positive (n=343)	*SARS-CoV-2 negative (n=77)*	*Risk difference*
Post-infective fatigue syndrome (n=48)	12.2 (9.1, 16.0)	7.8 (3.3, 16.3)	4.5 (-3.6, 10.8)

SARS-CoV-2= Severe acute respiratory syndrome coronavirus 2. [a]Agresti-Coull and Agresti-Caffo confidence intervals were calculated, respectively, for prevalence and risk difference.

© 2023 Selvakumar J et al. *JAMA Network Open.*

Table S11. Point prevalence % (confidence intervals) [a] of post-infective fatigue syndrome (PIFS) at six months follow-up. Sensitivity analysis removing cases of uncertain classification, individuals with possible EBV-infection at inclusion or during the observational period, individuals vaccinated before baseline and individuals receiving vaccination less than five days prior to the six months assessment, and individuals in the SARS-CoV-2-negative group with general infectious symptoms score[b] ≥ 11 at baseline.

	SARS-COV-2 positive (n=343)	*SARS-COV-2 negative (n=66)*	*Risk difference*
Post-infective fatigue syndrome (n=46)	12.2 (9.1, 16.0)	6.1 (1.9, 15.0)	6.2 (-0.2, 12.2)

SARS-CoV-2= Severe acute respiratory syndrome coronavirus 2. [a]Agresti-Coull and Agresti-Caffo confidence intervals were calculated, respectively, for prevalence and risk difference. [b]General infectious symptoms score was computed as the sum across five single items (fever/chills, sore throat, headaches, muscle ache and fatigue after exercise), and has a total range from 5 – 25.[63]

© 2023 Selvakumar J et al. *JAMA Network Open.*

Table S12. Point prevalence % (confidence intervals)[a] of specific symptoms at baseline and six months follow-up.

	At baseline			At six months follow-up		
	SARS-CoV-2 positive (n=389)[b]	*SARS-CoV-2 negative (n=104)[b]*	*Risk difference*	*SARS-CoV-2 positive (n=379)[c]*	*SARS-CoV-2 negative (n=85)[c]*	*Risk difference*
**SYMPTOMS[d]**						
**Fatigue and post-exertional malaise**						
Fatigue[e]	57.3 (52.4, 62.1)	43.3 (34.2, 52.9)	12.3 (0.4, 23.8)	40.4 (35.5, 45.4)	31.8 (22.8, 42.3)	8.6 (-2.7, 19.2)
Extraordinary fatigue after physical activity	43.7 (38.9, 48.7)	16.3 (10.4, 24.7)	28.1 (18.1, 36.7)	25.9 (21.7, 30.5)	10.6 (5.5, 19.1)	15.3 (6.5, 22.5)
Lack of muscle strength even after resting	27.2 (23.1, 31.9)	17.3 (11.1, 25.8)	10.9 (1.0, 19.4)	21.4 (17.5, 25.8)	11.8 (6.3, 20.5)	9.6 (0.8, 17.0)
Muscle soreness after normal daily activities	26.0 (21.9, 30.5)	15.4 (9.6, 23.6)	8.9 (-0.9, 17.5)	19.3 (15.6, 23.5)	9.4 (4.6, 17.7)	9.8 (1.5, 16.6)
Tired 'in the head' after minimal exertions	35.0 (30.4, 39.8)	21.2 (14.3, 30.0)	11.1 (0.2, 20.8)	21.4 (17.5, 25.8)	18.8 (11.8, 28.5)	2.5 (-7.3, 11.3)
'Empty batteries' after light activities	37.5 (32.9, 42.4)	16.3 (10.4, 24.7)	20.9 (10.8, 29.6)	25.6 (21.5, 30.2)	17.6 (10.9, 27.2)	7.9 (-1.9, 16.6)
Fatigue the day after exertion	41.6 (36.9, 46.6)	32.7 (24.4, 42.2)	9.8 (-1.7, 20.6)	34.6 (30.0, 39.5)	32.9 (23.9, 43.5)	1.6 (-9.7, 12.3)
Unrefreshing sleep	50.6 (45.7, 55.6)	47.1 (37.8, 56.6)	3.3 (-8.4, 15.0)	48.0 (43.0, 53.0)	47.1 (36.8, 57.6)	1.0 (-10.7, 12.5)
**General infectious symptoms**						
Feeling of fever/chills	18.8 (15.2, 23.0)	6.7 (3.1, 13.5)	11.7 (3.8, 17.9)	8.4 (6.0, 11.7)	5.9 (2.2, 13.4)	2.6 (-4.3, 7.8)
Tender lymphatic nodes	8.5 (6.1, 11.7)	2.9 (0.6, 8.5)	5.2 (-0.4, 9.1)	5.3 (3.4, 8.1)	5.9 (2.2, 13.4)	-0.6 (-7.2, 4.4)
Muscles pain	31.1 (26.7, 35.9)	22.1 (15.2, 31.1)	11.7 (1.5, 20.8)	17.9 (14.4, 22.1)	15.3 (9.0, 24.6)	2.6 (-6.6, 10.7)
Multi-joint pain	19.8 (16.1, 24.1)	9.6 (5.1, 17.0)	10.1 (1.7, 17.0)	15.0 (11.8, 19.0)	10.6 (5.5, 19.1)	4.5 (-3.9, 11.3)
Headache	48.8 (43.9, 53.8)	35.6 (27.0, 45.2)	11.3 (-0.4, 22.4)	32.5 (27.9, 37.3)	31.8 (22.8, 42.3)	0.7 (-10.5, 11.2)
**Cognitive symptoms**						
Memory problems	26.0 (21.9, 30.5)	27.9 (20.1, 37.2)	-7.7 (-18.9, 3.0)	36.4 (31.7, 41.4)	29.4 (20.7, 39.9)	7.0 (-4.2, 17.4)
Concentration problems	50.4 (45.4, 55.3)	51.0 (41.5, 60.4)	-2.2 (-13.9, 9.5)	48.3 (43.3, 53.3)	44.7 (34.6, 55.3)	3.6 (-8.1, 15.1)
Problems making decisions	26.0 (21.9, 30.5)	34.6 (26.2, 44.2)	-8.4 (-19.6, 2.4)	33.0 (28.4, 37.9)	23.5 (15.7, 33.6)	9.5 (-1.2, 19.1)
**Respiratory symptoms**						
Shortness of breath/dyspnea	31.4 (26.9, 36.1)	9.6 (5.1, 17.0)	21.8 (13.0, 29.0)	20.1 (16.3, 24.4)	11.8 (6.3, 20.5)	8.3 (-0.5, 15.6)
Cough	46.3 (41.4, 51.2)	17.3 (11.1, 25.8)	27.0 (16.5, 36.1)	22.2 (18.3, 26.6)	21.2 (13.8, 31.1)	1.0 (-9.2, 10.1)
**ENT symptoms**						
Altered smell[f]	NA	NA	NA	25.9 (21.7, 30.5)	0.0 (0.0, 5.2)	25.9 (19.9, 29.8)
Altered taste[f]	NA	NA	NA	17.9 (14.4, 22.1)	0.0 (0.0, 5.2)	17.9 (12.5, 21.4)
Sore throat	26.5 (22.3, 31.1)	10.6 (5.9, 18.1)	15.4 (6.5, 22.8)	12.9 (9.9, 16.7)	17.6 (10.9, 27.2)	-4.7 (-14.1, 3.6)
**Cardiac symptoms**						
Chest pain	15.2 (11.9, 19.1)	10.6 (5.9, 18.1)	1.6 (-7.2, 9.1)	12.1 (9.2, 15.8)	4.7 (1.5, 11.9)	7.4 (0.7, 12.5)
Palpitations	14.1 (11.0, 18.0)	10.6 (5.9, 18.1)	1.7 (-6.9, 8.9)	13.5 (10.4, 17.3)	7.1 (3.0, 14.8)	6.4 (-1.1, 12.3)
**Autonomic symptoms**						
Dizziness	39.1 (34.4, 44.0)	32.7 (24.4, 42.2)	3.9 (-7.6, 14.8)	31.4 (26.9, 36.2)	31.8 (22.8, 42.3)	-0.4 (-11.6, 10.2)
Pale and cold hands	27.5 (23.3, 32.2)	20.2 (13.5, 29.0)	5.5 (-4.9, 14.9)	20.1 (16.3, 24.4)	25.9 (17.7, 36.1)	-5.8 (-16.3, 3.9)
Felt alternately hot and cold	28.5 (24.3, 33.2)	15.4 (9.6, 23.6)	12.9 (3.1, 21.2)	21.1 (17.3, 25.5)	17.6 (10.9, 27.2)	3.5 (-6.2, 12.0)

SARS-CoV-2=Severe acute respiratory syndrome coronavirus 2; NA=Not applicable. ENT=Ear-nose-throat. [a]Agresti-Coull and Agresti-Caffo confidence intervals were calculated, respectively, for prevalence and risk difference. [b]Sixteen out of 509 cases had missing data in the symptom questionnaire at baseline, and are thus not included in the prevalence analysis. [c]Three out of 467 cases had missing data in the symptom questionnaire at six months, and are thus not included in the prevalence analysis. [d]With the exception of 'fatigue', all symptoms were self-reported on a Likert scale 1-5, with 1 corresponding to 'Never' and 5 to 'Constantly'. The prevalence presented is for reporting a value of three or higher. [e]From the Chalder Fatique Questionnaire; prevalence reported is for a total score of 4 or higher, using the bimodal scoring method. [f]Altered smell/taste were not included in the questionnaire at baseline.

© 2023 Selvakumar J et al. *JAMA Network Open.*

Table S13. Results of final factor analyses (Principal Component Analysis) of ten clinical symptoms variables and four psychological traits variables, respectively. Per protocol data.

	**Principal component from clinical symptoms variables: 'Symptom severity'**	**Principal component from psychological traits variables: 'Emotional maladjustment'**
Total variance explained (%)	52.5	66.3
Bartlett's test of sphericity (p-value)	<0.001	<0.001
Kaiser-Meyer-Olkin measure of sampling adequacy	0.911	0.742
**Loading variables**		
Fatigue[a] (factor loading)	0.818	
Post-exertional malaise[b] (factor loading)	0.816	
Sleep problems[c] (factor loading)	-0.817	
Pain[d] (factor loading)	0.612	
Cognitive symptoms[e] (factor loading)	0.769	
Respiratory symptoms[f] (factor loading)	0.537	
Autonomic symptoms[g] (factor loading)	0.760	
Symptoms of anxiety[h] (factor loading)	0.748	
Symptoms of depression[h] (factor loading)	0.677	
Negative emotions[i] (factor loading)	0.633	
Neuroticism[j] (factor loading)		0.903
Emotional awareness[k] (factor loading)		0.833
Worrying tendencies[l] (factor loading)		0.860
Body vigilance[m] (factor loading)		0.637

[a]From the Chalder Fatigue Questionnaire; higher score implies more fatigue. [b]From the DePaul Symptom Questionnaire; higher score implies more post-exertional malaise. [c]From the Karolinska Sleep Questionnaire; higher score implies better sleep. [d]From the Brief Pain Inventory, higher score implies more pain. [e]Self-developed, aggregated score for problems with 'memory', 'concentration', and 'decision making'; higher score implies more symptoms. [f]Self-developed, aggregated score for symptoms 'cough' and 'dyspnoea'; higher score implies more symptoms. [g]Self-developed, aggregated score for symptoms 'dizziness', 'cold and pale hands', 'feeling alternately warm and cold'; higher score implies more symptoms. [h]From the anxiety and depression subscales, respectively, of the Hospital Anxiety and Depression Scale; higher scores imply more symptoms. [i]From the Positive and Negative Affect Schedule; higher score implies more negative emotions. [j]From the NEO-Five-Factor-Inventory-30; higher scores implies more neuroticism. [k]From the Toronto Alexithymia Scale; higher score implies more difficulty identifying feelings. [l]From the Penn State Worry Questionnaire; higher score implies more worrying. [m]From the Body Vigilance Scale; higher score implies being more attentive to bodily sensations.

© 2023 Selvakumar J et al. *JAMA Network Open.*

Table S14. Results of final factor analyses (Principal Component Analysis) of ten clinical symptoms variables and four psychological traits variables, respectively. Sensitivity analysis removing cases of uncertain classification, individuals with possible EBV-infection at inclusion or during the observational period, individuals vaccinated before baseline and individuals receiving vaccination less than five days prior to the six months assessment.

	**Principal components for analysis of the long COVID condition (n=410)**		**Principal component for analysis of post-infective fatigue syndrome (n=423)**	
	Principal component from clinical symptoms variables: 'Symptom severity'	*Principal component from psychological traits variables: 'Emotional maladjustment'*	*Principal component from clinical symptoms variables: 'Symptom severity'*	*Principal component from psychological traits variables: 'Emotional maladjustment'*
Total variance explained (%)	51.4	65.6	51.3	65.5
Bartlett's test of sphericity (p-value)	<0.001	<0.001	<0.001	<0.001
Kaiser-Meyer-Olkin measure of sampling adequacy	0.908	0.737	0.907	0.734
**Loading variables**				
Fatigue[a] (factor loading)	0.819		0.817	
Post-exertional malaise[b] (factor loading)	0.812		0.811	
Sleep problems[c] (factor loading)	-0.808		-0.817	
Pain[d] (factor loading)	0.577		0.574	
Cognitive symptoms[e] (factor loading)	0.766		0.771	
Respiratory symptoms[f] (factor loading)	0.549		0.533	
Autonomic symptoms[g] (factor loading)	0.760		0.753	
Symptoms of anxiety[h] (factor loading)	0.740		0.746	
Symptoms of depression[h] (factor loading)	0.649		0.649	
Negative emotions[i] (factor loading)	0.622		0.623	
Neuroticism[j] (factor loading)		0.901		0.901
Emotional awareness[k] (factor loading)		0.826		0.828
Worrying tendencies[l] (factor loading)		0.854		0.859
Body vigilance[m] (factor loading)		0.633		0.619

[a]From the Chalder Fatigue Questionnaire; higher score implies more fatigue. [b]From the DePaul Symptom Questionnaire; higher score implies more post-exertional malaise. [c]From the Karolinska Sleep Questionnaire; higher score implies better sleep. [d]From the Brief Pain Inventory, higher score implies more pain. [e]Self-developed, aggregated score for problems with 'memory', 'concentration', and 'decision making'; higher score implies more symptoms. [f]Self-developed, aggregated score for symptoms 'cough' and 'dyspnoea'; higher score implies more symptoms. [g]Self-developed, aggregated score for symptoms 'dizziness', 'cold and pale hands', 'feeling alternately warm and cold'; higher score implies more symptoms. [h]From the anxiety and depression subscales, respectively, of the Hospital Anxiety and Depression Scale; higher scores imply more symptoms. [i]From the Positive and Negative Affect Schedule; higher score implies more negative emotions. [j]From the NEO-Five-Factor-Inventory-30; higher scores implies more neuroticism. [k]From the Toronto Alexithymia Scale; higher score implies more difficulty identifying feelings. [l]From the Penn State Worry Questionnaire; higher score implies more worrying. [m]From the Body Vigilance Scale; higher score implies being more attentive to bodily sensations.

© 2023 Selvakumar J et al. *JAMA Network Open.*

Table S15. Results of final factor analyses (Principal Component Analysis) of ten clinical symptoms variables and four psychological traits variables, respectively. Sensitivity analysis featuring imputation of mean/median for missing data.

	**Principal component from clinical symptoms variables: 'Symptom severity'**	**Principal component from psychological traits variables: 'Emotional maladjustment'**
Total variance explained (%)	52.5	66.3
Bartlett's test of sphericity (p-value)	<0.001	<0.001
Kaiser-Meyer-Olkin measure of sampling adequacy	0.911	0.741
**Loading variables**		
Fatigue[a] (factor loading)	0.816	
Post-exertional malaise[b] (factor loading)	0.816	
Sleep problems[c] (factor loading)	-0.817	
Pain[d] (factor loading)	0.613	
Cognitive symptoms[e] (factor loading)	0.769	
Respiratory symptoms[f] (factor loading)	0.538	
Autonomic symptoms[g] (factor loading)	0.760	
Symptoms of anxiety[h] (factor loading)	0.748	
Symptoms of depression[h] (factor loading)	0.677	
Negative emotions[i] (factor loading)	0.633	
Neuroticism[j] (factor loading)		0.903
Emotional awareness[k] (factor loading)		0.833
Worrying tendencies[l] (factor loading)		0.859
Body vigilance[m] (factor loading)		0.636

[a]From the Chalder Fatigue Questionnaire; higher score implies more fatigue. [b]From the DePaul Symptom Questionnaire; higher score implies more post-exertional malaise. [c]From the Karolinska Sleep Questionnaire; higher score implies better sleep. [d]From the Brief Pain Inventory, higher score implies more pain. [e]Self-developed, aggregated score for problems with 'memory', 'concentration', and 'decision making'; higher score implies more symptoms. [f]Self-developed, aggregated score for symptoms 'cough' and 'dyspnoea'; higher score implies more symptoms. [g]Self-developed, aggregated score for symptoms 'dizziness', 'cold and pale hands', 'feeling alternately warm and cold'; higher score implies more symptoms. [h]From the anxiety and depression subscales, respectively, of the Hospital Anxiety and Depression Scale; higher scores imply more symptoms. [i]From the Positive and Negative Affect Schedule; higher score implies more negative emotions. [j]From the NEO-Five-Factor-Inventory-30; higher scores implies more neuroticism. [k]From the Toronto Alexithymia Scale; higher score implies more difficulty identifying feelings. [l]From the Penn State Worry Questionnaire; higher score implies more worrying. [m]From the Body Vigilance Scale; higher score implies being more attentive to bodily sensations.

© 2023 Selvakumar J et al. *JAMA Network Open.*

Table S16. Characteristics of potential baseline risk factors and their univariate associations (Poisson regression with log-link and robust error variances) to long COVID and post-infective fatigue syndrome (PIFS) at six months follow-up. Per protocol data.

	Baseline characteristics		Univariate association to long COVID		Univariate association to PIFS	
	SARS-CoV-2 positive (n=382)	*SARS-CoV-2 negative (n=85)*	*Relative risk (CI)[a]*	*p-value[b]*	*Relative risk (CI)[a]*	*p-value[b]*
**SARS-CoV-2 status**						
SARS-CoV-2-positive at baseline – no. (%)	NA	NA	1.03 (0.81, 1.33)	0.804	1.70 (0.86, 3.85)	0.133
**Background and constitutional factors**						
Female sex – no. (%)	230 (60.2)	54 (63.5)	1.48 (1.21, 1.82)	<0.001	3.66 (1.97, 7.53)	<0.001
Age, years – mean (CI)	17.98 (17.61, 18.35)	17.73 (17.04, 18.43)	1.01 (0.98, 1.03)	0.678	1.08 (1.01, 1.15)	0.031
BMI, z-score[d] – mean (CI)	0.44 (0.32, 0.55)	.48 (0.23, 0.72)	1.01 (0.93, 1.09)	0.858	0.94 (0.76, 1.15)	0.522
Ethnicity non-European – no. (%)	88 (23.0)	2 (2.4)	1.09 (0.86, 1.37)	0.479	1.67 (0.96, 2.77)	0.068
Any comorbidity – no. (%)	79 (21.4)	28 (33.3)	1.34 (1.09, 1.64)	0.007	1.39 (0.81, 2.29)	0.226
**Observational period characteristics**						
Time span between baseline and follow-up, days – median (range)	193 (191, 195)	193 (190, 196)	1.00 (1.00, 1.01)	0.369	1.00 (0.98, 1.01)	0.710
Immunisation against SARS-CoV-2[e] – no. (%)	4 (1.0)	3 (3.5)	0.89 (0.34, 1.83)	0.769	2.25 (0.43, 6.76)	0.285
**Organ function tests/biomarkers**						
FVC, % of predicted[f] – mean (CI)	99.4 (98.3, 100.6)	100.8 (98.4, 103.2)	1.00 (0.99, 1.01)	0.770	0.98 (0.96, 1.01)	0.243
SpO_2, % – mean (CI)	98.67 (98.56, 98.78)	98.57 (98.30, 98.84)	1.06 (0.97, 1.16)	0.198	1.24 (1.00, 1.57)	0.056
NT-pBNP, ng/L – median (CI)	34 (30, 38)	35 (26, 44)	1.00 (1.00, 1.00)	0.421	1.01 (1.00, 1.01)	0.099
Troponin T, ng/L – median (CI)	4.00 (4.00, 4.00)	2.89 (2.21, 4.00)	0.98 (0.95, 1.02)	0.338	0.88 (0.79, 0.97)	0.011
NfL, pg/mL – mean (CI)	4.73 (4.33, 5.12)	4.20 (3.86, 4.54)	0.97 (0.92, 1.01)	0.116	0.96 (0.83, 1.04)	0.456
GFAp, pg/mL – mean (CI)	70.02 (64.56, 75.48)	56.02 (51.09, 60.95)	1.00 (0.99, 1.00)	0.037	1.00 (0.99, 1.00)	0.530
D-dimer[g], mg/L – median (CI)	0.17 (0.15, 0.19)	0.19 (0.17, 0.21)	0.68 (0.39, 1.19)	0.178	1.19 (0.29, 4.44)	0.807
Ferritin, µg/L – median (CI)	69 (64, 76)	48 (42, 60)	1.00 (1.00, 1.00)	0.150	1.00 (0.99, 1.00)	0.620
Vitamin B_{12}, pmol/L – mean (CI)	443.97 (426.58, 461.36)	419.66 (385.34, 453.97)	1.00 (1.00, 1.00)	0.158	0.998 (0.996, 0.999)	0.008
**Immunological markers**						
Blood Leukocyte count, 10^9 cells/L - mean (CI)	18.0 (17.6, 18.4)	17.7 (17.0, 18.4)	1.03 (0.97, 1.10)	0.323	0.96 (0.81, 1.13)	0.665
Blood Lymphocyte count, 10^9 cells/L - mean (CI)	2.1 (2.1, 2.2)	2.1 (1.9, 2.2)	0.97 (0.82, 1.15)	0.753	0.73 (0.45, 1.13)	0.162
Blood Monocyte count, 10^9 cells/L - mean (CI)	0.45 (0.44, 0.47)	0.42 (0.39, 0.45)	1.47 (0.78, 2.72)	0.235	0.53 (0.09, 2.64)	0.448
Blood Neutrophil count, 10^9 cells/L - mean (CI)	3.2 (3.0, 3.3)	3.1 (2.8, 3.3)	1.07 (0.98, 1.16)	0.138	1.07 (0.87, 1.30)	0.528
Neutrophil-to-Lymphocyte ratio – mean (CI)	1.6 (1.5, 1.6)	1.6 (1.4, 1.7)	1.12 (0.97, 1.29)	0.130	1.26 (0.89, 1.74)	0.185
Systemic immune-inflammation index - median (CI)[h]	410.8 (389.1, 432.5)	395.8 (357.9, 433.8)	1.00 (1.00, 1.00)	0.055	1.001 (1.000, 1.002)	0.035
hsCRP[i], mg/L – median (CI)	0.83 (0.73, 1.10)	1.29 (0.74, 1.69)	0.99 (0.91, 1.06)	0.723	1.05 (0.87, 1.26)	0.634
GDF15, ng/mL – mean (CI)	0.41 (0.39, 0.42)	0.40 (0.46, 0.43)	0.98 (0.54, 1.67)	0.929	2.29 (0.68, 5.73)	0.164
TCC/C5b-9, CAU/mL – median (CI)	0.18 (0.16, 0.20)	0.003 (0.002, 0.050)	1.01 (0.93, 1.07)	0.715	1.09 (0.95, 1.17)	0.174
RANTES/CCL5[j], pg/mL – median (CI)	261.07 (234.66, 292.45)	271.49 (221.28, 320.20)	1.02 (0.91, 1.13)	0.725	1.03 (0.78, 1.33)	0.821
MCP-1/CCL2, pg/mL – mean (CI)	12.84 (12.20, 13.47)	13.80 (12.56, 15.04)	1.00 (0.98, 1.01)	0.722	1.01 (0.97, 1.05)	0.594
IP-10, pg/mL – mean (CI)	164.14 (155.32, 172.96)	125.93 (113.88, 137.99)	1.00 (1.00, 1.00)	0.037	1.00 (0.996, 1.00)	0.852
SARS-CoV-2-Anti-RBD, BAU/mL – median (CI)	1046 (983, 1133)	1 (1, 1)	1.00 (1.00, 1.00)	0.363	1.00 (1.00, 1.00)	0.919
Plasma total IgG, g/L - mean (CI)	11.1 (10.8, 11.3)	10.7 (10.3, 11.1)	1.02 (0.97, 1.06)	0.499	1.03 (0.92, 1.15)	0.616
Plasma total IgM, g/L - mean (CI)	1.27 (1.22, 1.33)	1.10 (0.99, 1.22)	1.00 (0.83, 1.20)	0.998	0.89 (0.54, 1.39)	0.614
Plasma total IgA, g/L - mean (CI)	1.71 (1.63, 1.78)	1.58 (1.42, 1.74)	1.06 (0.93, 1.20)	0.372	0.88 (0.63, 1.21)	0.454
Plasma IL-1β, pg/mL – median (CI)	0.63 (0.47, 0.73)	0.01 (0.01, 0.19)	0.94 (0.82, 1.05)	0.274	1.06 (0.79, 1.35)	0.656
Plasma IL-2, pg/mL - median (CI)	0.69 (0.47, 1.09)	0.40 (0.03, .78)	0.99 (0.92, 1.05)	0.741	1.08 (0.92, 1.23)	0.341
Plasma IL-4, pg/mL - median (CI)	1.46 (1.39, 1.50)	0.88 (0.75, 0.92)	0.98 (0.87, 1.11)	0.798	1.04 (0.76, 1.39)	0.803
Plasma IL-7, pg/mL - median (CI)	12.6 (11.5, 12.6)	2.98 (1.79, 5.65)	1.00 (0.99, 1.01)	0.636	0.98 (0.96, 1.01)	0.171
Plasma IL-8, pg/mL - median (CI)	0.80 (0.58, 1.08)	0.098 (0.077, 0.12)	1.00 (0.95, 1.03)	0.809	1.07 (0.99, 1.12)	0.094
Plasma IL-9, pg/mL - median (CI)	68.2 (60.5, 80.7)	70.2 (51.5, 86.4)	1.00 (1.00, 1.00)	0.561	1.00 (1.00, 1.00)	0.785
Plasma IL-12, pg/mL - median (CI)	1.49 (1.38, 1.50)	0.194 (0.138, 1.05)	1.00 (0.97, 1.02)	0.789	1.01 (0.96, 1.06)	0.573

© 2023 Selvakumar J et al. *JAMA Network Open.*

Plasma IL-13, pg/mL - median (CI)	0.26 (0.25, 0.27)	0.51 (0.45, 0.66)	0.98 (0.89, 1.05)	0.567	0.96 (0.72, 1.14)	0.692
Plasma IL-17A, pg/mL - median (CI)	1.62 (1.55, 1.99)	1.35 (0.69, 2.03)	1.01 (0.96, 1.06)	0.812	1.07 (0.95, 1.20)	0.252
Plasma TNF, pg/mL - median (CI)	7.81 (6.73, 8.24)	4.26 (3.04, 5.40)	0.99 (0.98, 1.01)	0.397	0.99 (0.94, 1.02)	0.427
Plasma IFN-γ, pg/mL - median (CI)	1.30 (1.02, 1.34)	0.94 (0.94, 1.14)	1.00 (0.97, 1.02)	0.997	1.03 (0.98, 1.06)	0.227
Plasma Eotaxin-1/CCL11, pg/mL - median (CI)	14.8 (14.0, 15.2)	12.7 (11.6, 14.0)	1.00 (0.98, 1.01)	0.601	1.00 (0.97, 1.04)	0.843
Plasma MIP-1α, pg/mL - median (CI)	0.77 (0.67, 0.82)	0.79 (0.79, 1.02)	1.10 (0.86, 1.40)	0.455	0.95 (0.50, 1.73)	0.858
Plasma MIP-1β, pg/mL - median (CI)	24.9 (22.5, 27.3)	25.2 (19.4, 30.0)	1.00 (1.00, 1.00)	0.545	1.00 (0.99, 1.01)	0.883
Plasma GM-CSF, pg/mL - median (CI)	0.20 (0.11, 0.34)	0.017 (0.014, 0.023)	1.01 (0.96, 1.06)	0.631	1.05 (0.92, 1.15)	0.399
Plasma bFGF, pg/mL - median (CI)	3.40 (2.72, 3.40)	1.32 (1.08, 1.53)	1.01 (0.99, 1.02)	0.491	1.02 (0.99, 1.05)	0.127
Plasma C3bc, ng/mL - median (CI)	3.83 (3.67, 4.11)	2.92 (2.70, 3.15)	0.99 (0.93, 1.05)	0.705	0.96 (0.82, 1.11)	0.605
**Autonomic markers**						
LF-RRI[i], ms^2 – median (CI)	654 (585, 746)	585 (467, 841)	0.99 (0.90, 1.10)	0.865	0.78 (0.61, 1.00)	0.046
HF-RRI[i], ms^2 – median (CI)	784 (682, 903)	1006 (724, 1253)	0.99 (0.91, 1.08)	0.796	0.92 (0.74, 1.13)	0.420
**Cognitive function tests**						
Digit span[k], total score – median (CI)	15.15 (14.79, 15.51)	14.97 (14.27, 15.68)	1.00 (0.97, 1.02)	0.730	1.02 (0.96, 1.09)	0.491
Immediate recall[l], score 0 to 36 – median (CI)	24.60 (24.17, 25.02)	24.58 (23.77, 25.39)	0.98 (0.96, 1.01)	0.168	1.02 (0.97, 1.08)	0.460
Delayed recall[l], score 0 to 12 – median (CI)	8.73 (8.52, 8.94)	8.45 (8.06, 8.84)	1.01 (0.96, 1.05)	0.845	1.07 (0.95, 1.21)	0.279
Recognition index[m], score 0 to 12 – median (CI)	12 (11, 12)	12 (11, 12)	1.08 (0.98, 1.21)	0.128	1.24 (0.95, 1.68)	0.128
**Clinical symptoms**						
Fatigue[n], score 0 to 33 – mean (CI)	16.15 (15.57, 16.74)	13.26 (12.22, 14.31)	1.06 (1.05, 1.08)	<0.001	1.22 (1.18, 1.26)	<0.001
Post-exertional malaise[o], score 0 to 100 – median (CI)	20 (15, 25)	10 (10, 15)	1.01 (1.01, 1.02)	<0.001	1.04 (1.03, 1.04)	<0.001
Sleep problems[p], score 1 to 6 – mean (CI)	4.05 (3.93, 4.17)	3.83 (3.64, 4.02)	0.69 (0.64, 0.75)	<0.001	0.37 (0.30, 0.44)	<0.001
Pain[q], score 1 to 10 – median (CI)	2.25 (2.00, 2.50)	2.50 (2.00, 2.75)	1.24 (1.16, 1.32)	<0.001	1.65 (1.43, 1.91)	<0.001
Cognitive symptoms[r], score 3 to 15 – median (CI)	6 (5, 6)	6 (5, 8)	1.12 (1.09, 1.15)	<0.001	1.30 (1.22, 1.38)	<0.001
Respiratory symptoms[s], score 2 to 10 – median (CI)	4 (4, 5)	3 (3, 3)	1.12 (1.07, 1.16)	<0.001	1.28 (1.16, 1.42)	<0.001
Autonomic symptoms[t], score 2 to 10 - median (CI)	6 (5, 6)	5 (5, 6)	1.11 (1.07, 1.14)	<0.001	1.33 (1.24, 1.41)	<0.001
Symptoms of anxiety[u], score 0 to 21 – median (CI)	5 (5, 6)	7 (6, 8)	1.09 (1.06, 1.11)	<0.001	1.19 (1.13, 1.25)	<0.001
Symptoms of depression[u], score 0 to 21 – median (CI)	3 (3, 4)	3 (3, 5)	1.10 (1.07, 1.12)	<0.001	1.21 (1.15, 1.27)	<0.001
Negative emotions[v], score 5 to 25 – median (CI)	9 (9, 10)	11 (10, 13)	1.06 (1.04, 1.07)	<0.001	1.13 (1.08, 1.17)	<0.001
Principal Component: Symptom severity[w] – mean (CI)	NA	NA	1.54 (1.41, 1.67)	<0.001	3.04 (2.54, 3.67)	<0.001
**Psychological traits**				<0.001		<0.001
Neuroticism[x], score 0 to 24 – median (CI)	6 (5, 7)	7 (5, 11)	1.06 (1.05, 1.08)	<0.001	1.12 (1.08, 1.16)	<0.001
Emotional awareness[y], score 7 to 35 – median (CI)	13 (12, 14)	14.5 (12, 17)	1.06 (1.04, 1.07)	<0.001	1.11 (1.07, 1.15)	<0.001
Worrying tendencies[z], score 16 to 80 – mean (CI)	45.03 (43.58, 46.48)	47.74 (47.64, 47.50)	1.02 (1.02, 1.03)	<0.001	1.05 (1.04, 1.07)	<0.001
Body vigilance[aa], score 0 to 40 – mean (CI)	12.01 (11.24, 12.79)	11.90 (11.72, 10.83)	1.03 (1.01, 1.04)	<0.001	1.06 (1.03, 1.09)	<0.001
Principal component: Emotional maladjustment[ab] – mean (CI)	NA	NA	1.48 (1.35, 1.61)	<0.001	2.21 (1.79, 2.75)	<0.001
**Social/behavioural markers**						
Average level of physical activity prior to acute infection[ac], score 1 to 10 – mean (CI)	6.42 (6.20, 6.65)	6.17 (5.72, 6.61)	0.92 (0.88, 0.96)	<0.001	0.87 (0.78, 0.96)	0.007
Socioeconomic level ISEI-08[ad], score 10 to 90 – median (CI)	63.88 (58.77, 68.54)	63.03 (58.77, 68.70)	1.00 (0.990, 1.000)	0.051	0.996 (0.984, 1.008)	0.481
Family member with chronic disease[ae] – no. (%)	123 (33.5)	30 (35.7)	1.33 (1.10, 1.61)	0.004	1.82 (1.13, 2.93)	0.014
Loneliness[af], score 20-80 – mean (CI)	37.65 (36.57, 38.73)	39.39 (36.94, 41.85)	1.03 (1.02, 1.04)	<0.001	1.06 (1.04, 1.08)	<0.001
Negative life events last 12 months[ag], impact score – median (CI)	2 (1, 2)	2 (2, 3)	1.05 (1.03, 1.07)	<0.001	1.09 (1.04, 1.13)	<0.001
Negative life events prior to last 12 months[ag], impact score – median (CI)	0 (0, 1)	2 (0, 3)	1.05 (1.01, 1.09)	0.023	0.998 (0.89, 1.10)	0.971

© 2023 Selvakumar J et al. *JAMA Network Open.*

CI=95% Confidence interval; NA=Not applicable; SARS-CoV-2= Severe acute respiratory syndrome coronavirus 2; BMI=Body mass index; FVC=Forced vital capacity; SpO_2=Peripheral oxygen saturation; NT-pBNP=N-terminal pro-brain natriuretic peptide; NfL=Neurofilament light chain; GFAp=Glial fibrillary acidic protein; hsCRP=high-sensitive assay of C-reactive protein; GDF-15=Growth/differentiation factor 15; IL=Interleukin; TCC=Terminal complement complex; CAU=Complement arbitrary units; RANTES=Regulated on activation, normal T-cell expressed and secreted; MCP=Monocyte chemotactic protein; IP=Interferon gamma-induced protein; RBD= Receptor binding domain; BAU=Binding antibody units; LF-RRI=Low frequency power of heart rate variability; HF-RRI=High-frequency power of heart rate variability; ISEI-08=International Socioeconomic Index 2008. [a]95% Profile likelihood based confidence intervals. [b]Likelihood ratio p-values. [c]Bonferroni-adjusted for test multiplicity. [d]Standardised score calculated according to World Health Organisation 2006 Child Growth Standards for ages 12-19; for participants above this age, reference values for 19-year-olds were used. [e]One or more doses of immunisation against SARS-CoV-2. [f]The Global Lung Function Initiative 2012 reference values were used to calculate predicted values.[4] [g]Square-root-transformed variable was used for regression analysis. [h]Defined as (NxP)/L, where N, P and L represent neutrophil, platelet and lymphocyte counts respectively. [i]Ln-transformed variable was used for regression analyses. [j]Fifth-root-transformed variable was used for regression analyses. [k]From the Wechsler Intelligence Scales for Children revised; higher score implies better short-term memory. [l]From the Hopkins Verbal Learning Test revised (HVLT-R); higher scores imply better immediate and delayed recall of words, respectively. [m]From the HVLT-R; higher score implies better recognition of words. [n]From the Chalder Fatigue Questionnaire; higher score implies more fatigue. [o]From the DePaul Symptom Questionnaire; higher score implies more post-exertional malaise. [p]From the Karolinska Sleep Questionnaire; higher score implies better sleep. [q]From the Brief Pain Inventory, higher score implies more pain. [r]Self-developed, aggregated score for problems with ‘memory’, ‘concentration’, and ‘decision making’; higher score implies more symptoms. [s]Self-developed, aggregated score for symptoms ‘cough’ and ‘dyspnoea’; higher score implies more symptoms. [t]Self-developed, aggregated score for symptoms ‘dizziness’, ‘cold and pale hands’, ‘feeling alternately warm and cold’; higher score implies more symptoms. [u]From the anxiety and depression subscales, respectively, of the Hospital Anxiety and Depression Scale; higher scores imply more symptoms. [v]From the Positive and Negative Affect Schedule; higher score implies more negative emotions. [w]The main component extracted by Principal Component Analysis of the 10 clinical symptoms variables, labelled ‘symptom severity’. [x]From the NEO-Five-Factor-Inventory-30; higher scores implies more neuroticism. [y]From the Toronto Alexithymia Scale; higher score implies more difficulty identifying feelings. [z]From the Penn State Worry Questionnaire; higher score implies more worrying. [aa]From the Body Vigilance Scale; higher score implies being more attentive to bodily sensations. [ab]The main component extracted by Principal Component Analysis of the four psychological traits variables, labelled ‘emotional maladjustment’. [ac]Self-developed; higher score implies more physical activity. [ad]The ISEI-08 score of the parent with the highest score; higher score implies higher socioeconomic status. [ae]Having a sibling or parent affected by chronic disease. [af]From the University of California, Los Angeles, Loneliness Scale; higher score implies more loneliness. [ag]From the Life Event Checklist; higher score implies more negative impact of past life events.

© 2023 Selvakumar J et al. *JAMA Network Open.*

Table S17. Potential baseline risk factors and their univariate associations (Poission regression with log-link) to long COVID and post-infective fatigue syndrome (PIFS) at six months follow-up. Sensitivity analysis removing cases of uncertain classification, individuals with possible EBV-infection at inclusion or during the observational period, individuals vaccinated before baseline and individuals receiving vaccination less than five days prior to the six months assessment.

	**Univariate association to long COVID (n=410)**		**Univariate association to PIFS (n=423)**	
	Relative risk (CI)[a]	*p-value*[b]	*Relative risk (CI)*[a]	*p-value*[b]
**SARS-CoV-2 status**				
SARS-CoV-2-positive at baseline	0.99 (0.76, 1.31)	0.956	1.57 (0.75, 3.88)	0.246
**Background and constitutional factors**				
Female sex	1.55 (1.24, 1.94)	<0.001	3.47 (1.78, 7.58)	<0.001
Age, years	1.01 (0.98, 1.04)	0.635	1.07 (0.99, 1.15)	0.081
BMI, z-score[d]	0.98 (0.90, 1.08)	0.709	0.94 (0.74, 1.18)	0.581
Ethnicity non-European	1.01 (0.77, 1.31)	0.932	1.17 (0.58, 2.18)	0.641
Any comorbidity	1.36 (1.08, 1.71)	0.010	1.64 (0.91, 2.84)	0.099
**Observational period characteristics**				
Time span between baseline and follow-up, days	1.00 (1.00, 1.01)	0.586	0.99 (0.97, 1.01)	0.331
Immunisation against SARS-CoV-2[e]	NA	NA	NA	NA
**Organ function tests/biomarkers**				
FVC, % of predicted[f]	1.00 (0.99, 1.01)	0.574	0.98 (0.95, 1.00)	0.093
SpO_2, %	1.07 (0.97, 1.17)	0.198	1.26 (0.98, 1.64)	0.076
NT-pBNP, ng/L	1.00 (1.00, 1.00)	0.398	1.006 (1.00, 1.01)	0.081
Troponin T, ng/L	0.98 (0.94, 1.01)	0.207	0.89 (0.79, 0.99)	0.032
NfL, pg/mL	0.97 (0.91, 1.01)	0.122	0.92 (0.77, 1.03)	0.241
GFAp, pg/mL	1.00 (0.99, 1.00)	0.070	1.00 (0.99, 1.00)	0.797
D-dimer[g], mg/L	0.65 (0.35, 1.19)	0.166	0.86 (0.18, 3.84)	0.851
Ferritin, µg/L	1.00 (1.00, 1.00)	0.200	1.00 (0.99, 1.00)	0.826
Vitamin B_{12}, pmol/L	1.00 (1.00, 1.00)	0.372	1.00 (1.00, 1.00)	0.011
**Immunological markers**				
Blood Leukocyte count, 10^9 cells/L	1.05 (0.98 to 1.12)	0.187	0.98 (0.80 to 1.17)	0.812
Blood Lymphocyte count, 10^9 cells/L	0.99 (0.82 to 1.18)	0.882	0.64 (0.37, 1.06)	0.085
Blood Monocyte count, 10^9 cells/L	1.71 (0.86 to 3.31)	0.124	0.624 (0.09, 3.66)	0.615
Blood Neutrophil count, 10^9 cells/L	1.09 (0.99 to 1.19)	0.067	1.15 (0.90, 1.42)	0.255
Neutrophil-to-Lymphocyte ratio	1.17 (1.00 to 1.36)	0.053	1.49 (1.02, 2.10)	0.043
Systemic immune-inflammation index [h]	1.00 (1.000, 1.001)	0.017	1.002 (1.00, 1.00)	0.010
hsCRP[i], mg/L	0.99 (0.91, 1.08)	0.848	1.02 (0.83, 1.26)	0.842
GDF15, ng/mL	1.28 (0.64, 2.39)	0.477	2.41 (0.41, 10.35)	0.309
TCC/C5b-9, CAU/mL	1.03 (0.95, 1.09)	0.425	0.90 (0.31, 1.34)	0.723
RANTES/CCL5[j], pg/mL	1.02 (0.90, 1.15)	0.740	1.04 (0.76, 1.37)	0.806
MCP-1/CCL2, pg/mL	1.00 (0.98, 1.01)	0.543	1.01 (0.96, 1.05)	0.717
IP-10, pg/mL	1.00 (1.00, 1.00)	0.130	1.00 (1.00, 1.00)	0.939
SARS-CoV-2-Anti-RBD, BAU/mL	1.00 (1.00, 1.00)	0.079	1.00 (1.00, 1.00)	0.053
Plasma total IgG, g/L	1.01 (0.96 to 1.06)	0.744	1.01 (0.89, 1.14)	0.912
Plasma total IgM, g/L	1.01 (0.82 to 1.23)	0.924	0.98 (0.57, 1.60)	0.923

© 2023 Selvakumar J et al. *JAMA Network Open.*

Plasma total IgA, g/L	1.05 (0.91 to 1.20)	0.504	0.85 (0.58, 1.21)	0.373
Plasma IL-1β, pg/mL	0.93 (0.81, 1.06)	0.305	1.09 (0.79, 1.42)	0.567
Plasma IL-2, pg/mL	1.00 (0.93 to 1.07)	0.980	1.13 (0.96, 1.30)	0.145
Plasma IL-4, pg/mL	0.96 (0.83 to 1.10)	0.522	1.06 (0.75, 1.47)	0.725
Plasma IL-7, pg/mL	1.00 (0.99 to 1.01)	0.974	0.98 (0.94, 1.01)	0.102
Plasma IL-8, pg/mL	1.01 (0.96 to 1.05)	0.805	1.08 (0.98, 1.15)	0.109
Plasma IL-9, pg/mL	1.00 (1.00 to 1.00)	0.562	1.00 (1.00, 1.00)	0.712
Plasma IL-12, pg/mL	1.00 (0.98 to 1.02)	0.973	1.03 (0.97, 1.07)	0.346
Plasma IL-13, pg/mL	0.98 (0.88 to 1.06)	0.611	0.96 (0.69, 1.16)	0.726
Plasma IL-17A, pg/mL	1.00 (0.95 to 1.06)	0.962	1.10 (0.96, 1.24)	0.158
Plasma TNF, pg/mL	1.00 (0.98 to 1.01)	0.482	0.98 (0.93, 1.02)	0.402
Plasma IFN-γ, pg/mL	1.01 (0.98 to 1.03)	0.600	1.05 (1.00, 1.09)	0.062
Plasma Eotaxin-1/CCL11, pg/mL	0.99 (0.98 to 1.01)	0.390	1.00 (0.96, 1.04)	0.889
Plasma MIP-1α, pg/mL	1.17 (0.90 to 1.51)	0.249	1.06 (0.53, 2.04)	0.863
Plasma MIP-1β, pg/mL	1.00 (1.00 to 1.00)	0.570	1.00 (0.99, 1.01)	0.794
Plasma GM-CSF, pg/mL	1.02 (0.96 to 1.07)	0.487	1.08 (0.96, 1.17)	0.194
Plasma bFGF, pg/mL	1.01 (0.99 to 1.02)	0.395	1.03 (1.00, 1.06)	0.056
Plasma C3bc, ng/mL	0.98 (0.92 to 1.05)	0.611	0.94 (0.78, 1.11)	0.450
**Autonomic markers**				
LF-RRI[i], ms^2	1.01 (0.90, 1.12)	0.928	0.79 (0.60, 1.04)	0.088
HF-RRI[i], ms^2	1.00 (0.91, 1.10)	0.970	0.94 (0.72, 1.18)	0.591
**Cognitive function tests**				
Digit span[k], total score	1.00 (0.97, 1.03)	0.751	1.05 (0.97, 1.12)	0.207
Immediate recall[l], score 0 to 36	0.98 (0.96, 1.01)	0.157	1.03 (0.97, 1.10)	0.384
Delayed recall[l], score 0 to 12	1.01 (0.96, 1.06)	0.783	1.07 (0.94, 1.23)	0.308
Recognition index[m], score 0 to 12	1.10 (0.98, 1.24)	0.102	1.16 (0.87, 1.61)	0.332
**Clinical symptoms**				
Fatigue[n], score 0 to 33	1.06 (1.05, 1.08)	<0.001	1.22 (1.18, 1.27)	<0.001
Post-exertional malaise[o], score 0 to 100	1.01 (1.01, 1.02)	<0.001	1.04 (1.03, 1.05)	<0.001
Sleep problems[p], score 1 to 6	0.68 (0.62, 0.74)	<0.001	0.33 (0.26, 0.41)	<0.001
Pain[q], score 1 to 10	1.26 (1.17, 1.35)	<0.001	1.64 (1.38, 1.93)	<0.001
Cognitive symptoms[r], score 3 to 15	1.11 (1.08, 1.15)	<0.001	1.31 (1.23, 1.41)	<0.001
Respiratory symptoms[s], score 2 to 10	1.12 (1.07, 1.17)	<0.001	1.30 (1.16, 1.45)	<0.001
Autonomic symptoms[t], score 2 to 10	1.11 (1.07, 1.14)	<0.001	1.34 (1.25, 1.44)	<0.001
Symptoms of anxiety[u], score 0 to 21	1.09 (1.07, 1.12)	<0.001	1.23 (1.16, 1.30)	<0.001
Symptoms of depression[u], score 0 to 21	1.10 (1.08, 1.13)	<0.001	1.23 (1.16, 1.30)	<0.001
Negative emotions[v], score 5 to 25	1.06 (1.04, 1.08)	<0.001	1.14 (1.09, 1.19)	<0.001
Principal Component: Symptom severity[w]	1.54 (1.40, 1.69)	<0.001	3.08 (2.53, 3.75)	<0.001
**Psychological traits**				
Neuroticism[x], score 0 to 24	1.07 (1.05, 1.08)	<0.001	1.13 (1.09, 1.17)	<0.001
Emotional awareness[y], score 7 to 35	1.06 (1.05, 1.08)	<0.001	1.11 (1.07, 1.16)	<0.001
Worrying tendencies[z], score 16 to 80	1.03 (1.02, 1.03)	<0.001	1.06 (1.04, 1.08)	<0.001
Body vigilance[aa], score 0 to 40	1.03 (1.02, 1.04)	<0.001	1.06 (1.02, 1.09)	<0.001
Principal component: Emotional maladjustment[ab]	1.51 (1.37, 1.67)	<0.001	2.27 (1.80, 2.89)	<0.001
**Social/behavioural markers**				
Average level of physical activity prior to acute infection[ac], score 1 to 10	0.91 (0.87, 0.95)	<0.001	0.87 (0.77, 0.98)	0.021
Socioeconomic level ISEI-08[ad], score 10 to 90	1.00 (0.99, 1.00)	0.062	0.99 (0.98, 1.01)	0.375

© 2023 Selvakumar J et al. *JAMA Network Open.*

Family member with chronic disease[ae]	1.36 (1.11, 1.68)	0.004	1.82 (1.06, 3.11)	0.030
Loneliness[af], score 20-80	1.03 (1.03, 1.04)	<0.001	1.07 (1.05, 1.09)	<0.001
Negative life events last 12 months[ag], impact score	1.05 (1.03, 1.07)	<0.001	1.10 (1.04, 1.15)	<0.001
Negative life events prior to last 12 months[ag], impact score	1.05 (1.00, 1.09)	0.039	1.06 (0.94, 1.17)	0.315

CI=95% Confidence interval; NA=Not applicable; SARS-CoV-2= Severe acute respiratory syndrome coronavirus 2; BMI=Body mass index; FVC=Forced vital capacity; SpO_2=Peripheral oxygen saturation; NT-pBNP=N-terminal pro-brain natriuretic peptide; NfL=Neurofilament light chain; GFAp=Glial fibrillary acidic protein; hsCRP=high-sensitive assay of C-reactive protein; GDF-15=Growth/differentiation factor 15; IL=Interleukin; TCC=Terminal complement complex; CAU=Complement arbitrary units; RANTES=Regulated on activation, normal T-cell expressed and secreted; MCP=Monocyte chemotactic protein; IP=Interferon gamma-induced protein; RBD= Receptor binding domain; BAU=Binding antibody units; LF-RRI=Low frequency power of heart rate variability; HF-RRI=High-frequency power of heart rate variability; ISEI-08=International Socioeconomic Index 2008. [a]95% Profile likelihood based confidence intervals. [b]Likelihood ratio p-values. [c]Bonferroni-adjusted for test multiplicity. [d]Standardised score calculated according to World Health Organisation 2006 Child Growth Standards for ages 12-19; for participants above this age, reference values for 19-year-olds were used. [e]One or more doses of immunisation against SARS-CoV-2. [f]The Global Lung Function Initiative 2012 reference values were used to calculate predicted values.[4] [g]Square-root-transformed variable was used for regression analysis. [h]Defined as (NxP)/L, where N, P and L represent neutrophil, platelet and lymphocyte counts respectively. [i]Ln-transformed variable was used for regression analyses. [j]Fifth-root-transformed variable was used for regression analyses. [k]From the Wechsler Intelligence Scales for Children revised; higher score implies better short-term memory. [l]From the Hopkins Verbal Learning Test revised (HVLT-R); higher scores imply better immediate and delayed recall of words, respectively. [m]From the HVLT-R; higher score implies better recognition of words. [n]From the Chalder Fatigue Questionnaire; higher score implies more fatigue. [o]From the DePaul Symptom Questionnaire; higher score implies more post-exertional malaise. [p]From the Karolinska Sleep Questionnaire; higher score implies better sleep. [q]From the Brief Pain Inventory, higher score implies more pain. [r]Self-developed, aggregated score for problems with ‘memory’, ‘concentration’, and ‘decision making’; higher score implies more symptoms. [s]Self-developed, aggregated score for symptoms ‘cough’ and ‘dyspnoea’; higher score implies more symptoms. [t]Self-developed, aggregated score for symptoms ‘dizziness’, ‘cold and pale hands’, ‘feeling alternately warm and cold’; higher score implies more symptoms. [u]From the anxiety and depression subscales, respectively, of the Hospital Anxiety and Depression Scale; higher scores imply more symptoms. [v]From the Positive and Negative Affect Schedule; higher score implies more negative emotions. [w]The main component extracted by Principal Component Analysis of the 10 clinical symptoms variables, labelled ‘symptom severity’. [x]From the NEO-Five-Factor-Inventory-30; higher scores implies more neuroticism. [y]From the Toronto Alexithymia Scale; higher score implies more difficulty identifying feelings. [z]From the Penn State Worry Questionnaire; higher score implies more worrying. [aa]From the Body Vigilance Scale; higher score implies being more attentive to bodily sensations. [ab]The main component extracted by Principal Component Analysis of the four psychological traits variables, labelled ‘emotional maladjustment’. [ac]Self-developed; higher score implies more physical activity. [ad]The ISEI-08 score of the parent with the highest score; higher score implies higher socioeconomic status. [ae]Having a sibling or parent affected by chronic disease. [af]From the University of California, Los Angeles, Loneliness Scale; higher score implies more loneliness. [ag]From the Life Event Checklist; higher score implies more negative impact of past life events.

© 2023 Selvakumar J et al. *JAMA Network Open.*

Table S18. Characteristics of potential baseline risk factors and their univariate associations (Poisson regression with log-link and robust error variances) to long COVID and post-infective fatigue syndrome (PIFS) at six months follow-up. Sensitivity analysis featuring imputation of mean/median values for missing data.

	**Baseline characteristics**		**Univariate association to long COVID**		**Univariate association to PIFS**	
	SARS-CoV-2-positive (n=382)	*SARS-CoV-2-negative (n=85)*	*Relative risk (CI)[a]*	*p-value[b]*	*Relative risk (CI)[a]*	*p-value[b]*
**SARS-CoV-2 status**						
SARS-CoV-2-positive at baseline – no. (%)	NA	NA	1.03 (.81, 1.33)	0.804	1.70 (.86, 3.85)	0.133
**Background and constitutional factors**						
Female sex – no. (%)	230 (60.2)	54 (63.5)	1.48 (1.21, 1.82)	<0.001	3.66 (1.97, 7.53)	<0.001
Age, years – mean (CI)	18.0 (17.6, 18.4)	17.7 (17.0, 18.4)	1.01 (.98, 1.03)	0.678	1.08 (1.01, 1.15)	0.031
BMI, z-score[d] – mean (CI)	0.44 (0.32, 0.55)	0.48 (0.23, 0.71)	1.01 (.93, 1.09)	0.858	0.94 (0.76, 1.15)	0.522
Ethnicity non-European – no. (%)	88 (23)	2 (2.4)	1.09 (.86, 1.37)	0.479	1.67 (0.96, 2.77)	0.068
Any comorbidity – no. (%)	79 (20.7)	28 (32.9)	1.36 (1.10, 1.68)	0.004	1.43 (0.84, 2.36)	0.186
**Observational period characteristics**						
Time span between baseline and follow-up, days – median (range)	193 (191, 195)	193 (190, 196)	1.00 (1.00, 1.01)	0.369	1.00 (0.98, 1.01)	0.710
Immunisation against SARS-CoV-2[e] – no. (%)	4 (1.0)	3 (3.5)	0.89 (0.34, 1.83)	0.769	2.25 (0.43, 6.76)	0.285
**Organ function tests/biomarkers**						
FVC, % of predicted[f] – mean (CI)	99.4 (98.5, 100.4)	100.8 (98.6, 103.0)	1.00 (0.99, 1.01)	0.756	0.99 (0.96, 1.01)	0.245
SpO_2, % – mean (CI)	98.7 (98.6, 98.8)	98.6 (98.3, 98.8)	1.06 (0.97, 1.15)	0.199	1.24 (1.00, 1.57)	0.056
NT-pBNP, ng/L – median (CI)	34.5 (34.0, 36.0),	34.0 (27.0, 43.0)	1.00 (1.00, 1.00)	0.384	1.01 (1.00, 1.01)	0.115
Troponin T, ng/L – median (CI)	4.0 (4.0, 4.0),	2.9 (2.2, 4.0)	0.98 (0.95, 1.02)	0.332	0.88 (0.79, 0.97)	0.011
NfL, pg/mL – mean (CI)	4.72 (4.34, 5.12)	4.20 (3.86, 4.55)	0.97 (0.92, 1.01)	0.117	0.96 (0.83, 1.04)	0.460
GFAp, pg/mL – mean (CI)	70.0 (62.3, 53.4)	56.0 (51.1, 61.0)	1.00 (0.99, 1.00)	0.038	1.00 (0.99, 1.00)	0.534
D-dimer[g], mg/L – median (CI)	0.178 (0.154, 0.192)	0.189 (0.168, 0.207)	0.68 (0.39, 1.19)	0.181	1.18 (0.29, 4.44)	0.814
Ferritin, µg/L – median (CI)	69 (67, 72)	48 (42, 56)	1.00 (1.00, 1.00)	0.150	1.00 (.99, 1.00)	0.620
Vitamin B_{12}, pmol/L – mean (CI)	444 (461, 433)	419 (388, 451)	1.00 (1.00, 1.00)	0.160	0.998 (0.996, 0.999)	0.009
**Immunological markers**						
Blood Leukocyte count, 10^9 cells/L - mean (CI)	6.9 (6.6, 7.3)	6.8 (6.0, 7.6)	1.00 (0.98, 1.03)	0.773	0.99 (0.92, 1.05)	0.803
Blood Lymphocyte count, 10^9 cells/L - mean (CI)	2.13 (2.07, 2.18)	2.06 (1.95, 2.17)	0.97 (0.82, 1.15)	0.75	0.73 (0.45, 1.13)	0.161
Blood Monocyte count, 10^9 cells/L - mean (CI)	0.45 (0.44, 0.47)	0.42 (0.39, 0.45)	1.46 (0.77, 2.69)	0.242	0.52 (0.09, 2.63)	0.44
Blood Neutrophil count, 10^9 cells/L - mean (CI)	3.16 (3.05, 3.28)	3.07 (2.85, 3.29)	1.07 (0.98, 1.15)	0.139	1.07 (0.86, 1.30)	0.531
Neutrophil-to-Lymphocyte ratio – mean (CI)	1.56 (1.50, 1.63)	1.58 (1.44, 1.71)	1.12 (0.97, 1.28)	0.134	1.27 (0.89, 1.75)	0.18
Systemic immune-inflammation index - median (CI) [h]	410.8 (390.8, 430.8)	395.8 (361.5, 430.1)	1.00 (1.00, 1.00)	0.056	1.001 (1.001, 1.002)	0.035
hsCRP[i], mg/L – median (CI)	0.830 (0.732, 0.939)	1.26 (0.697, 1.99)	0.99 (0.92, 1.06)	0.709	1.04 (0.87, 1.26)	0.655
GDF15, ng/mL – mean (CI)	0.407 (0.390, 0.424)	0.399 (0.364, 0.434)	0.98 (0.54, 1.66)	0.933	2.27 (0.68, 5.64)	0.165
TCC/C5b-9, CAU/mL – median (CI)	0.180 (0.170, 0.190)	0.003 (0.002, 0.050)	1.01 (0.93, 1.07)	0.736	1.08 (0.95, 1.16)	0.178
RANTES/CCL5[j], pg/mL – median (CI)	265.7 (248.5, 283.5)	265.6 (221.3, 320.2)	1.02 (0.91, 1.13)	0.767	1.03 (0.77, 1.32)	0.850
MCP-1/CCL2, pg/mL – mean (CI)	12.8 (12.2, 13.4)	13.8 (12.6, 15.0)	1.00 (0.98, 1.01)	0.724	1.01 (0.97, 1.05)	0.602
IP-10, pg/mL – mean (CI)	164.1 (155.6, 172.6)	126.0 (114.1, 137.9)	1.00 (1.00, 1.00)	0.037	1.00 (1.00, 1.00)	0.868
SARS-CoV-2-Anti-RBD, BAU/mL – median (CI)	1044 (988.7, 1129.9)	1 (1, 1)	1.00 (1.00, 1.00)	0.363	1.00 (1.00, 1.00)	0.920
Plasma total IgG, g/L - mean (CI)	11.1 (10.9, 11.3)	10.7 (10.3, 11.1)	1.02 (0.97, 1.06)	0.497	1.03 (0.92, 1.15)	0.612
Plasma total IgM, g/L - mean (CI)	1.27 (1.22, 1.32)	1.10 (0.99, 1.21)	1.00 (0.83, 1.20)	0.995	0.89 (0.55, 1.40)	0.623
Plasma total IgA, g/L - mean (CI)	1.71 (1.63, 1.78)	1.58 (1.43, 1.74)	1.06 (0.93, 1.20)	0.370	0.88 (0.63, 1.21)	0.458

© 2023 Selvakumar J et al. *JAMA Network Open.*

Plasma IL-1β, pg/mL – median (CI)	0.630 (0.490, 0.730)	0.009 (0.007, 0.190)	0.93 (0.82, 1.05)	0.254	1.06 (0.80, 1.34)	0.653
Plasma IL-2, pg/mL - median (CI)	0.69 (0.63, 0.78)	0.40 (0.03, 0.78)	0.99 (0.92, 1.05)	0.669	1.07 (0.92, 1.23)	0.369
Plasma IL-4, pg/mL - median (CI)	1.46 (1.40, 1.50)	0.88 (0.75, 0.92)	0.98 (0.87, 1.11)	0.775	1.04 (0.76, 1.39)	0.792
Plasma IL-7, pg/mL - median (CI)	12.6 (12.1, 12.6)	2.98 (1.79, 5.65)	1.00 (0.99, 1.01)	0.648	0.98 (0.96, 1.01)	0.182
Plasma IL-8, pg/mL - median (CI)	0.80 (0.69, 1.08)	0.10 (0.08, 0.12)	0.99 (0.95, 1.03)	0.756	1.06 (0.99, 1.12)	0.099
Plasma IL-9, pg/mL - median (CI)	68.2 (64.1, 77.0)	70.2 (51.5, 86.4)	1.00 (1.00, 1.00)	0.634	1.00 (1.00, 1.00)	0.833
Plasma IL-12, pg/mL - median (CI)	1.49 (1.38, 1.50)	0.19 (0.14, 1.05)	1.00 (0.97, 1.02)	0.735	1.01 (0.96, 1.06)	0.613
Plasma IL-13, pg/mL - median (CI)	0.26 (0.26, 0.27)	0.51 (0.45, 0.66)	0.97 (0.89, 1.05)	0.512	0.95 (0.72, 1.14)	0.651
Plasma IL-17A, pg/mL - median (CI)	1.62 (1.62, 1.99)	1.35 (0.69, 2.03)	1.01 (0.95, 1.06)	0.856	1.07 (0.95, 1.19)	0.266
Plasma TNF-α, pg/mL - median (CI)	7.81 (6.91, 8.19)	4.26 (3.04, 5.40)	0.99 (0.98, 1.01)	0.376	0.99 (0.94, 1.02)	0.432
Plasma IFN-γ, pg/mL - median (CI)	1.30 (1.14, 1.32)	0.94 (0.94, 1.14)	1.00 (0.97, 1.02)	0.971	1.03 (0.98, 1.06)	0.235
Plasma Eotaxin-1/CCL11, pg/mL - median (CI)	14.8 (14.1, 14.9)	12.7 (11.6, 14.0)	1.00 (0.98, 1.01)	0.568	1.00 (0.97, 1.04)	0.854
Plasma MIP-1α, pg/mL - median (CI)	0.77 (0.75, 0.77)	0.79 (0.79, 1.02)	1.09 (0.86, 1.39)	0.467	0.94 (0.50, 1.72)	0.849
Plasma MIP-1β, pg/mL - median (CI)	24.9 (23.1, 26.2)	25.2 (19.4, 30.0)	1.00 (1.00, 1.00)	0.613	1.00 (0.99, 1.01)	0.929
Plasma GM-CSF, pg/mL - median (CI)	0.20 (0.11, 0.34)	0.017 (0.014, 0.023)	1.01 (0.96, 1.06)	0.687	1.05 (0.92, 1.15)	0.423
Plasma bFGF, pg/mL - median (CI)	3.40 (2.72, 3.40)	1.32 (1.08, 1.54)	1.00 (0.99, 1.02)	0.538	1.02 (0.99, 1.05)	0.138
Plasma C3bc, ng/mL - median (CI)	3.83 (3.72, 3.99)	2.92 (2.70, 3.15)	0.99 (0.93, 1.05)	0.686	0.96 (0.83, 1.11)	0.615
**Autonomic markers**						
LF-RRI[i], ms^2 – median (CI)	649 (595, 745)	585 (467, 841)	0.99 (0.90, 1.10)	0.866	0.78 (0.61, 0.99)	0.045
HF-RRI[i], ms^2 – median (CI)	758 (693, 880)	1004 (724, 1253)	0.99 (0.91, 1.08)	0.804	0.92 (0.74, 1.13)	0.419
**Cognitive function tests**						
Digit span[k], total score – median (CI)	15.1 (14.8, 15.5)	15.0 (14.3, 15.7)	1.00 (0.97, 1.02)	0.734	1.02 (0.96, 1.09)	0.489
Immediate recall[l], score 0 to 36 – median (CI)	24.6 (24.2, 25.0)	24.6 (23.8, 25.4)	0.98 (0.96, 1.01)	0.168	1.02 (0.97, 1.08)	0.461
Delayed recall[l], score 0 to 12 – median (CI)	8.73 (8.52, 8.93)	8.45 (8.07, 8.84)	1.01 (0.96, 1.05)	0.840	1.07 (0.95, 1.21)	0.278
Recognition index[m], score 0 to 12 – median (CI)	12 (11, 12)	12 (11, 12)	1.08 (0.97, 1.20)	0.147	1.23 (0.94, 1.67)	0.136
**Clinical symptoms**						
Fatigue[n], score 0 to 33 – mean (CI)	16.2 (15.6, 16.7)	13.3 (12.2, 14.3)	1.06 (1.05, 1.08)	<0.001	1.22 (1.18, 1.26)	<0.001
Post-exertional malaise[o], score 0 to 100 – median (CI)	20 (20, 20)	10 (10, 15)	1.01 (1.01, 1.02)	<0.001	1.04 (1.03, 1.04)	<0.001
Sleep problems[p], score 1 to 6 – mean (CI)	4.05 (4.16, 4.07)	3.83, (3.65, 4.02)	0.69 (0.63, 0.75)	<0.001	0.36 (0.30, 0.44)	<0.001
Pain[q], score 1 to 10 – median (CI)	2.25 (2.0, 2.25)	2.5 (2.20, 2.75)	1.25 (1.16, 1.33)	<0.001	1.67 (1.44, 1.93)	<0.001
Cognitive symptoms[r], score 3 to 15 – median (CI)	6 (5, 6)	6 (5, 8)	1.12 (1.09, 1.15)	<0.001	1.30 (1.22, 1.39)	<0.001
Respiratory symptoms[s], score 2 to 10 – median (CI)	4 (4, 4)	3 (3, 3)	1.12 (1.07, 1.17)	<0.001	1.29 (1.17, 1.42)	<0.001
Autonomic symptoms[t], score 2 to 10 - median (CI)	6 (5, 6)	5 (5, 6)	1.11 (1.08, 1.14)	<0.001	1.33 (1.25, 1.42)	<0.001
Symptoms of anxiety[u], score 0 to 21 – median (CI)	5 (5, 6)	7 (6, 8)	1.09 (1.06, 1.11)	<0.001	1.19 (1.14, 1.25)	<0.001
Symptoms of depression[u], score 0 to 21 – median (CI)	3 (3, 3)	3 (3, 5)	1.10 (1.08, 1.12)	<0.001	1.21 (1.15, 1.27)	<0.001
Negative emotions[v], score 5 to 25 – median (CI)	9 (9, 10)	11 (10, 13)	1.06 (1.04, 1.08)	<0.001	1.13 (1.09, 1.18)	<0.001
Principal Component: Symptom severity[w] – mean (CI)			1.54 (1.41, 1.67)	<0.001	3.03 (2.53, 3.64)	<0.001
**Psychological traits**						
Neuroticism[x], score 0 to 24 – median (CI)	6 (5, 6)	7 (5, 11)	1.06 (1.05, 1.08)	<.0001	1.12 (1.08, 1.16)	<.0001
Emotional awareness[y], score 7 to 35 – median (CI)	13 (13, 14)	14 (12, 17)	1.06 (1.04, 1.07)	<0.001	1.11 (1.07, 1.15)	<0.001
Worrying tendencies[z], score 16 to 80 – mean (CI)	45.0 (43.6, 46.4)	47.7 (44.8, 50.7)	1.02 (1.02, 1.03)	<0.001	1.06 (1.04, 1.07)	<0.001
Body vigilance[aa], score 0 to 40 – mean (CI)	12.0 (11.3, 12.8)	11.9 (10.4, 13.4)	1.03 (1.02, 1.04)	<0.001	1.06 (1.03, 1.09)	<0.001
Principal component: Emotional maladjustment[ab] – mean (CI)			1.48 (1.35, 1.62)	<0.001	2.22 (1.80, 2.75)	<0.001
**Social/behavioural markers**						
Average level of physical activity prior to acute infection[ac], score 1 to 10 – mean (CI)	6.42 (6.20, 6.64)	6.17 (5.72, 6.61)	0.92 (0.88, 0.96)	<0.001	0.86 (0.77, 0.96)	0.007
Socioeconomic level ISEI-08[ad], score 10 to 90 – median (CI)	63.3 (63.3, 64.4)	65.0 (59.9, 68.7)	1.00 (0.99, 1.00)	0.054	1.00 (0.98, 1.01)	0.477

© 2023 Selvakumar J et al. *JAMA Network Open.*

Family member with chronic disease[ae] – no. (%)	123 (32.2)	30 (35.3)	1.37 (1.13, 1.65)	0.002	1.90 (1.18, 3.06)	0.009
Loneliness[af], score 20-80 – mean (CI)	37.7 (36.6, 38.7)	39.4 (37.0, 41.8)	1.03 (1.02, 1.04)	<0.001	1.07 (1.04, 1.09)	<0.001
Negative life events last 12 months[ag], impact score – median (CI)	2 (2, 2)	2 (2, 3)	1.05 (1.03, 1.07)	<0.001	1.09 (1.04, 1.14)	<0.001
Negative life events prior to last 12 months[ag], impact score – median (CI)	0 (0, 0)	2 (0, 3)	1.05 (1.01, 1.09)	0.012	1.01 (.90, 1.11)	0.898

CI=95% Confidence interval; NA=Not applicable; SARS-CoV-2= Severe acute respiratory syndrome coronavirus 2; BMI=Body mass index; FVC=Forced vital capacity; SpO_2=Peripheral oxygen saturation; NT-pBNP=N-terminal pro-brain natriuretic peptide; NfL=Neurofilament light chain; GFAp=Glial fibrillary acidic protein; hsCRP=high-sensitive assay of C-reactive protein; GDF-15=Growth/differentiation factor 15; IL=Interleukin; TCC=Terminal complement complex; CAU=Complement arbitrary units; RANTES=Regulated on activation, normal T-cell expressed and secreted; MCP=Monocyte chemotactic protein; IP=Interferon gamma-induced protein; RBD= Receptor binding domain; BAU=Binding antibody units; LF-RRI=Low frequency power of heart rate variability; HF-RRI=High-frequency power of heart rate variability; ISEI-08=International Socioeconomic Index 2008. [a]95% Profile likelihood based confidence intervals. [b]Likelihood ratio p-values. [c]Bonferroni-adjusted for test multiplicity. [d]Standardised score calculated according to World Health Organisation 2006 Child Growth Standards for ages 12-19; for participants above this age, reference values for 19-year-olds were used. [e]One or more doses of immunisation against SARS-CoV-2. [f]The Global Lung Function Initiative 2012 reference values were used to calculate predicted values.[4] [g]Square-root-transformed variable was used for regression analysis. [h]Defined as (NxP)/L, where N, P and L represent neutrophil, platelet and lymphocyte counts respectively. [i]Ln-transformed variable was used for regression analyses. [j]Fifth-root-transformed variable was used for regression analyses. [k]From the Wechsler Intelligence Scales for Children revised; higher score implies better short-term memory. [l]From the Hopkins Verbal Learning Test revised (HVLT-R); higher scores imply better immediate and delayed recall of words, respectively. [m]From the HVLT-R; higher score implies better recognition of words. [n]From the Chalder Fatigue Questionnaire; higher score implies more fatigue. [o]From the DePaul Symptom Questionnaire; higher score implies more post-exertional malaise. [p]From the Karolinska Sleep Questionnaire; higher score implies better sleep. [q]From the Brief Pain Inventory, higher score implies more pain. [r]Self-developed, aggregated score for problems with 'memory', 'concentration', and 'decision making'; higher score implies more symptoms. [s]Self-developed, aggregated score for symptoms 'cough' and 'dyspnoea'; higher score implies more symptoms. [t]Self-developed, aggregated score for symptoms 'dizziness', 'cold and pale hands', 'feeling alternately warm and cold'; higher score implies more symptoms. [u]From the anxiety and depression subscales, respectively, of the Hospital Anxiety and Depression Scale; higher scores imply more symptoms. [v]From the Positive and Negative Affect Schedule; higher score implies more negative emotions. [w]The main component extracted by Principal Component Analysis of the 10 clinical symptoms variables, labelled 'symptom severity'. [x]From the NEO-Five-Factor-Inventory-30; higher scores implies more neuroticism. [y]From the Toronto Alexithymia Scale; higher score implies more difficulty identifying feelings. [z]From the Penn State Worry Questionnaire; higher score implies more worrying. [aa]From the Body Vigilance Scale; higher score implies being more attentive to bodily sensations. [ab]The main component extracted by Principal Component Analysis of the four psychological traits variables, labelled 'emotional maladjustment'. [ac]Self-developed; higher score implies more physical activity. [ad]The ISEI-08 score of the parent with the highest score; higher score implies higher socioeconomic status. [ae]Having a sibling or parent affected by chronic disease. [af]From the University of California, Los Angeles, Loneliness Scale; higher score implies more loneliness. [ag]From the Life Event Checklist; higher score implies more negative impact of past life events.

© 2023 Selvakumar J et al. *JAMA Network Open.*

Table S19. Baseline potential risk factors of long COVID and post-infective fatigue syndrome (PIFS) at six months follow-up. Final multiple regression models (modified Poisson regression with log-link and robust error variances). Sensitivity analysis removing cases of uncertain classification, individuals with possible EBV-infection at inclusion or during the observational period, individuals vaccinated before baseline and individuals receiving vaccination less than five days prior to the six months assessment.

	Long COVID		PIFS	
	Relative risk (CI)[a]	*p-value*[b]	*Relative risk (CI)*[a]	*p-value*[b]
**SARS-CoV-2 status**				
SARS-CoV-2-positive at baseline	1.00 (0.76, 1.32)	0.987	1.24 (0.62, 2.71)	0.565
**Background and constitutional factors**				
Female sex	1.22 (0.98, 1.54)	0.080	1.59 (0.88, 3.07)	0.131
Age, years	0.98 (0.95, 1.01)	0.216	1.05 (0.99, 1.12)	0.133
BMI, z-score[c]	0.99 (0.90, 1.08)	0.772	0.97 (0.77, 1.22)	0.765
Ethnicity non-European	0.88 (0.67, 1.15)	0.358	1.11 (0.57, 2.04)	0.753
Any comorbidity	1.13 (0.89, 1.42)	0.282	0.68 (0.39, 1.14)	0.146
**Observational period characteristics**				
Time span between baseline and follow-up, days	1.00 (1.00, 1.01)	0.864	0.99 (0.98, 1.01)	0.362
Immunisation against SARS-CoV-2[d]	NA	NA	NA	NA
**Remaining risk factors**				
Principal component: Symptom severity[e]	1.41 (1.27, 1.57)	<0.001	3.28 (2.57, 4.23)	<0.001
Average level of physical activity prior to acute infection[f], score 1 to 10	0.95 (0.90, 0.99)	0.021	--	--
Loneliness[g], score 20 to 80	1.01 (1.00, 1.02)	0.010	1.03 (1.01, 1.05)	0.008
Blood Leukocyte count, 10^9 cells/L	--	--	0.78 (0.66, 0.92)	0.003
Plasma Interleukin-7, pg/mL	--	--	0.97 (0.95, 1.00)	0.029
LF-RRI[h], ms^2	--	--	0.73 (0.57, 0.92)	0.009

CI=95% Confidence interval; NA=Not applicable; SARS-CoV-2= Severe acute respiratory syndrome coronavirus 2; BMI=Body mass index; NfL=Neurofilament light chain; RBD= Receptor binding domain; BAU=Binding antibody units; LF-RRI=Low frequency power of heart rate variability. [a]95% Profile likelihood based confidence intervals. [b]Likelihood ratio p-values. [c]Standardised score calculated according to World Health Organisation 2006 Child Growth Standards for ages 12-19; for participants above this age, reference values for 19-year-olds were used. [d]One or more doses of immunisation against SARS-CoV-2. [e]The main component extracted by Principal Component Analysis of the 10 clinical symptoms variables, labelled 'symptom severity'. [f]Self-developed; higher score implies more physical activity. [g]From the University of California, Los Angeles, Loneliness Scale; higher score implies more loneliness. [h]Log-transformed variable was used for regression analysis.

© 2023 Selvakumar J et al. *JAMA Network Open.*

Table S20. Baseline potential risk factors of long COVID and post-infective fatigue syndrome (PIFS) at six months follow-up. Final multiple regression models (modified Poisson regression with log-link and robust error variances). Sensitivity analysis removing cases of uncertain classification, individuals with possible EBV-infection at inclusion or during the observational period, individuals vaccinated before baseline, individuals receiving vaccination less than five days prior to the six months assessment, and individuals in the SARS-CoV-2-negative group with general infectious symptoms score[a] ≥ 11 at baseline.

	**Long COVID**		**PIFS**	
	Relative risk (CI)[b]	*p-value*[c]	*Relative risk (CI)*[b]	*p-value*[c]
**SARS-CoV-2 status**				
SARS-CoV-2-positive at baseline	1.03 (0.76, 1.43)	0.857	1.10 (0.47, 3.08)	0.839
**Background and constitutional factors**				
Female sex	1.33 (1.03, 1.74)	0.033	1.43 (0.73, 2.99)	0.308
Age, years	0.98 (0.94, 1.01)	0.121	1.06 (0.99, 1.13)	0.111
BMI, z-score[d]	0.99 (0.89, 1.10)	0.807	0.92 (0.72, 1.18)	0.503
Ethnicity non-European	0.82 (0.61, 1.10)	0.191	1.17 (0.60, 2.18)	0.641
Any comorbidity	1.15 (0.90, 1.47)	0.266	0.62 (0.34, 1.09)	0.099
**Observational period characteristics**				
Time span between baseline and follow-up, days	1.00 (0.99, 1.00)	0.753	0.99 (0.98, 1.01)	0.252
Immunisation against SARS-CoV-2[e]	NA	NA	NA	NA
**Remaining risk factors**				
Principal component: Symptom severity[f]	1.34 (1,16, 1.55)	<0.001	3.35 (2.58, 4.39)	<0.001
NfL, pg/mL	0.95 (0.89, 1.00)	0.050	--	--
Recognition index[g], score 0 to 12	1.13 (1.00, 1.29)	0.044	--	--
Principal component: Emotional maladjustment[h]	1.20 (1.03, 1.39)	0.023	--	--
Socioeconomic level ISEI-08[i], score 10 to 90	0.99 (0.99, 1.00)	0.034	--	--
Loneliness[j], score 20 to 80	--	--	1.03 (1.01, 1.05)	0.013
Blood Leukocyte count, 10^9 cells/L	--	--	0.77 (0.64, 0.91)	0.002
Plasma Interleukin-7, pg/mL			0.97 (0.95, 1.00)	0.038
LF-RRI[k], ms^2	--	--	0.71 (0.55, 0.91)	0.006

CI=95% Confidence interval; NA=Not applicable; SARS-CoV-2= Severe acute respiratory syndrome coronavirus 2; BMI=Body mass index; NfL=Neurofilament light chain; RBD= Receptor binding domain; BAU=Binding antibody units; LF-RRI=Low frequency power of heart rate variability. [a]General infectious symptoms score is computed as the sum across five single items (fever/chills, sore throat, headaches, muscle ache and fatigue after exercise), and has a total range from 5 – 25.[63] [b]95% Profile likelihood based confidence intervals. [c]Likelihood ratio p-values. [d]Standardised score calculated according to World Health Organisation 2006 Child Growth Standards for ages 12-19; for participants above this age, reference values for 19-year-olds were used. [e]One or more doses of immunisation against SARS-CoV-2. [f]The main component extracted by Principal Component Analysis of the 10 clinical symptoms variables, labelled 'symptom severity'. [g]From the Hopkins Verbal Learning Test revised (HVLT-R); higher score implies better recognition of words. [h]The main component extracted by Principal Component Analysis of the four psychological traits variables, labelled 'emotional maladjustment'. [i]The ISEI-08 score of the parent with the highest score; higher score implies higher socioeconomic status. [j]From the University of California, Los Angeles, Loneliness Scale; higher score implies more loneliness. [k]Log-transformed variable was used for regression analysis.

© 2023 Selvakumar J et al. *JAMA Network Open.*

Table S21. Baseline potential risk factors of long COVID and post-infective fatigue syndrome (PIFS) at six months follow-up. Final multiple regression models. Sensitivity analysis featuring imputation of mean/median values for missing data.

	**Long COVID**		**PIFS**	
	Relative risk (CI)[a]	*p-value[b]*	*Relative risk (CI)[a]*	*p-value[b]*
**SARS-CoV-2 status**				
SARS-CoV-2-positive at baseline	1.05 (.82, 1.36)	0.702	1.56 (0.86, 3.00)	0.149
**Background and constitutional factors**				
Female sex	1.15 (0.93, 1.42)	0.206	1.43 (0.85, 2.53)	0.182
Age, years	0.97 (0.94, 1.00)	0.045	1.01 (0.95, 1.07)	0.721
BMI, z-score[c]	0.99 (0.91, 1.07)	0.789	0.86 (0.72, 1.01)	0.069
Ethnicity non-European	0.96 (0.75, 1.21)	0.726	1.03 (0.64, 1.61)	0.904
Any comorbidity	1.13 (0.91, 1.39)	0.267	1.02 (0.65, 1.55)	0.945
**Observational period characteristics**				
Time span between baseline and follow-up, days	1.00 (1.00, 1.01)	0.743	0.99 (0.98, 1.00)	0.067
Immunisation against SARS-CoV-2[d]	0.77 (0.30, 1.62)	0.521	3.12 (0.88, 8.50)	0.074
**Remaining risk factors**				
Principal component: Symptom severity[e]	1.41 (1.27, 1.56)	<0.001	3.47 (2.84, 4.28)	<0.001
Average level of physical activity prior to acute infection[f], score 1 to 10	0.95 (0.91, 1.00)	0.031	--	--
Loneliness[g], score 20 to 80	1.01 (1.00, 1.02)	0.011	--	--
GFAp, pg/mL	1.00 (0.99, 1.00)	0.029	--	--
D-dimer, mg/L	0.56 (0.32, 0.97)	0.040	--	--
Immediate recall[h], score 0 to 36	--	--	1.05 (1.01, 1.10)	0.030
Negative life events prior to last 12 months[i], impact score	--	--	0.90 (0.82, 0.98)	0.011
LF-RRI[j]. ms^2	--	--	0.67 (0.55, 0.82)	<0.001
Blood Lymphocyte count, 10^9 cells/L	--	--	0.67 (0.47, 0.92)	0.013
Plasma Interleukin-7, pg/mL	--	--	0.97 (0.95, 0.99)	0.003

CI=95% Confidence interval; SARS-CoV-2= Severe acute respiratory syndrome coronavirus 2; BMI=Body mass index; GFAp=Glial fibrillary acidic protein; LF-RRI=Low frequency power of heart rate variability. [a]95% Profile likelihood based confidence intervals. [b]Likelihood ratio p-values. [c]Standardised score calculated according to World Health Organisation 2006 Child Growth Standards for ages 12-19; for participants above this age, reference values for 19-year-olds were used. [d]One or more doses of immunisation against SARS-CoV-2. [e]The main component extracted by Principal Component Analysis of the 10 clinical symptoms variables, labelled 'symptom severity'. [f]Self-developed; higher score implies more physical activity. [g]From the University of California, Los Angeles, Loneliness Scale; higher score implies more loneliness. [h]From the Hopkins Verbal Learning Test revised (HVLT-R); higher scores imply better immediate recall of words. [i]From the Life Event Checklist; higher score implies more negative impact of past life events. [j]Log-transformed variable was used for regression analysis.

© 2023 Selvakumar J et al. *JAMA Network Open.*

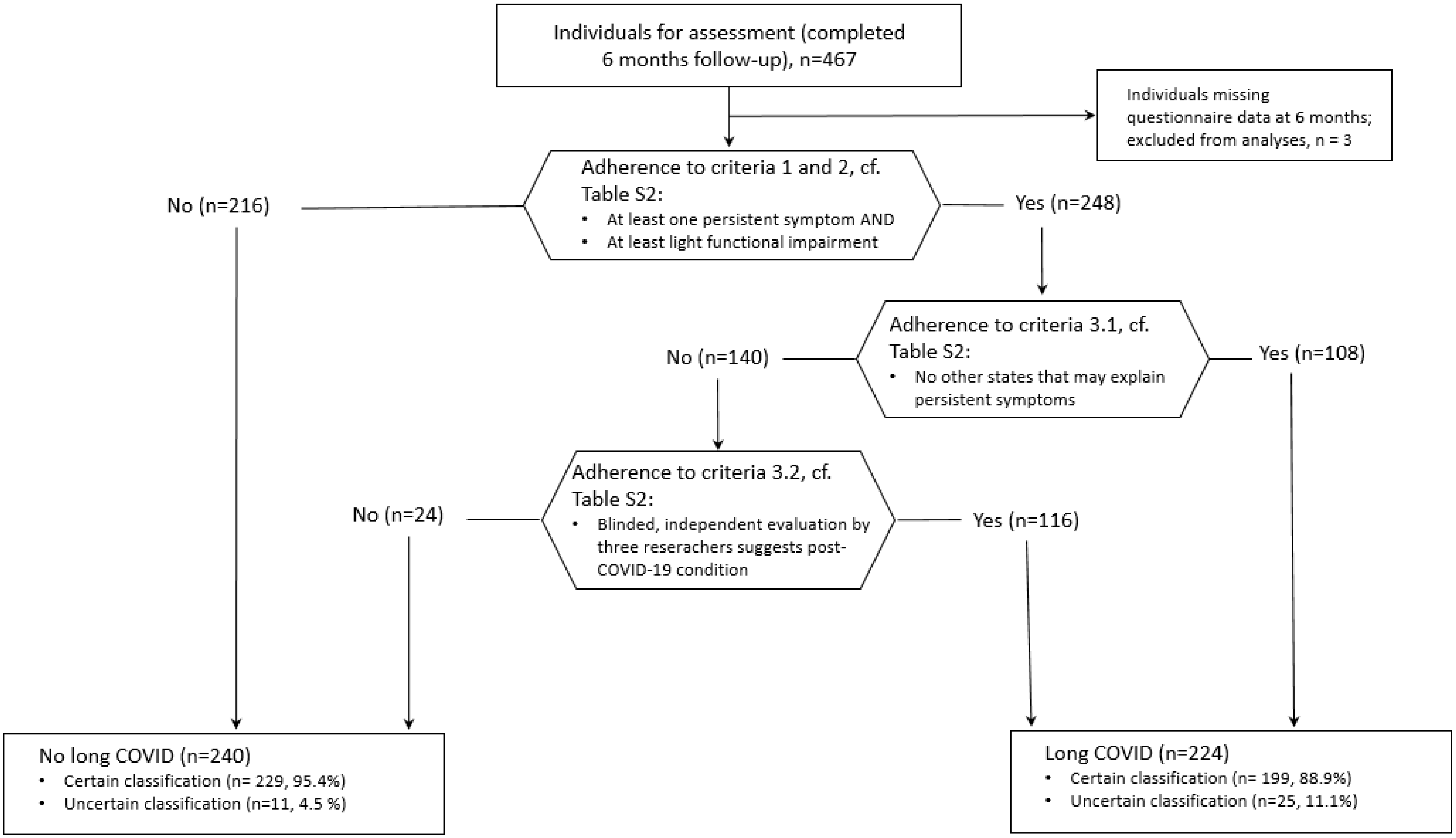

Figure S1. Algorithm for assessment of long COVID at six months follow-up.

© 2023 Selvakumar J et al. *JAMA Network Open.*

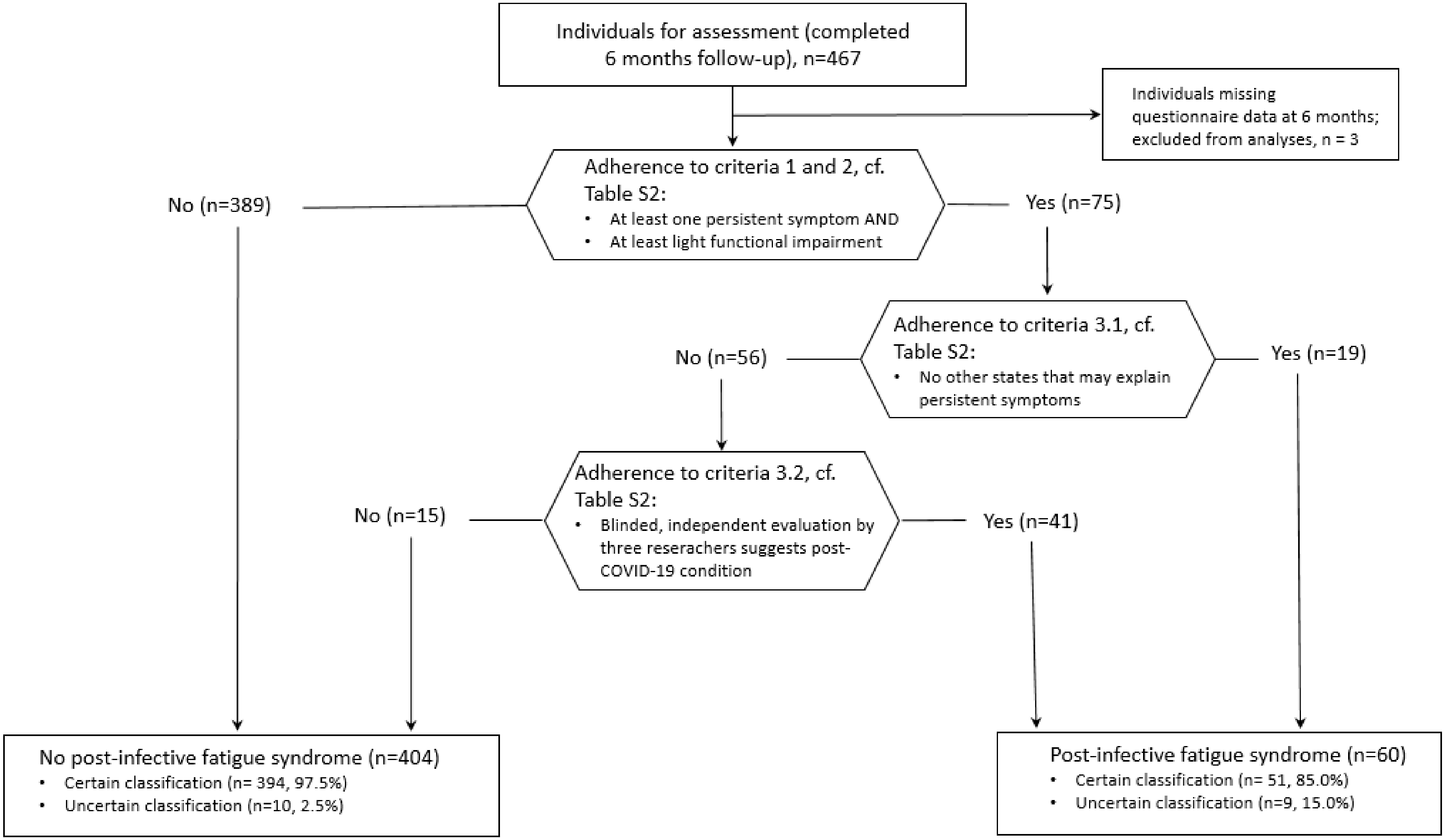

Figure S2. Algorithm for assessment of post-infective fatigue syndrome (PIFS) at six months follow-up.

© 2023 Selvakumar J et al. *JAMA Network Open.*

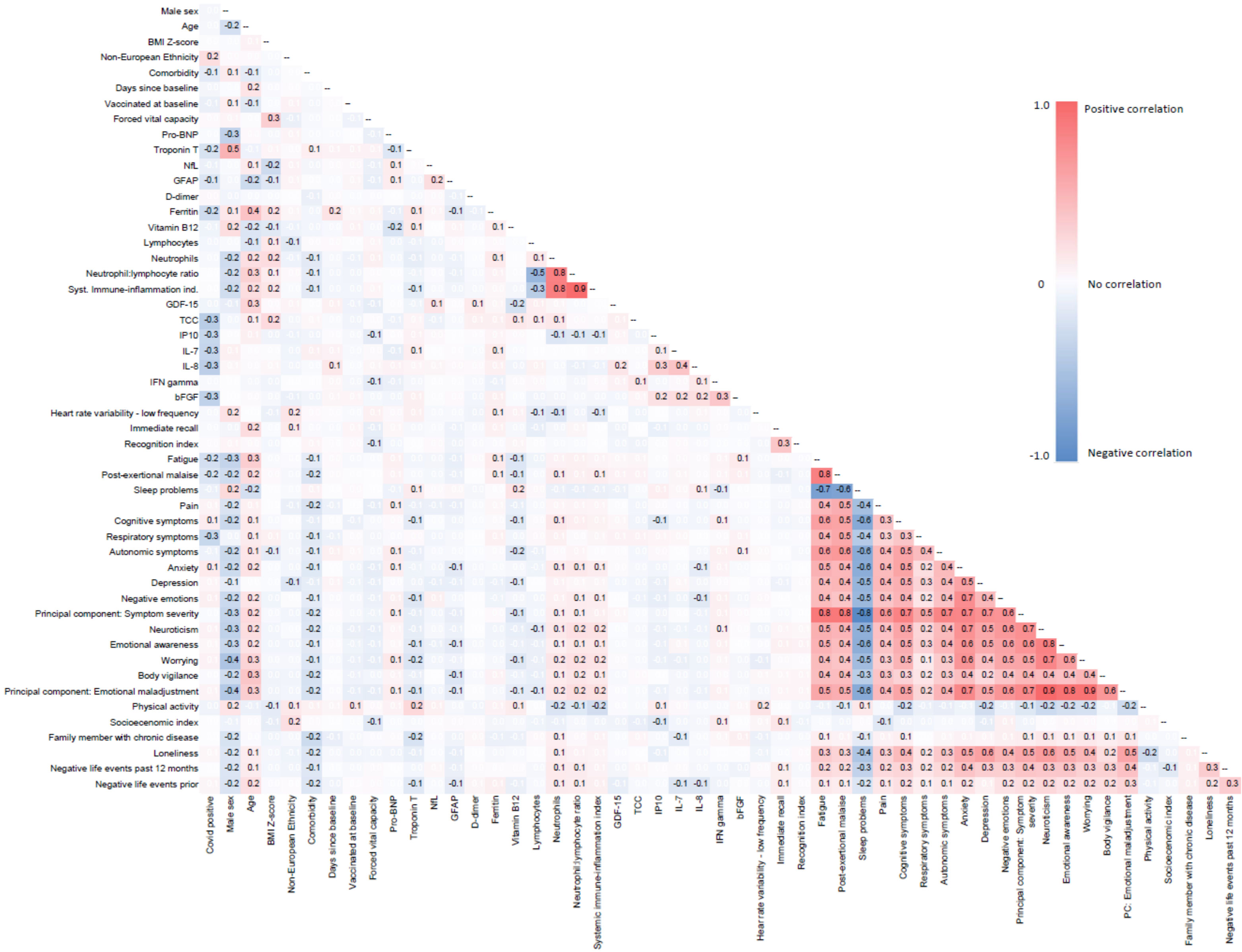

Figure S3. Spearman rank correlation heatmap of independent variables with p<0.25 in bivariate analyses. (*For clarity, coefficients <0.1 are not shown. Full matrix with p-values can be downloaded from [https://1drv.ms/x/s!AopX-j2nV-6e4wrmd9jY6FG53tEl?e=3SjJI7]*)

© 2023 Selvakumar J et al. *JAMA Network Open.*

3. References – supplementary

1 Wyller V. Long-term Effects of COVID-19 in Adolescents: Study protocol and statistical analysis plan. clinicaltrials.gov, 2022 https://clinicaltrials.gov/ct2/show/NCT04686734 (accessed Oct 11, 2022).

2 Group WMGRS, de Onis M. WHO Child Growth Standards based on length/height, weight and age. *Acta Paediatrica* 2006; **95**: 76–85.

3 Graham BL, Steenbruggen I, Miller MR, *et al.* Standardization of Spirometry 2019 Update. An Official American Thoracic Society and European Respiratory Society Technical Statement. *Am J Respir Crit Care Med* 2019; **200**: e70–88.

4 Quanjer PH, Stanojevic S, Cole TJ, *et al.* Multi-ethnic reference values for spirometry for the 3–95-yr age range: the global lung function 2012 equations. *European Respiratory Journal* 2012; **40**: 1324–43.

5 Heart rate variability: standards of measurement, physiological interpretation and clinical use. Task Force of the European Society of Cardiology and the North American Society of Pacing and Electrophysiology. *Circulation* 1996; **93**: 1043–65.

6 Saul J. Beat-To-Beat Variations of Heart Rate Reflect Modulation of Cardiac Autonomic Outflow. *Physiology* 1990; **5**: 32–7.

7 Pagani M, Lombardi F, Guzzetti S, *et al.* Power spectral analysis of heart rate and arterial pressure variabilities as a marker of sympatho-vagal interaction in man and conscious dog. *Circ Res* 1986; **59**: 178–93.

8 Grizzle R. Wechsler Intelligence Scale for Children, Fourth Edition. In: Goldstein S, Naglieri JA, eds. Encyclopedia of child behavior and development. New York: Springer Science+Business Media, 2011.

9 Benedict RHB, Schretlen D, Groninger L, Brandt J. Hopkins Verbal Learning Test – Revised: Normative Data and Analysis of Inter-Form and Test-Retest Reliability. *The Clinical Neuropsychologist* 2010; published online Aug 9. DOI:10.1076/clin.12.1.43.1726.

10 Hughes AM, Chalder T, Hirsch CR, Moss-Morris R. An attention and interpretation bias for illness-specific information in chronic fatigue syndrome. *Psychol Med* 2017; **47**: 853–65.

11 O'Reilly A, Roche B, Gavin A, Ruiz MR, Ryan A, Campion G. A Function Acquisition Speed Test For Equivalence Relations (FASTER). *The Psychological Record* 2013; **63**: 707–24.

12 Bergseth G, Ludviksen JK, Kirschfink M, Giclas PC, Nilsson B, Mollnes TE. An international serum standard for application in assays to detect human complement activation products. *Mol Immunol* 2013; **56**: 232–9.

13 Tran TT, Vaage EB, Mehta A, *et al.* Titers of antibodies the receptor-binding domain (RBD) of ancestral SARS-CoV-2 are predictive for levels of neutralizing antibodies to multiple variants. 2022; : 2022.03.26.484261.

14 Soriano JB, Murthy S, Marshall JC, Relan P, Diaz JV. A clinical case definition of post-COVID-19 condition by a Delphi consensus. *The Lancet Infectious Diseases* 2022; **22**: e102–7.

© 2023 Selvakumar J et al. *JAMA Network Open.*

15 Fukuda K, Straus SE, Hickie I, Sharpe MC, Dobbins JG, Komaroff A. The chronic fatigue syndrome: a comprehensive approach to its definition and study. International Chronic Fatigue Syndrome Study Group. *Ann Intern Med* 1994; **121**: 953–9.

16 University of Oslo. Services for sensitive data (TSD) - University of Oslo. https://www.uio.no/english/services/it/research/sensitive-data/index.html (accessed Oct 13, 2022).

17 Ganzeboom HBG, De Graaf PM, Treiman DJ. A standard international socio-economic index of occupational status. *Social Science Research* 1992; **21**: 1–56.

18 Ganzeboom HBG. A new International Socio-Economic Index (ISEI) of occupational status for the International Standard Classification of Occupation 2008 (ISCO-08) constructed with data from the ISSP 2002-2007. Lisbon, 2010. http://www.harryganzeboom.nl/Pdf/2010%20-%20Ganzeboom-ISEI08-ISSP-Lisbon-(paper).pdf (accessed Jan 18, 2022).

19 Chalder T, Berelowitz G, Pawlikowska T, *et al.* Development of a fatigue scale. *Journal of Psychosomatic Research* 1993; **37**: 147–53.

20 Wagner D, Nisenbaum R, Heim C, Jones JF, Unger ER, Reeves WC. Psychometric properties of the CDC Symptom Inventory for assessment of Chronic Fatigue Syndrome. *Population Health Metrics* 2005; **3**: 8.

21 Jason LA, Sunnquist M. The Development of the DePaul Symptom Questionnaire: Original, Expanded, Brief, and Pediatric Versions. *Frontiers in Pediatrics* 2018; **6**: 330.

22 Bedree H, Sunnquist M, Jason LA. The DePaul Symptom Questionnaire-2: A Validation Study. *Fatigue* 2019; **7**: 166–79.

23 Åkerstedt T, Ingre M, Broman J, Kecklund G. Disturbed Sleep in Shift Workers, Day Workers, and Insomniacs. *Chronobiology International* 2008; **25**: 333–48.

24 Klepstad P, Loge JH, Borchgrevink PC, Mendoza TR, Cleeland CS, Kaasa S. The Norwegian Brief Pain Inventory Questionnaire: Translation and Validation in Cancer Pain Patients. *Journal of Pain and Symptom Management* 2002; **24**: 517–25.

25 Zigmond AS, Snaith RP. The Hospital Anxiety and Depression Scale. *Acta Psychiatrica Scandinavica* 1983; **67**: 361–70.

26 Thompson ER. Development and Validation of an Internationally Reliable Short-Form of the Positive and Negative Affect Schedule (PANAS). *Journal of Cross-Cultural Psychology* 2007; **38**: 227–42.

27 Broadbent E, Petrie KJ, Main J, Weinman J. The Brief Illness Perception Questionnaire. *Journal of Psychosomatic Research* 2006; **60**: 631–7.

28 Varni JW, Burwinkle TM, Seid M, Skarr D. The PedsQL™* 4.0 as a Pediatric Population Health Measure: Feasibility, Reliability, and Validity. *Ambulatory Pediatrics* 2003; **3**: 329–41.

29 Varni JW, Burwinkle TM, Limbers CA, Szer IS. The PedsQL™ as a patient-reported outcome in children and adolescents with fibromyalgia: an analysis of OMERACT domains. *Health Qual Life Outcomes* 2007; **5**: 9.

30 Schmidt NB, Lerew DR, Trakowski JH. Body vigilance in panic disorder: Evaluating attention to bodily perturbations. *Journal of Consulting and Clinical Psychology* 1997; **65**: 214–20.

© 2023 Selvakumar J et al. *JAMA Network Open.*

31 Körner A, Geyer M, Roth M, *et al.* [Personality assessment with the NEO-Five-Factor Inventory: the 30-Item-Short-Version (NEO-FFI-30)]. *Psychother Psychosom Med Psychol* 2008; **58**: 238–45.

32 Pallesen S, Nordhus IH, Carlstedt B, Thayer JF, Johnsen TB. A Norwegian adaptation of the Penn State Worry Questionnaire: factor structure, reliability, validity and norms. *Scand J Psychol* 2006; **47**: 281–91.

33 Bagby RM, Taylor GJ, Parker JD. The Twenty-item Toronto Alexithymia Scale--II. Convergent, discriminant, and concurrent validity. *J Psychosom Res* 1994; **38**: 33–40.

34 Russell D, Peplau LA, Cutrona CE. The revised UCLA Loneliness Scale: concurrent and discriminant validity evidence. *J Pers Soc Psychol* 1980; **39**: 472–80.

35 Romppel M, Herrmann-Lingen C, Wachter R, *et al.* A short form of the General Self-Efficacy Scale (GSE-6): Development, psychometric properties and validity in an intercultural non-clinical sample and a sample of patients at risk for heart failure. *Psychosoc Med* 2013; **10**: Doc01.

36 Flett GL, Hewitt PL, Besser A, *et al.* The Child–Adolescent Perfectionism Scale: Development, Psychometric Properties, and Associations With Stress, Distress, and Psychiatric Symptoms. *Journal of Psychoeducational Assessment* 2016; **34**: 634–52.

37 Power TG. Parenting dimensions and styles: a brief history and recommendations for future research. *Child Obes* 2013; **9 Suppl**: S14-21.

38 Loge JH, Ekeberg Ø, Kaasa S. Fatigue in the general norwegian population: Normative data and associations. *Journal of Psychosomatic Research* 1998; **45**: 53–65.

39 Stavem K, Ghanima W, Olsen MK, Gilboe HM, Einvik G. Prevalence and Determinants of Fatigue after COVID-19 in Non-Hospitalized Subjects: A Population-Based Study. *International Journal of Environmental Research and Public Health* 2021; **18**: 2030.

40 Townsend L, Dyer AH, Jones K, *et al.* Persistent fatigue following SARS-CoV-2 infection is common and independent of severity of initial infection. *PLOS ONE* 2020; **15**: e0240784.

41 Ceban F, Ling S, Lui LMW, *et al.* Fatigue and cognitive impairment in Post-COVID-19 Syndrome: A systematic review and meta-analysis. *Brain, Behavior, and Immunity* 2022; **101**: 93–135.

42 Huang I-C, Thompson LA, Chi Y-Y, *et al.* The Linkage between Pediatric Quality of Life and Health Conditions: Establishing Clinically Meaningful Cutoff Scores for the PedsQL. *Value in Health* 2009; **12**: 773–81.

43 Morriss RK, Wearden AJ. Screening instruments for psychiatric morbidity in chronic fatigue syndrome. *J R Soc Med* 1998; **91**: 365–8.

44 Nordin M, Åkerstedt T, Nordin S. Psychometric evaluation and normative data for the Karolinska Sleep Questionnaire. *Sleep Biol Rhythms* 2013; **11**: 216–26.

45 Nir A, Lindinger A, Rauh M, *et al.* NT-Pro-B-type Natriuretic Peptide in Infants and Children: Reference Values Based on Combined Data from Four Studies. *Pediatr Cardiol* 2008; **30**: 3.

46 American Thoracic Society, American College of Chest Physicians. ATS/ACCP Statement on cardiopulmonary exercise testing. *Am J Respir Crit Care Med* 2003; **167**: 211–77.

© 2023 Selvakumar J et al. *JAMA Network Open.*

47 Sudre CH, Murray B, Varsavsky T, *et al.* Attributes and predictors of long COVID. *Nat Med* 2021; **27**: 626–31.

48 Osmanov IM, Spiridonova E, Bobkova P, *et al.* Risk factors for long covid in previously hospitalised children using the ISARIC Global follow-up protocol: A prospective cohort study. ; : 55.

49 Mohammad S, Aziz R, Al Mahri S, *et al.* Obesity and COVID-19: what makes obese host so vulnerable? *Immun Ageing* 2021; **18**: 1.

50 Thompson EJ, Williams DM, Walker AJ, *et al.* Long COVID burden and risk factors in 10 UK longitudinal studies and electronic health records. *Nat Commun* 2022; **13**: 3528.

51 Hickie I, Davenport T, Wakefield D, *et al.* Post-infective and chronic fatigue syndromes precipitated by viral and non-viral pathogens: prospective cohort study. *BMJ* 2006; **333**: 575.

52 Ayoubkhani D, Bermingham C, Pouwels KB, *et al.* Trajectory of long covid symptoms after covid-19 vaccination: community based cohort study. *BMJ* 2022; **377**: e069676.

53 Townsend L, Fogarty H, Dyer A, *et al.* Prolonged elevation of D-dimer levels in convalescent COVID-19 patients is independent of the acute phase response. *Journal of Thrombosis and Haemostasis* 2021; **19**: 1064–70.

54 Pretorius E, Vlok M, Venter C, *et al.* Persistent clotting protein pathology in Long COVID/Post-Acute Sequelae of COVID-19 (PASC) is accompanied by increased levels of antiplasmin. *Cardiovascular Diabetology* 2021; **20**: 172.

55 García-Abellán J, Padilla S, Fernández-González M, *et al.* Long-term clinical, virological and immunological outcomes in patients hospitalized for COVID-19: antibody response predicts long COVID. 2021; : 2021.03.08.21253124.

56 Satterfield BA, Bhatt DL, Gersh BJ. Cardiac involvement in the long-term implications of COVID-19. *Nat Rev Cardiol* 2021; : 1–10.

57 Havdal LB, Berven LL, Selvakumar J, *et al.* Neurological Involvement in COVID-19 Among Non-Hospitalized Adolescents and Young Adults. *Front Neurol* 2022; **13**: 915712.

58 Kanberg N, Ashton NJ, Andersson L-M, *et al.* Neurochemical evidence of astrocytic and neuronal injury commonly found in COVID-19. *Neurology* 2020; **95**: e1754–9.

59 Virhammar J, Nääs A, Fällmar D, *et al.* Biomarkers for central nervous system injury in cerebrospinal fluid are elevated in COVID-19 and associated with neurological symptoms and disease severity. *European Journal of Neurology* 2021; **28**: 3324–31.

60 Pedersen M, Asprusten TT, Godang K, *et al.* Predictors of chronic fatigue in adolescents six months after acute Epstein-Barr virus infection: A prospective cohort study. *Brain, Behavior, and Immunity* 2019; **75**: 94–100.

61 Akbarialiabad H, Taghrir MH, Abdollahi A, *et al.* Long COVID, a comprehensive systematic scoping review. *Infection* 2021; **49**: 1163–86.

62 Mazza MG, De Lorenzo R, Conte C, *et al.* Anxiety and depression in COVID-19 survivors: Role of inflammatory and clinical predictors. *Brain, Behavior, and Immunity* 2020; **89**: 594–600.

© 2023 Selvakumar J et al. *JAMA Network Open.*

63 Myhre PL, Prebensen C, Strand H, *et al.* Growth Differentiation Factor 15 Provides Prognostic Information Superior to Established Cardiovascular and Inflammatory Biomarkers in Unselected Patients Hospitalized With COVID-19. *Circulation* 2020; **142**: 2128–37.

64 Lund Berven L, Selvakumar J, Havdal L, *et al.* Inflammatory Markers, Pulmonary Function, and Clinical Symptoms in Acute COVID-19 Among Non-Hospitalized Adolescents and Young Adults. *Front Immunol* 2022; **13**: 837288.

65 Schultze JL, Aschenbrenner AC. COVID-19 and the human innate immune system. *Cell* 2021; **184**: 1671–92.

66 Fevang B, Wyller VBB, Mollnes TE, *et al.* Lasting Immunological Imprint of Primary Epstein-Barr Virus Infection With Associations to Chronic Low-Grade Inflammation and Fatigue. *Frontiers in Immunology* 2021; **12**. https://www.frontiersin.org/article/10.3389/fimmu.2021.715102 (accessed Jan 24, 2022).

67 Ling L, Chen Z, Lui G, *et al.* Longitudinal Cytokine Profile in Patients With Mild to Critical COVID-19. *Frontiers in Immunology* 2021; **12**. https://www.frontiersin.org/article/10.3389/fimmu.2021.763292 (accessed Jan 21, 2022).

68 Kempuraj D, Selvakumar GP, Ahmed ME, *et al.* COVID-19, Mast Cells, Cytokine Storm, Psychological Stress, and Neuroinflammation. *Neuroscientist* 2020; **26**: 402–14.

69 Peluso MJ, Lu S, Tang AF, *et al.* Markers of immune activation and inflammation in individuals with post-acute sequelae of SARS-CoV-2 infection. 2021; : 2021.07.09.21260287.

70 Blomberg B, Mohn KG-I, Brokstad KA, *et al.* Long COVID in a prospective cohort of home-isolated patients. *Nat Med* 2021; **27**: 1607–13.

71 Cervia C, Zurbuchen Y, Taeschler P, *et al.* Immunoglobulin signature predicts risk of post-acute COVID-19 syndrome. *Nat Commun* 2022; **13**: 446.

72 Sante GD, Buonsenso D, Rose CD, *et al.* Immune profile of children with post-acute sequelae of SARS-CoV-2 infection (Long Covid). 2021.

73 Queiroz MAF, Neves PFM das, Lima SS, *et al.* Cytokine Profiles Associated With Acute COVID-19 and Long COVID-19 Syndrome. *Frontiers in Cellular and Infection Microbiology* 2022; **12**. https://www.frontiersin.org/articles/10.3389/fcimb.2022.922422 (accessed Sept 1, 2022).

74 Lorkiewicz P, Waszkiewicz N. Biomarkers of Post-COVID Depression. *Journal of Clinical Medicine* 2021; **10**: 4142.

75 Klein J, Wood J, Jaycox J, *et al.* Distinguishing features of Long COVID identified through immune profiling. 2022; : 2022.08.09.22278592.

76 Patterson BK, Guevara-Coto J, Yogendra R, *et al.* Immune-Based Prediction of COVID-19 Severity and Chronicity Decoded Using Machine Learning. *Frontiers in Immunology* 2021; **12**. https://www.frontiersin.org/articles/10.3389/fimmu.2021.700782 (accessed Sept 2, 2022).

77 Kedor C, Freitag H, Meyer-Arndt L, *et al.* A prospective observational study of post-COVID-19 chronic fatigue syndrome following the first pandemic wave in Germany and biomarkers associated with symptom severity. *Nat Commun* 2022; **13**: 5104.

© 2023 Selvakumar J et al. *JAMA Network Open.*

78 Acosta-Ampudia Y, Monsalve DM, Rojas M, *et al.* Persistent Autoimmune Activation and Proinflammatory State in Post-Coronavirus Disease 2019 Syndrome. *The Journal of Infectious Diseases* 2022; **225**: 2155–62.

79 Kirkham BW, Kavanaugh A, Reich K. Interleukin-17A: a unique pathway in immune-mediated diseases: psoriasis, psoriatic arthritis and rheumatoid arthritis. *Immunology* 2014; **141**: 133–42.

80 Smadja DM, Mentzer SJ, Fontenay M, *et al.* COVID-19 is a systemic vascular hemopathy: insight for mechanistic and clinical aspects. *Angiogenesis* 2021; **24**: 755–88.

81 Fogarty H, Townsend L, Morrin H, *et al.* Persistent endotheliopathy in the pathogenesis of long COVID syndrome. *Journal of Thrombosis and Haemostasis* 2021; **19**: 2546–53.

82 Nazarinia D, Behzadifard M, Gholampour J, Karimi R, Gholampour M. Eotaxin-1 (CCL11) in neuroinflammatory disorders and possible role in COVID-19 neurologic complications. *Acta Neurol Belg* 2022; **122**: 865–9.

83 Phetsouphanh C, Darley DR, Wilson DB, *et al.* Immunological dysfunction persists for 8 months following initial mild-to-moderate SARS-CoV-2 infection. *Nat Immunol* 2022; **23**: 210–6.

84 Schultheiß C, Willscher E, Paschold L, *et al.* The IL-1β, IL-6, and TNF cytokine triad is associated with post-acute sequelae of COVID-19. *Cell Reports Medicine* 2022; **3**: 100663.

85 Kristiansen MS, Stabursvik J, O'Leary EC, *et al.* Clinical symptoms and markers of disease mechanisms in adolescent chronic fatigue following Epstein-Barr virus infection: An exploratory cross-sectional study. *Brain Behav Immun* 2019; **80**: 551–63.

86 Crook H, Raza S, Nowell J, Young M, Edison P. Long covid—mechanisms, risk factors, and management. *BMJ* 2021; **374**: n1648.

87 Sandler CX, Wyller VBB, Moss-Morris R, *et al.* Long COVID and Post-infective Fatigue Syndrome: A Review. *Open Forum Infectious Diseases* 2021; **8**: ofab440.

88 Brodwall EM, Pedersen M, Asprusten TT, Wyller VBB. Pain in adolescent chronic fatigue following Epstein-Barr virus infection. *Scand J Pain* 2020; **20**: 765–73.

89 Jason LA, Cotler J, Islam MF, Sunnquist M, Katz BZ. Risks for Developing Myalgic Encephalomyelitis/Chronic Fatigue Syndrome in College Students Following Infectious Mononucleosis: A Prospective Cohort Study. *Clin Infect Dis* 2021; **73**: e3740–6.

90 Russell C, Wearden AJ, Fairclough G, Emsley RA, Kyle SD. Subjective but Not Actigraphy-Defined Sleep Predicts Next-Day Fatigue in Chronic Fatigue Syndrome: A Prospective Daily Diary Study. *Sleep* 2016; **39**: 937–44.

91 Wang S, Quan L, Chavarro JE, *et al.* Associations of Depression, Anxiety, Worry, Perceived Stress, and Loneliness Prior to Infection With Risk of Post–COVID-19 Conditions. *JAMA Psychiatry* 2022; published online Sept 7. DOI:10.1001/jamapsychiatry.2022.2640.

92 Hulme K, Hudson JL, Rojczyk P, Little P, Moss-Morris R. Biopsychosocial risk factors of persistent fatigue after acute infection: A systematic review to inform interventions. *Journal of Psychosomatic Research* 2017; **99**: 120–9.

93 Moss-Morris R, Petrie KJ, Weinman J. Functioning in chronic fatigue syndrome: Do illness perceptions play a regulatory role? *British Journal of Health Psychology* 1996; **1**: 15–25.

© 2023 Selvakumar J et al. *JAMA Network Open.*

94 White PD, Thomas JM, Kangro HO, *et al.* Predictions and associations of fatigue syndromes and mood disorders that occur after infectious mononucleosis. *The Lancet* 2001; **358**: 1946–54.

95 Kadota Y, Cooper G, Burton AR, *et al.* Autonomic hyper-vigilance in post-infective fatigue syndrome. *Biological Psychology* 2010; **85**: 97–103.

96 La J, Bz K, Y S, Cj M, Y I, R T. Predictors of Post-Infectious Chronic Fatigue Syndrome in Adolescents. *Health psychology and behavioral medicine* 2014; **2**. DOI:10.1080/21642850.2013.869176.

97 Novotný JS, Gonzalez-Rivas JP, Kunzová Š, *et al.* Risk Factors Underlying COVID-19 Lockdown-Induced Mental Distress. *Front Psychiatry* 2020; **11**: 603014.

98 Nugawela MD, Stephenson T, Shafran R, *et al.* Developing a model for predicting impairing physical symptoms in children 3 months after a SARS-CoV-2 PCR-test: The CLoCk Study. 2022; : 2022.04.01.22273117.

99 Caspersen IH, Magnus P, Trogstad L. Excess risk and clusters of symptoms after COVID-19 in a large Norwegian cohort. *Eur J Epidemiol* 2022; published online Feb 25. DOI:10.1007/s10654-022-00847-8.

100 Desgranges F, Tadini E, Munting A, *et al.* Post-COVID-19 Syndrome in Outpatients: a Cohort Study. *J Gen Intern Med* 2022; published online March 22. DOI:10.1007/s11606-021-07242-1.

101 Gherardi RK, Crépeaux G, Authier F-J. Myalgia and chronic fatigue syndrome following immunization: macrophagic myofasciitis and animal studies support linkage to aluminum adjuvant persistency and diffusion in the immune system. *Autoimmun Rev* 2019; **18**: 691–705.

© 2023 Selvakumar J et al. *JAMA Network Open.*
