# Supplementary material for: Prevalence and Characteristics Associated With Post–COVID-19 Condition Among Nonhospitalized Adolescents and Young Adults
Source: JAMA Netw Open. 2023 Mar 30;6(3):e235763. doi: 10.1001/jamanetworkopen.2023.5763 (PMC10064252; doi:10.1001/jamanetworkopen.2023.5763)
Supplement: Supplement 2. — Data Sharing Statement [file jamanetwopen-e235763-s002.pdf]

# Data Sharing Statement

Selvakumar. Prevalence and Characteristics Associated With Post-COVID-19 Condition Among Nonhospitalized Adolescents and Young Adults. *JAMA Netw Open*. Published March 30, 2023. doi:10.1001/jamanetworkopen.2023.5763

**Data**
**Data available:** Yes
**Data types:** Deidentified participant data, Data dictionary
**How to access data:** Anonymised individual participant data as well as data dictionary will be made available with publication for scientific purposes upon reasonable request. Proposals might also need approval by the appropriate Research Ethics committee. The Services for Sensitive Data at the University of Oslo will be used as the data sharing platform (https://www.uio.no/english/services/it/research/sensitive-data/index.html); access to the designated server area will be granted by the principal investigator (email: v.b.b.wyller@medisin.uio.no). The study protocol (encompassing the informed consent form) as well as the statistical analysis plan is freely available from the designated ClinicalTrial registry website (https://www.clinicaltrials.gov/ct2/show/NCT04686734).
**When available:** With publication

**Supporting Documents**
**Document types:** Informed consent form, Other (please specify)
**Additional Information:** Statistical analysis plan, prespecified outcomes, informed consent forms are available at https://clinicaltrials.gov/ct2/show/NCT04686734
**How to access documents:** https://clinicaltrials.gov/ct2/show/NCT04686734
**When available:** beginning date: 01-31-2021

**Additional Information**
**Who can access the data:** Researchers whose proposed use of the data has been approved.
**Types of analyses:** Research purposes
**Mechanisms of data availability:** After approval of a proposal, the Services for Sensitive Data at the University of Oslo will be used as the data sharing platform (https://www.uio.no/english/services/it/research/sensitive-data/index.html); access to the designated server area will be granted by the principal investigator (email: v.b.b.wyller@medisin.uio.no).
